# Systematic exploration of eczema‐associated paediatric diseases in a Chinese population of millions: A retrospective observation study

**DOI:** 10.1002/clt2.12249

**Published:** 2023-05-02

**Authors:** Huiwen Zheng, Jian Yang, Yuqing Feng, Huilong Duan, Lizhong Du, Qiang Shu, Haomin Li

**Affiliations:** ^1^ Children's Hospital Zhejiang University School of Medicine National Clinical Research Center for Child Health Hangzhou China; ^2^ College of Biomedical Engineering and Instrument Science Zhejiang University Hangzhou China

**Keywords:** atopic dermatitis, atopic march, disease association, eczema, paediatric diseases

## Abstract

**Background:**

Eczema is the most common form of dermatitis and also the starting point of atopic march. Although many eczema‐associated allergic and immunologic disorders have been studied, there remains a gap in the systematic quantitative knowledge regarding the relationships between all childhood disorders and eczema. This study aimed to systematically explore eczema‐associated childhood diseases using a real‐world, long‐term clinical dataset generated from millions of children in China.

**Methods:**

Data were collected at 8,907,735 outpatient healthcare visits from 2,592,147 children between January 1, 2013, and August 15, 2019, at the largest comprehensive pediatric medical center in Zhejiang Province of China. The period prevalence differences in various pediatric diseases between children with and without eczema were used to test the independence of various pediatric disorders and eczema using Fisher's exact test. Bonferroni correction was used to adjust the *p* value in multiple testing. Odds ratio >2 with 95% confidence interval not including 1 and adjusted *p* < 0.05 was used to identify eczema‐associated diseases.

**Results:**

Overall, 234 pediatric disorders were identified from more than 6000 different pediatric disorders. An interactive eczema‐associated disease map that has related quantitative epidemiological features called ADmap was published at http://pedmap.nbscn.org/admap. Thirty‐six of these disease associations have not been reported in previous studies.

**Conclusion:**

This systematic exploratory study confirmed the associations of many well‐known diseases with eczema in Chinese children and also identified some novel and interesting associations. These results are valuable for the development of a comprehensive approach to the management of eczema in childhood.

## 1 INTRODUCTION

Eczema is the most common form of dermatitis, affecting 245 million people globally in 2015, mostly children and adolescents.^1^ Atopic dermatitis (AD), which is the most common type of eczema, has a wide range incidence among different countries and periods of observation.^2^, ^3^ In the United States, eczema affects approximately 10%–30% of people.^4^ In China, the prevalence of AD among children aged 1–7 years is 12.94%.^5^ A systematic review showed that the incidence and prevalence of eczema have increased globally.^6^, ^7^

The association between eczema and other diseases has been known for a long time.^8^ Genetic and environmental factors are thought to play a role in the pathogenesis of eczema.^9^ Approximately 50% of patients with severe AD will develop asthma, and 75% will develop allergic rhinitis.^10^ People with eczema may also be particularly susceptible to bacterial, viral, and fungal skin infections.^11^ Many other associated diseases have also been reported, such as conjunctivitis,^12^ eosinophilic oesophagitis (EOE),^13^ anemia,^14^ cardiovascular diseases,^15^ and psychosocial diseases.^16^ To date, most studies have focused on several predefined diseases with relatively high prevalence rates. Most of these association studies were conducted in Western countries. We observed some potential associations in a previous study exploring relationships among common paediatric disorders using clinical data.^17^ In this study, we systematically explored eczema‐associated paediatric disorders using longer, larger, real‐world clinical data generated from a children's hospital in China.

## 2 METHODS

### 2.1 Data and patients

This study was approved by the Institutional Review Board of the Children's Hospital of Zhejiang University School of Medicine, with a waiver for informed consent, as the utilization of anonymized retrospective data does not require patient consent under local legislation.

We collected data obtained at outpatient healthcare visits between January 1, 2013, and August 15, 2019, at the Children's Hospital, Zhejiang University School of Medicine. Given its status as the center of children's healthcare in Zhejiang Province and the National Clinical Research Center for children's health and diseases in China, the number of daily outpatient visits in this hospital often exceeds 10,000 visits. Because patients do not require a referral to visit children's hospitals as outpatients in China, the disease spectrum of outpatients is an adequate reflection of the disease spectrum of the population of children in this area. The data were obtained from an EHR database generated from 8,907,735 outpatient visits (mean age at visit 44.65 ± 41.98 months) by 2,592,147 paediatric patients with 6882 types of disorders during the study period. These diagnoses were recorded by clinicians in the EHR system based on the clinical guideline at that time.

### 2.2 Statistical analyses

Among these 2,592,147 paediatric patients, 91,515 children had been diagnosed with eczema at least once. In all these diagnoses of eczema, 91.1% were made by dermatologists, 2.9% by pediatricians, and the rest by various specialties such as ophthalmologists, ophthalmologists, urologists, and allergists. The incidences of different disorders in children with/without eczema were calculated. As for many disorders with relatively low prevalence, Fisher's exact tests were used to determine whether there were nonrandom associations between the disease and eczema. For each paediatric disorder, 6881 disease eczema pairs were tested based on the children's disease and eczema status. Bonferroni correction was used to adjust the *p* value in multiple testing.

Diseases positively associated with eczema (positive diseases) were defined by the following criteria: odds ratio (OR) >2, 95% confidence interval (CI) not including 1, adjusted *p* value of Fisher's exact test <0.05, and a visiting rate among eczema patients (the number of patients with eczema and visit for this specific diseases/the total number of patients with eczema) > 0.0001 to ensure that each subset was large enough to achieve sufficient statistical power to detect a difference between patients with and without eczema. All statistics and calculations were performed in the *R* (v3.4.0) environment. The mean and standard deviation of age of the positive diseases in the group with and without eczema were also calculated. The novelty of associations was determined by a literature search in the PubMed database.

Many rare diseases with a prevalence of less than 1 in 10,000 people have also been reported to have an eczema phenotype. We also explored rare eczema‐associated diseases using RDmap,^18^ which is a novel rare disease knowledge base developed by our group that can identify rare diseases based on standard phenotype terms. Four phenotypes (HP:0000964 eczema; HP:0000976 eczematoid dermatitis; HP:0001047 AD; and HP:0011127 perioral eczema) were used to search for rare diseases in the RDmap. The rare diseases with an age of onset in the antenatal, neonatal and childhood were included.

### 2.3 Patient and public involvement

Patient and public were not involved in any way in this study.

## 3 RESULTS

**FIGURE 1:**
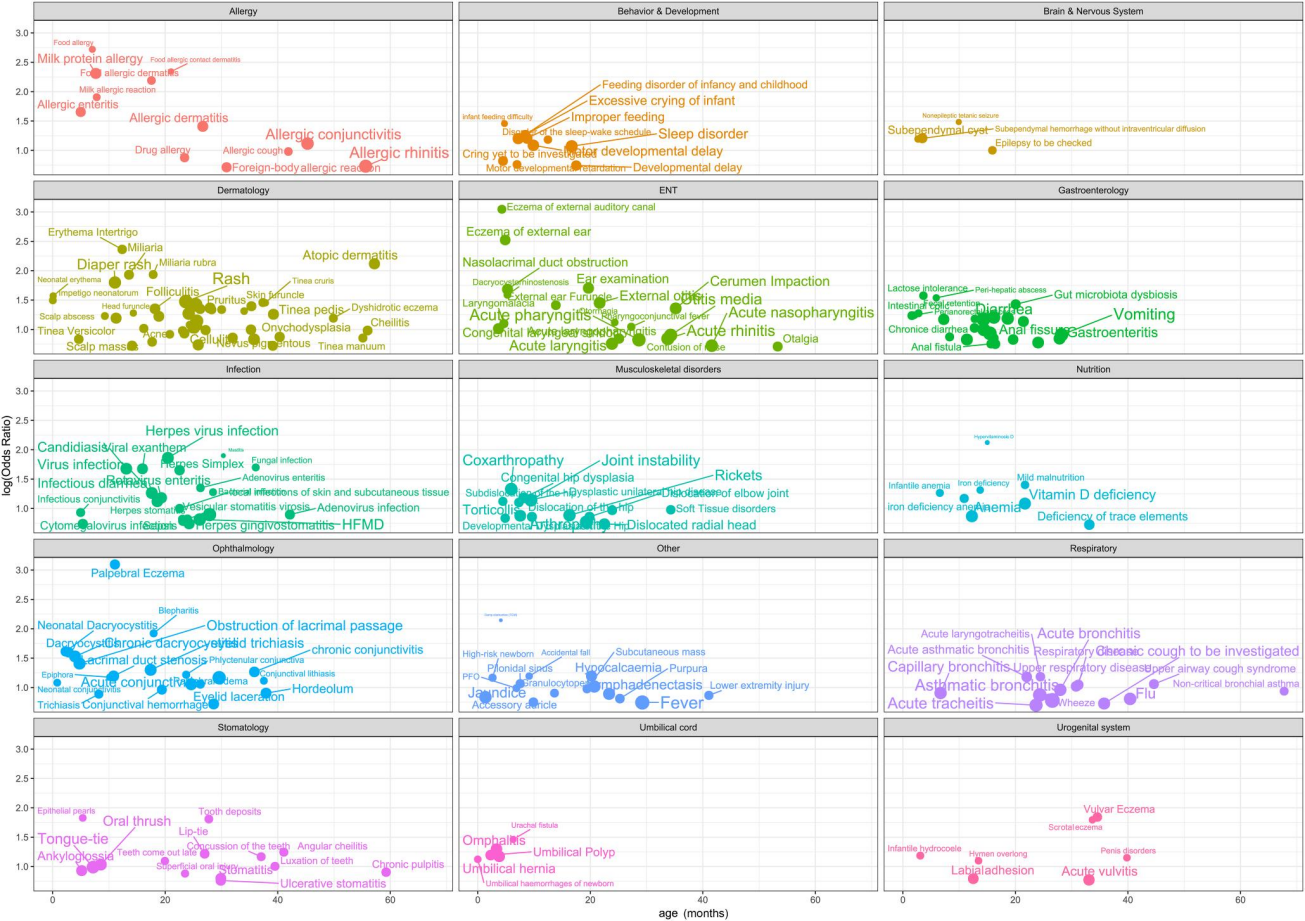
Eczema‐associated paediatric disorders grouped by disease. The mean age affected by the associated diseases and log of odds ratio (OR) are plotted on the *x* and *y* axes, respectively. The size of the bubble represents the incidence of the disease in children with eczema.

Among more than 6000 paediatric disorders, 234 diseases were identified as being potentially associated with eczema. The details of all 234 associated diseases are listed in supplemental Table S1 and shown in an interactive map called ADmap published at http://pedmap.nbscn.org/admap. Fifty common nondermatological paediatric disorders with OR>3 and adjusted *p* value <0.05 are listed in Table 1. These 234 eczema‐associated paediatric disorders were plotted and grouped by disease type in Figure 1. The details of each disease group are discussed below, and an OR >3 is indicated as the OR [95% CI]. The background of thousands of paediatric disorders were shown in the supplemental Table S2.

**TABLE 1:** The common paediatric disorders (excluding dermatologic conditions) associated with eczema.

Paediatric disorders	Odds ratio (OR) [95% CI]	Visiting rate^a^	Visiting rate^b^	Age^a^(months)	Age^b^(months)
Milk protein allergy	10.08 [8.87–11.42]	0.00037	0.00372	8.2 ± 7.3	7.7 ± 6.2
Herpes virus infection	6.43 [5.96–6.93]	0.00147	0.00940	33.3 ± 37.7	20.5 ± 24.6
Candidiasis	5.35 [4.93–5.80]	0.00148	0.00786	19.2 ± 27.3	13.1 ± 17.4
Viral exanthem	5.34 [4.70–6.06]	0.00061	0.00322	20.1 ± 20.2	16.0 ± 12.1
Allergic enteritis	5.23 [4.37,6.23]	0.00032	0.00167	7.5 ± 15.3	5.0 ± 5.6
Herpes simplex	5.22 [4.45–6.09]	0.00041	0.00212	45.2 ± 61.3	22.6 ± 20.4
Neonatal dacryocystitis	5.02 [4.21–5.96]	0.00034	0.00170	2.2 ± 7.9	2.3 ± 9.9
Dacryocystitis	4.95 [4.24–5.77]	0.00044	0.00216	4.3 ± 7.1	2.8 ± 2.2
Chronic dacryocystitis	4.62 [4.27–4.98]	0.00187	0.00858	4.9 ± 8.0	4.0 ± 5.7
Gut microbiota dysbiosis	4.17 [3.46–4.99]	0.00036	0.00152	35.6 ± 75.1	20.0 ± 31.1
Obstruction of lacrimal passage	4.09 [3.82–4.37]	0.00262	0.01061	6.2 ± 7.7	4.8 ± 4.7
Adenovirus enteritis	3.86 [2.79–5.23]	0.00014	0.00052	27.0 ± 18.5	26.3 ± 16.1
Coxarthropathy	3.78 [3.60–3.97]	0.00574	0.02135	7.9 ± 12.4	6.0 ± 6.9
Eyelid trichiasis	3.67 [3.41–3.95]	0.00244	0.00889	29.4 ± 27.9	17.4 ± 18.9
Omphalitis	3.65 [3.35–3.98]	0.00177	0.00644	5.8 ± 17.8	3.3 ± 8.6
Feeding disorder of infancy and childhood	3.55 [2.90–4.30]	0.00036	0.00129	11.6 ± 15.4	8.5 ± 8.3
Chronic conjunctivitis	3.55 [3.09–4.06]	0.00074	0.00262	44.7 ± 28.4	35.8 ± 26.3
Virus infection	3.54 [3.29–3.80]	0.00268	0.00942	20.5 ± 28.9	17.6 ± 30.0
Angular cheilitis	3.47 [2.63–4.51]	0.00020	0.00070	54.3 ± 36.9	41.1 ± 25.2
Intestinal colic	3.44 [2.58–4.52]	0.00019	0.00064	3.7 ± 11.7	1.6 ± 2.0
Phlyctenular conjunctiva	3.38 [2.32–4.79]	0.00012	0.00039	40.1 ± 29.0	23.7 ± 17.9
Lip‐tie	3.38 [2.72–4.16]	0.00033	0.00110	33.9 ± 27.7	27.0 ± 26.0
Subependymal cyst	3.35 [2.81–3.97]	0.00050	0.00166	11.0 ± 45.7	3.5 ± 4.7
Diarrhoea	3.34 [3.19–3.49]	0.00748	0.02452	22.2 ± 27.9	16.2 ± 15.5
Improper feeding	3.33 [2.93–3.78]	0.00092	0.00307	11.2 ± 10.0	8.8 ± 6.2
Excessive crying of infant	3.32 [2.99–3.68]	0.00139	0.00459	6.6 ± 7.4	7.2 ± 7.6
Lacrimal duct stenosis	3.30 [2.96–3.68]	0.00126	0.00415	15.9 ± 18.3	10.8 ± 8.9
Umbilical polyp	3.30 [2.85–3.81]	0.00072	0.00236	4.0 ± 28.7	2.3 ± 10.8
Hypocalcaemia	3.30 [2.95–3.69]	0.00115	0.00380	34.1 ± 38.5	20.2 ± 27.5
Infantile hydrocoele	3.28 [2.20–4.74]	0.00011	0.00035	9.3 ± 20.0	3.1 ± 3.0
Acute enteritis	3.27 [3.10–3.46]	0.00497	0.01607	24.8 ± 30.4	18.6 ± 17.1
Acute laryngotracheitis	3.27 [2.62–4.04]	0.00032	0.00106	31.3 ± 25.0	24.4 ± 23.0
Disorder of the sleep‐wake schedule	3.26 [2.32–4.49]	0.00014	0.00047	24.6 ± 26.8	12.5 ± 6.5
Rotavirus enteritis	3.26 [2.95–3.59]	0.00155	0.00505	20.5 ± 19.3	19.3 ± 3.4
Congenital hip dysplasia	3.26 [2.86–3.70]	0.00089	0.00291	10.3 ± 12.8	8.2 ± 7.0
Acute asthmatic bronchitis	3.26 [2.83–3.73]	0.00079	0.00257	23.5 ± 19.4	22.0 ± 14.2
Intestinal malabsorption	3.25 [2.28–4.53]	0.00013	0.00044	15.1 ± 18.6	12.8 ± 10.2
Dyspepsia	3.23 [3.16–3.30]	0.03414	0.10248	22.2 ± 29.7	14.3 ± 17.8
Umbilical hernia	3.23 [2.93–3.56]	0.00159	0.00511	5.9 ± 15.6	3.8 ± 8.7
Perianal abscess	3.22 [2.90–3.57]	0.00141	0.00455	11.1 ± 24.5	7.3 ± 16.5
Conjunctivitis	3.21 [3.12–3.31]	0.01934	0.05955	43.8 ± 42.1	29.6 ± 29.5
Concussion of the teeth	3.21 [2.41–4.21]	0.00020	0.00064	53.2 ± 32.7	37.1 ± 24.5
High‐risk newborn	3.21 [2.22–4.52]	0.00013	0.00040	2.5 ± 11.9	2.6 ± 1.8
Joint instability	3.13 [3.01–3.26]	0.00980	0.03006	14.0 ± 20.0	9.5 ± 11.2
Gastrointestinal dysfunction	3.11 [2.76–3.50]	0.00111	0.00344	37.4 ± 36.8	21.4 ± 20.8
Infectious diarrhoea	3.07 [2.81–3.34]	0.00211	0.00646	20.7 ± 28.8	18.6 ± 11.7
Subdislocation of the hip	3.07 [2.36,3.92]	0.00025	0.00078	10.1 ± 17.3	4.5 ± 3.7
Allergic conjunctivitis	3.05 [2.90–3.21]	0.00645	0.01942	58.4 ± 36.9	45.3 ± 28.2
Conjunctival lithiasis	3.05 [2.02–4.45]	0.00011	0.00033	46.1 ± 28.4	37.5 ± 22.2
Dysplastic unilateral hip disease	3.01 [2.41–3.72]	0.00035	0.00105	10.2 ± 15.1	7.3 ± 5.7

^a^ Visiting rate and age in 2500632 children without eczema.

^b^ Visiting rate and age in 91515 children with eczema.

### 3.1 Allergies

Many well‐known allergic diseases, such as food allergies (OR 15.18 [8.33~26.94]), milk protein allergies (OR 10.08 [8.87~11.42]), and milk allergic reactions (OR 6.72 [5.46~18.91]), were identified in the early stage of children with eczema. Gastrointestinal forms of food allergies, such as allergic enteritis (OR 5.23 [4.37~6.23]), also often occur in younger infants. Children with eczema experienced more drug allergies and foreign body allergic reactions than children without eczema. Other allergic disorders, such as allergic dermatitis (OR 4.09 [3.62~4.61]), allergic conjunctivitis (OR 3.05 [2.90~3.21]) and allergic rhinitis, affected children at subsequent ages.

### 3.2 Infection

Children with eczema were also susceptible to bacterial infection (OR 3.58 [2.40~5.20]), viral infection (OR 3.54 [3.29~3.80]), and particularly fungal infection (OR 5.46 [3.64~7.98]). The most significant infection affected by eczema was mastitis (OR 6.68 [3.12~13.12]), which is a relatively rare condition in children. Among viral infections, herpesvirus infection (OR 6.43 [5.96~6.93]), viral exanthems (OR 5.34 [4.70~6.06]), herpes simplex (OR 5.22 [4.45~6.09]), adenovirus and cytomegalovirus infections were all associated with eczema. Among fungal infections, candidiasis was more prevalent in children with eczema (OR 5.35 [4.93~5.80]).

### 3.3 Ear, nose, and throat diseases

Ear eczema most often led to problems in the ear canal, such as external otitis (OR 4.27 [3.94~4.62]), external ear furuncles (OR 4.11 [3.33~5.03]), and cerumen impaction (OR 3.89 [3.60~4.20]). Itchy skin also leads to scratching, which may have caused otorrhagia (OR 3.06 [1.99~4.53]) and otalgia. Two outstanding disorders, that is, nasolacrimal duct obstruction (OR 5.384 [4.77~6.06]) and nasolacrimal duct stenosis (OR 4.90 [3.22~7.21]), mainly affected babies aged under 6 months. Laryngomalacia (OR 3.00 [2.32~3.83]) was also identified as associated with eczema.

### 3.4 Respiratory diseases

Many common respiratory diseases were identified in this study, such as acute asthmatic bronchitis (OR 3.26 [2.83~3.73]) and acute laryngotracheitis (OR 3.27 [2.62~4.04]). Noncritical bronchial asthma, upper airway cough syndrome, acute bronchitis, and chronic cough were also identified as being associated with eczema in this study. We also observed that critical bronchial asthma (OR 27.33 [1.98~375.01]) had a significantly higher OR in the eczema population, but the incidence of this condition was too low to meet the criteria.

### 3.5 Ophthalmic diseases

The part of the skin most affected by eczema was the eyelids (palpebral eczema OR 22.09 [17.82~27.36]). Lacrimal duct stenosis (OR 3.30 [2.96~3.68]) or obstruction (OR 4.09 [3.82~4.37]) was increased in small infants with eczema, and they also caused a high incidence of epiphora (OR 3.24 [2.05~4.91]) and dacryocystitis (OR 4.95 [4.24~5.77]). Another common eye problem in patients with eczema was conjunctivitis (OR 3.21 [3.12~3.31]). Many different conjunctivitis types, such as neonatal conjunctivitis (OR 3.95 [1.95~4.31]), allergic conjunctivitis (OR 3.05 [2.90~3.21]), and chronic conjunctivitis (OR 3.55 [3.09~4.06]), were associated with eczema. Due to such chronic conjunctival inflammation conditions, the prevalence of conjunctival lithiasis (OR 3.05 [2.02~4.45]) was also increased in children with eczema. Some injuries or infections, such as eyelid trichiasis (OR 3.67 [3.41~3.95]), eyelid laceration and blepharitis (OR 6.83 [4.52~10.07]), may have been caused by itching and scratching due to eyelid eczema.

### 3.6 Stomatological diseases

Eczema is also associated with many common oral conditions. First, eczema increased the risk of many oral infections, such as oral thrush, angular cheilitis (OR 3.47 [2.63~4.51]) and stomatitis. Unexpectedly, many tooth problems, such as tooth concussion (OR 3.21 [2.41~4.21]), tooth deposits (OR 6.10 [4.43~8.27]), and epithelial pearls (OR 6.22 [3.97~9.44]), were correlated with eczema. Moreover, two types of ties, tongue‐tie and lip‐tie, were identified.

### 3.7 Musculoskeletal disorders

Unusual associations were observed between several arthropathies, such as coxarthropathy (OR 3.78 [3.60~3.97]) and joint instability (OR 3.13 [3.01~3.26]), and eczema. Several joint dislocation and development problems, such as subdislocation of the hip (OR 3.07 [2.36~3.92]), congenital hip dysplasia (OR 3.26 [2.86~3.70]), dysplastic unilateral hip disease (OR 3.01 [2.41~3.72]), and rickets, were associated with eczema.

### 3.8 Gastrointestinal diseases

Common paediatric digestive system conditions, such as dyspepsia (OR 3.23 [3.15~3.30]) and diarrhoea (OR 3.34 [3.19~3.49]), were associated with eczema in children. The most highly correlated gastrointestinal disease was lactose intolerance (OR 4.83 [3.42~6.68]). Inflammatory conditions of the alimentary tract, such as acute enteritis (OR 3.27 [3.10~3.46]), rotavirus enteritis (OR 3.26 [2.95~3.59]), and adenovirus enteritis (OR 3.86 [2.79~5.23]), were more common in children with eczema. These disorders are associated with many symptoms, such as vomiting, gastroesophageal reflux, changes in defecation habits, spastic colitis, intestinal colic (OR 3.44 [2.58~4.52]), and faecal retention (OR 3.58 [2.41~5.16]). These long‐term gastrointestinal problems may cause gut microbiota dysbiosis (OR 4.17 [3.46~4.99]) and gastrointestinal dysfunction (OR 3.11 [2.76~3.50]) later in life.

### 3.9 Dermatologic diseases

Some synonymous terms and similar skin conditions, such as AD (OR 8.32 [7.41~9.32]), dermatitis (OR 4.14 [4.05~4.23]), and seborrheic dermatitis (OR 3.86 [3.26~4.53]), were identified as being associated with eczema. Some eczema‐related symptoms, such as erythema intertrigo (OR 10.62 [8.20~13.67]) and pruritus (OR 4.04 [3.35~4.83]), were also noted in the results. Other common skin problems, such as miliaria (OR 6.89 [5.63~8.38]), miliaria rubra (OR 6.92 [5.12~9.22]), diaper rash (OR 6.04 [5.70~6.40]) and rash (OR 4.37 [4.26~4.48]), were associated with eczema. As children with eczema seems to be more prone to viral, bacterial and fungal infections, conditions such as impetigo neonatorum (OR 4.46 [2.95~6.54]), impetigo (OR 3.40 [2.99~3.85]), molluscum contagiosum (OR 3.91 [3.48~4.39]), exanthema subitem (OR 3.29 [2.94~3.68]), folliculitis (OR 3.86 [3.37~4.40]), granuloma (OR 3.71 [2.41~5.53]) and various tineas were identified as being associated with eczema. Infants with neonatal erythema (OR 4.81 [2.91~7.61]) at an early age were more likely to experience eczema later in life. Most other common skin problems, such as urticaria (OR 3.14 [3.03~3.25]) and popular urticaria (OR 3.56 [3.38~3.74]), were associated with eczema. Unexpectedly, children with eczema were more likely to develop frostbite (OR 3.80 [2.97~4.81]).

### 3.10 Brain and nervous system diseases

The prevalence rates of nonepileptic seizure (OR 4.41 [2.37~7.65]) were increased in patients with eczema. Due to such symptoms, a significant proportion of children with eczema experience epilepsy. However, epilepsy was negatively associated with eczema in this study, with an OR of 0.30 [0.26–0.34]. Subependymal cysts (OR 3.35 [2.81~3.97]) and subependymal haemorrhage without intraventricular diffusion (OR 3.30 [2.09~5.01]), which are related to intrauterine infection in newborns, were more common in patients with eczema. These nervous conditions mostly occur in the early stage of infancy.

### 3.11 Nutritional problems

The prevalence of mild malnutrition (OR 4.06 [2.90~5.57]) in children with eczema was significantly increased. The prevalence rates of vitamin D deficiency and related hypervitaminosis D (OR 8.35 [3.83~16.77]) due to treatment were also notably increased. The prevalence of anaemia, especially infantile anaemia (OR 3.54 [2.42~5.05]), was increased in children with eczema. Anaemia was mostly caused by iron deficiency (OR 3.72 [2.37~5.62]). These deficiencies in trace elements and malnutrition were most likely caused by the large number of gastrointestinal problems associated with eczema.

### 3.12 Umbilical disorders

The results revealed a cluster of conditions related to the umbilical cord. The incidence of urachal fistula (OR 4.32 [2.59~6.87]) was unexpectedly increased in patients with eczema. Several other umbilical conditions, such as omphalitis (OR 3.65 [3.35~3.98]), umbilical polyps (OR 3.30 [2.85~3.81]), umbilical hernia (OR 3.23 [2.93~3.56]), and umbilical haemorrhage in newborns (OR 3.08 [1.97~4.62]), were also identified as being associated with eczema.

### 3.13 Urogenital disorders

The vulva and scrotum were also highly affected by eczema. Scrotal eczema (OR 6.02 [3.70~9.43]) and vulvar eczema (OR 6.32 [4.97~7.95]) were identified in the results. Furthermore, infantile hydrocoele (OR 3.28 [2.20~4.74]) and other penis disorders (3.16 [2.02~4.75]) such as leukoplakia of penis, balanoposthitis, inflammatory disorders of penis, ulcer of penis were identified as being associated with eczema in boys. Labial adhesion and overlong hymen were identified in girls.

### 3.14 Behaviour and developmental disorders

Due to allergies to food, itchy skin and other comorbidities, many infants with eczema display some abnormal behaviours, such as feeding difficulty (OR 4.29 [2.50~7.00]), feeding disorders of infancy and childhood (OR 3.55 [2.90~4.30]), excessive crying of infants (OR 3.32 [3.00~3.68]), and disorder of the sleep‐wake schedule (OR 3.26 [2.32~4.49]). Infants with eczema are at a high risk of motor developmental delay and developmental index delay.

### 3.15 Other disorders

The prevalence of some common symptoms in children, such as fever and jaundice, was increased in children with eczema. It appears that high‐risk newborns (OR 3.21 [2.22~4.52]) are more likely to develop eczema. Unlike most other accidental injuries, accidental falls (OR 3.3 [2.09~5.01]) and lower extremity injuries were identified more frequently in children with eczema. Another highly correlated condition was a traditional Chinese medicine condition called 湿阻(damp obstruction) (OR 8.54 [3.74~17.81]), which means that dampness invaded the body, obstructed the channels and interfered with the blood and Qi circulation.

### 3.16 Novel associations identified in this study

Many associations between eczema and other diseases have been reported in previous studies shown as the reference list at the end of supplemental Table S1. We still identified 36 novel associations that have never been reported and cannot be adequately explained based on our current knowledge, as listed in Table 2. Most of the novel associations with relatively low prevalence focus on arthropathy, umbilical disorders and various ties in the mouth. Compared with children without eczema, these associated diseases always onset earlier in children with eczema.

**TABLE 2:** Novel identified associations in this study.

Paediatric disorders	Odds ratio [95% CI]	Visiting rate^a^	Visiting rate^b^	Age^a^(months)	Age^b^(months)
Damp obstruction (TCM)	8.54 [3.74–17.81]	0.00001	0.00011	30.5 ± 44.1	4.1 ± 3.0
Epithelial pearls	6.22 [3.97–9.44]	0.00005	0.00031	5.0 ± 8.2	5.3 ± 6.4
Urachal fistula	4.32 [2.59–6.87]	0.00005	0.00023	9.8 ± 22.6	6.3 ± 10.2
Frostbite	3.80 [2.97–4.81]	0.00023	0.00087	37.9 ± 35.5	28.3 ± 26.6
Coxarthropathy	3.78 [3.60–3.97]	0.00574	0.02135	7.9 ± 12.4	6.0 ± 6.9
Lip‐tie	3.38 [2.72–4.16]	0.00033	0.00110	33.9 ± 27.7	27.0 ± 26.0
Subependymal cyst	3.35 [2.81–3.97]	0.00050	0.00166	11.0 ± 45.7	3.5 ± 4.7
Accidental fall	3.30 [2.09–5.01]	0.00008	0.00027	39.1 ± 38.1	9.1 ± 5.8
Subependymal haemorrhage without intraventricular diffusion	3.30 [2.09–5.01]	0.00008	0.00027	3.3 ± 19.8	2.7 ± 7.3
Umbilical polyp	3.30 [2.85–3.81]	0.00072	0.00236	4.0 ± 28.7	2.3 ± 10.8
Infantile hydrocoele	3.28 [2.20–4.74]	0.00011	0.00035	9.3 ± 19.9	3.1 ± 3.0
Congenital hip dysplasia	3.26 [2.86–3.70]	0.00089	0.00291	10.3 ± 12.8	8.2 ± 7.0
Umbilical hernia	3.23 [2.93–3.56]	0.00159	0.00511	5.9 ± 15.6	3.8 ± 8.7
High‐risk newborn	3.21 [2.22–4.52]	0.00013	0.00040	2.5 ± 11.9	2.6 ± 1.8
Joint instability	3.13 [3.01–3.26]	0.00980	0.03006	14.0 ± 20.0	9.5 ± 11.2
Umbilical haemorrhages of newborn	3.08 [1.97–4.62]	0.00009	0.00028	0.0 ± 0.1	0.0 ± 0.0
Subdislocation of the hip	3.07 [2.36–3.92]	0.00025	0.00078	10.1 ± 17.3	4.5 ± 3.7
Dysplastic unilateral hip disease	3.01 [2.41–3.72]	0.00035	0.00105	10.2 ± 15.1	7.3 ± 5.7
Laryngomalacia	3.00 [2.32–3.83]	0.00027	0.00080	5.1 ± 7.1	4.6 ± 5.2
Hymen overlong	3.00 [1.92–4.50]	0.00009	0.00028	23.4 ± 32.4	13.4 ± 22.1
Pilonidal sinus	2.90 [2.25–3.70]	0.00028	0.00080	12.0 ± 21.7	7.5 ± 12.5
Neoplasm of skin	2.85 [1.83–4.28]	0.00010	0.00028	41.5 ± 37.65	25.9 ± 21.17
Congenital laryngeal stridor	2.75 [2.46–3.08]	0.00139	0.00381	4.3 ± 6.2	3.7 ± 3.3
Tongue‐tie	2.69 [2.57–2.82]	0.00853	0.02265	16.0 ± 20.4	7.2 ± 10.2
PFO	2.69 [1.78–3.92]	0.00012	0.00033	13.4 ± 18.9	6.9 ± 4.7
Dislocations, sprains and strains	2.64 [2.17–3.19]	0.00049	0.00129	31.6 ± 24.4	23.9 ± 15.5
Ankyloglossia	2.54 [2.25–2.85]	0.00134	0.00339	14.9 ± 27.6	5.2 ± 7.9
Torticollis	2.40 [2.22–2.59]	0.00338	0.00809	15.0 ± 24.0	7.5 ± 8.3
Lower extremity injury	2.38 [1.84–3.03]	0.00033	0.00079	64.0 ± 44.4	41.1 ± 33.4
Dislocation of the hip	2.36 [1.97–2.80]	0.00066	0.00155	18.0 ± 24.5	9.6 ± 11.9
Dislocation of elbow joint	2.36 [1.99–2.77]	0.00073	0.00173	26.7 ± 18.2	19.9 ± 11.7
Developmental dysplasia of the hip	2.30 [1.83–2.87]	0.00041	0.00094	10.3 ± 15.8	4.9 ± 3.4
Arthropathy	2.16 [2.08–2.24]	0.01696	0.03590	40.1 ± 43.3	19.3 ± 22.5
Accessory auricle	2.12 [1.71–2.59]	0.00053	0.00111	20.7 ± 25.7	9.9 ± 13.4
Dislocated radial head	2.09 [1.89–2.29]	0.00247	0.00514	26.7 ± 17.4	22.5 ± 13.6

^a^ Visiting rate and age in 2500632 children without eczema.

^b^ Visiting rate and age in 91515 children with eczema.

### 3.17 Age distributions of eczema‐associated diseases in paediatric patients

**FIGURE 2:**
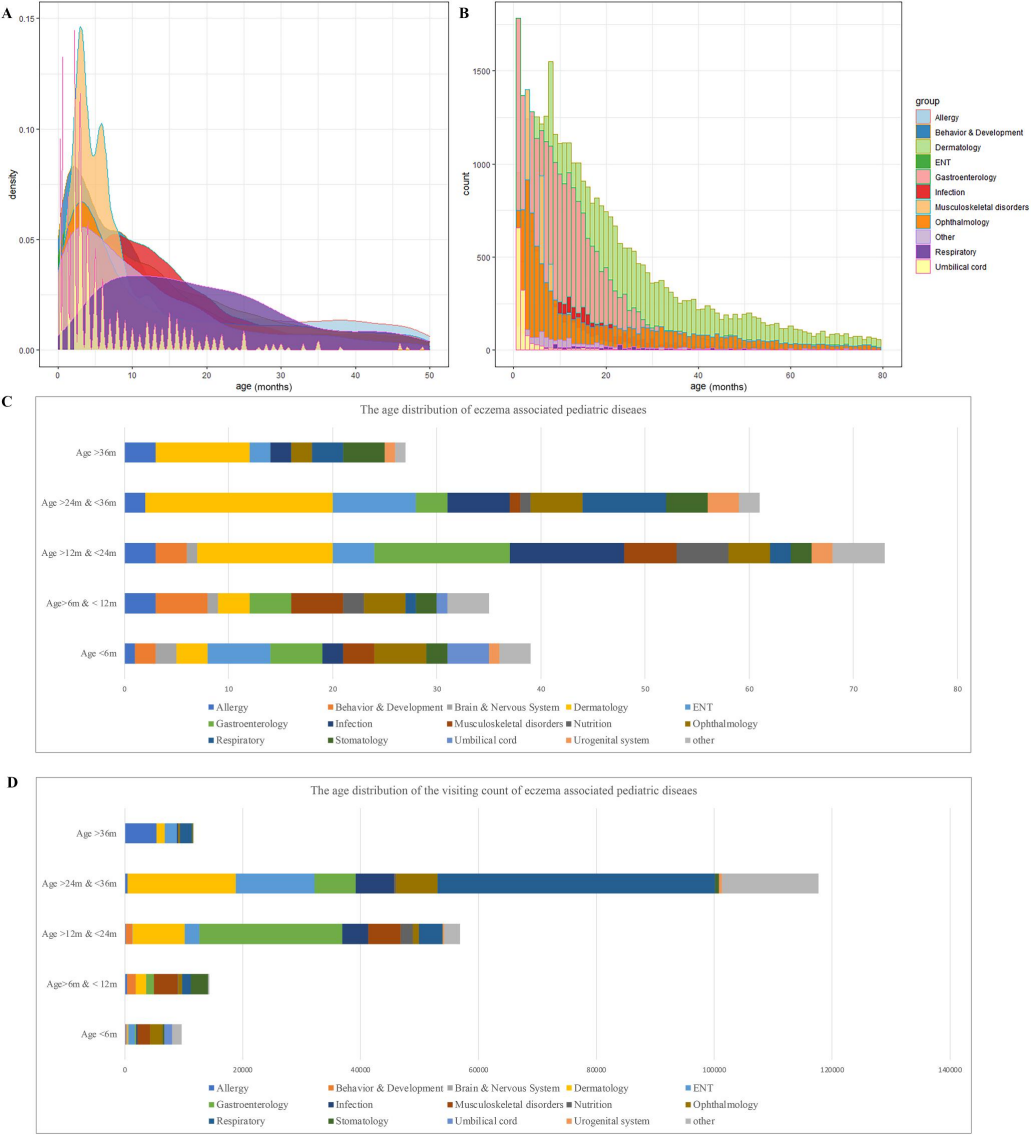
Age distribution of diseases associated with eczema. The associations with a high odds ratio (OR) (>3) account for 96460 visits, which was used to generate (A) and (B). (A) The age distribution density of different disease groups. (B) The absolute count of visits for different ages from different disease groups. All associations were grouped based on their mean age, and children with eczema were used to generate (C) and (D). (C) The age distribution of all 235 diseases in different age groups. (C) The age distribution of the visiting count of these diseases in children with eczema.

To further understand the distribution of the eczema‐associated diseases, a subdataset comprising data from 96,460 visits by children with eczema who were also diagnosed with diseases with a high OR (>3) was used to generate Figure 2A,B. These disease groups had different age distributions, as shown in Figure 2A, and most of them affected children in their first year. Based on the absolute counts shown in Figure 2B, dermatologic, gastroenterologic and ophthalmic complaints were the most common causes of visits of children with eczema. Based on the mean age of children affected by the diseases, all of these associated diseases were grouped by age and plotted in Figure 2C,D. Many relatively rare diseases were identified at the age <6 m, and many common respiratory and gastroenterology disorders contributed to the major visit count between 1 and 3 years. Since the length of data collection for this study was 7 years, theoretically if the time difference between the onset of the associated disease and eczema was more than 7 years, then no such association could be found in the data of this study.

### 3.18 Rare diseases associated with eczema

To compensate for the lack of rare disease cases in this observational study, as well as to gain a more systematic understanding of eczema‐associated diseases, total 43 rare diseases were identified with eczema phenotype using phenotype query on RDmap and in which 37 rare diseases with an age of onset in antenatal, neonatal and childhood are listed in Table 3. It should be noted that this part of the disease association was not strictly analyzed statistically, but only indicates that the eczema phenotype has a relatively high frequency of occurrence in these rare diseases. The phenotypes that concomitant with eczema in rare diseases were sorted list in supplemental Table S3 based on its frequency. In addition to abnormalities of the integument and immune system, these phenotypes are associated with abnormalities of the nervous system, growth, musculature, the teeth, and the skeletal and digestive systems.

**TABLE 3:** Paediatric rare diseases associated with eczema.

Disease name	Prevalence	Eczema	Gene
Holocarboxylase synthetase deficiency (OMIM: 253270)	<1/200 000	***	HLCS
Netherton syndrome (OMIM: 256500)	1–9/1 000 000	***	SPINK5
Ehlers‐Danlos syndrome, classic type (OMIM: 130000)	1–9/100 000	***	COL5A1; COL5A2; COL1A1
Bullous pemphigoid	1–5/10 000	***	HLA‐DRB1; HLA‐DQB1
Grubben‐de Cock‐Borghgraef syndrome (OMIM: 233810)	<1/1 000 000	***	
Autosomal dominant hyper‐IgE syndrome (OMIM: 147060)	1–9/100 000	***	STAT3
Hereditary acrokeratotic poikiloderma, Weary type	<1/1 000 000	***	
Combined immunodeficiency due to DOCK8 deficiency (OMIM: 243700)	1/1 000 000	***	DOCK8
Xq12‐q13.3 duplication syndrome	<1/1 000 000	***	
Intellectual disability‐seizures‐macrocephaly‐obesity syndrome	<1/1 000 000	***	
Craniosynostosis‐anal anomalies‐porokeratosis syndrome (OMIM: 603116)	<1/1 000 000	***	
Shwachman‐Diamond syndrome (OMIM: 260400)	1–9/1 000 000	**	SBDS; EFL1; SRP54; DNAJC21;
Epidermolytic palmoplantar keratoderma (OMIM: 144200)	Not yet documented	**	KRT1; KRT9; KRT16;
2q37 microdeletion syndrome (OMIM: 600430)	Unknown	**	HDAC4
Ichthyosis follicularis‐alopecia‐photophobia syndrome (OMIM: 308205)	<1/1 000 000	**	MBTPS2
Pili torti‐onychodysplasia syndrome	<1/1 000 000	**	
Intellectual disability‐sparse hair‐brachydactyly syndrome (OMIM: 601358)	<1/1 000 000	**	SMARCA2
Biotinidase deficiency (OMIM: 253260)	1–9/100 000	**	BTD
Classic phenylketonuria (OMIM: 261600)	1/15,000 births	**	PAH
Maternal uniparental disomy of chromosome 6	<1/1 000 000	**	
Hypohidrotic ectodermal dysplasia (OMIM: 129490)	1–9/100 000	**	EDAR; EDARADD; TRAF6; KDF1; WNT10A; IKBKG; NFKBIA; EDA; EDA2R;
Acral peeling skin syndrome (OMIM: 609796)	<1/1 000 000	**	CSTA; TGM5;
Dubowitz syndrome (OMIM: 223370)	Unknown	**	LIG4; NSUN2;
Autoimmune enteropathy and endocrinopathy‐susceptibility to chronic infections syndrome (OMIM: 614162)	Not yet documented	**	STAT1
Dystrophic epidermolysis bullosa (OMIM: 604129)	1–9/1 000 000	*	COL7A1
Chronic granulomatous disease (OMIM: 233670)	1–9/1 000 000	*	CYBB; NCF1; NCF2; CYBA; NCF4; CYBC1
Wiskott‐Aldrich syndrome (OMIM: 301000)	1–9/1 000 000	*	WAS; WIPF1;
Cranio‐osteoarthropathy (OMIM: 259100)	<1/1 000 000	*	HPGD
Autosomal dominant hypohidrotic ectodermal dysplasia (OMIM: 129490)	<1/1 000 000	*	EDAR; EDARADD; TRAF6; KDF1;
Jacobsen syndrome (OMIM: 147791)	Unknown	*	FLI1
Pachydermoperiostosis (OMIM: 167100)	Unknown	*	SLCO2A1; HPGD
X‐linked intellectual disability‐hypogonadism‐ichthyosis‐obesity‐short stature syndrome	<1/1 000 000	*	
Autosomal erythropoietic protoporphyria (OMIM: 177000)	1–9/1 000 000	*	FECH
Ectodermal dysplasia syndrome	6–9/10 000	*	
Distal trisomy 5q	Not yet documented	*	
Elastoderma	<1/1 000 000	*	
8q21.11 microdeletion syndrome (OMIM: 614230)	<1/1 000 000	*	

*Note*: ***very frequent (99%–80%) **frequent (79%–30%) *occasional (29%–5%).

## 4 DISCUSSION

Although eczema itself accounts for only 1.30% of all visits in our study population, the number of visits of children with eczema exceeds 8.44% of total visits. Children with eczema had an average of 8.22 outpatient visits for various problems during the study period, while the corresponding number for children without eczema was only 3.26. Understanding these disease associations and providing preemptive health management are expected to significantly reduce the need for paediatric resources.

As the results show, many paediatric disorders are significantly associated with eczema. Some may have similar genetic mechanisms, such as dental problems in early childhood^19^; some may be susceptible to common immune deficiencies, such as various infections; and some may be indirectly caused by associated health problems, such as behavioural, nutritional and developmental problems. There are also many unknown relationships among these associations. Many paediatric joint disorders were identified in the clinical data. A possible explanation is that treatments of these disorders, such as long‐term cast immobilization, may trigger or exacerbate eczema. While we checked the visiting date of patients (*n* = 3285) with both eczema and arthropathy, which was the most prevalent disorder in this group, the eczema was diagnosed 130 days before the arthropathy on average (IQR [−245.88, 46.91]), which means that most of the eczema cannot be explained as triggered by related treatments. Furthermore, as many rare genetic disorders coexist with joint development problems and eczema (shown in supplemental Table S3 and Figure S1), we cannot rule out the possibility that the underlying mechanism that triggers eczema affects joint development.

Although the pathogenesis of AD involves a complex interplay of epidermal barrier dysfunction and dysregulated immune response, the increasing importance of vitamin D deficiency (OR 2.95 [2.76~3.16]) in atopic individuals has to be highlighted.^20^ Vitamin D and its analogues seem to play an increasing role in the management of diseases such as AD.^21^ However, the optimal dose, duration and effect of vitamin D supplementation have not been well studied. The risk of hypervitaminosis D (OR 8.35 [3.83~16.77]) is significantly higher in children with eczema, suggesting that there are problems with current vitamin D supplementation strategies.

A large epidemiological study showed that eczema was strongly associated with autism and attention deficit hyperactivity disorder (ADHD).^22^ However, there were negative associations in our dataset, including autism, with an OR of 0.67, and ADHD, with an OR of 0.43. The different prevalence rates of autism and ADHD, the specific service availability, and the diagnosis age may have also contributed to these inconsistent results. Some epidemiologic studies on the association between allergic disease and epilepsy in adults and children have come to conflicting results.^23^ The findings of this study are also very interesting. Nonepileptic seizures (OR 4.41 [2.37~7.65]) were increased in patients with eczema, while epilepsy (OR 0.30 [0.26~0.34]) was decreased. The relationship between eczema and seizures should be further studied in data with better disease classifications and final prognoses.

In addition to the positive associations, there were several negative associations. However, most of them were adolescent disorders, which can be explained by the time spacing between disease and eczema onset beyond the time span of this study. However, there were still several neurological diseases with unexpectedly low ORs, such as facial neuritis (OR 0.27), facial paralysis (OR 0.45), dysarthria (OR 0.12), and dyslalia (OR 0.43). A potential explanation is that the disruption of the skin barrier by scratching exacerbates inflammation, and such inflammation promotes nerve growth. A study also showed that the distribution density of cutaneous nerve fibres was much higher in AD patients than in normal controls.^24^

The coronavirus disease 2019 (COVID‐19) pandemic and associated measures, such as home quarantine and more frequent hand washing, may have exacerbated eczema and affected the frequency of paediatric visits.^25^ This study period ended in the summer of 2019 and partially reduced the impact of the COVID‐19 pandemic.

There are some limitations of this study that should be noted. First, the willingness of outpatient visits to this hospital for different diseases may have affected the results. These may lead to sample selection that does not accurately reflect the target population. Some conditions that are more likely to come to this hospital may have their OR overestimated; others that are not likely to come to this hospital may have their OR underestimated. Especially, when the prevalence of a disease is particularly low and its willingness to attend is particularly influenced by other confounding factors, it may be misleading to the results of this study. This potential ‘selection bias’ are of concern but given that this is the most comprehensive and widely served children's specialty hospital in the region and that the study used strict statistical criteria and sample size requirement, we believe that these reported results are still significant. Second, as the diagnostic terms used by clinicians are not well controlled in practice, synonyms may have been used by different clinicians or during different periods. Third, although the time span of the study cohort was more than 7 years, some diseases, such as adolescent disorders that may affect children with eczema after 7 years or more, their associations may not have been captured in this dataset and were not identified. The median age of identified positive diseases is 32 months with IQR 16–43. Some diseases that may be diagnosed at a high age, such as autism and ADHD, may also have been affected by this issue. Considering the age distribution of eczema, the results of this study may not cover all age groups of paediatric diseases from 0 to 18 years. A final limitation is that the association found here does not effectively account for the causal relationship between the two diseases. Further research needs to be able to control for confounders such as age, gender, and willingness to attend if the causal relationship between these diseases is to be clarified. The strength of this study is that it systematically explored all paediatric disorders that are associated with eczema from numerous different clinical cases in Chinese children and rare diseases knowledge base. Some of these strong correlations have never been reported in previous studies.

In summary, this is the first systematic exploration of associations between paediatric disorders and eczema using a long‐term, large clinical dataset in China. More than 200 different paediatric disorders were identified, which confirmed many well‐known diseases concomitant with eczema and also introduced some novel and interesting associations. These results are valuable for the development of a comprehensive approach to the management of eczema for children.

## AUTHOR CONTRIBUTIONS

Haomin Li and Huiwen Zheng conceptualized and designed the study, collected data, carried out the initial analyses, drafted the initial manuscript, and reviewed and revised the manuscript. Jian Yang, Yuqing Feng, and Huilong Duan collected data and carried out the initial analyses. Lizhong Du and Qiang Shu coordinated and supervised the data collection and critically reviewed the manuscript for important intellectual content. All authors approved the final manuscript as submitted and agree to be accountable for all aspects of the work.

## CONFLICT OF INTEREST STATEMENT

The authors have no conflicts of interest relevant to this article to disclose.

## Supporting information

Supporting Information S1
