# Supplementary material for: Systematic exploration of eczema‐associated paediatric diseases in a Chinese population of millions: A retrospective observation study
Source: Clin Transl Allergy. 2023 May 2;13(5):e12249. doi: 10.1002/clt2.12249 (PMC10154876; doi:10.1002/clt2.12249)
Supplement: Supplementary file 1 — Supporting Information S1 [file CLT2-13-e12249-s001.docx]

**Supplemental materials**

**Table S1 The whole list of significant associations between pediatric disorders and eczema.** The disorder with citations is a reported association that can be queried from the literature; # represents an association that is not clearly reported in the literature but can be directly explained from the available evidence through relevant common sense and medical knowledge; * represents an novel association that is never reported in the literature and at the same time cannot be adequately explained by the available evidence.

| **Disease Groups** | **Pediatric Disorders** | **Odds Ratio (OR)** | **95% CI.l** | **95% CI.h** | **Bonferroni-corrected**  **p-value** | **Visiting Rate**  **_without eczema_** | **Visiting Rate _with eczema_** | **Age**  (months)  **_without eczema_** | **Age**  (months)  **_with eczema_** |
| --- | --- | --- | --- | --- | --- | --- | --- | --- | --- |
| Allergy | Food allergy ^1^ | 15.18 | 8.33 | 26.94 | *** | 0.00001 | 0.00022 | 8.3 | 7.1 |
|  | Food allergic contact dermatitis ^1^ | 10.41 | 5.46 | 18.91 | *** | 0.00002 | 0.00017 | 21.1 | 21.0 |
|  | Milk protein allergy ^1^ | 10.08 | 8.87 | 11.42 | *** | 0.00037 | 0.00372 | 8.2 | 7.7 |
|  | Food allergic dermatitis ^1^ | 8.94 | 6.38 | 12.33 | *** | 0.00006 | 0.00056 | 21.8 | 17.6 |
|  | Milk allergic reaction ^1^ | 6.72 | 4.39 | 10.03 | *** | 0.00005 | 0.00034 | 8.1 | 7.8 |
|  | Allergic enteritis ^2^ | 5.23 | 4.37 | 6.23 | *** | 0.00032 | 0.00167 | 7.5 | 5.0 |
|  | Allergic dermatitis ^3^ | 4.09 | 3.62 | 4.61 | *** | 0.00083 | 0.00340 | 58.9 | 26.7 |
|  | Allergic conjunctivitis ^4^ | 3.05 | 2.90 | 3.21 | *** | 0.00645 | 0.01942 | 58.4 | 45.3 |
|  | Allergic cough ^5^ | 2.67 | 1.96 | 3.56 | *** | 0.00021 | 0.00056 | 52.4 | 41.9 |
|  | Drug allergy ^6^ | 2.40 | 1.87 | 3.04 | *** | 0.00034 | 0.00082 | 40.0 | 23.4 |
|  | Allergic rhinitis ^7^ | 2.08 | 2.01 | 2.15 | *** | 0.01914 | 0.03896 | 70.9 | 55.6 |
|  | Foreign-body allergic reaction ^8^ | 2.04 | 1.71 | 2.41 | *** | 0.00078 | 0.00160 | 42.4 | 30.9 |
| Behavior & Development | infant feeding difficulty ^9^ | 4.29 | 2.50 | 7.00 | ** | 0.00005 | 0.00021 | 6.3 | 4.8 |
|  | Feeding disorder of infancy and childhood ^10^ | 3.55 | 2.90 | 4.30 | *** | 0.00036 | 0.00129 | 11.6 | 8.5 |
|  | Improper feeding ^11^ | 3.33 | 2.93 | 3.78 | *** | 0.00092 | 0.00307 | 11.2 | 8.8 |
|  | Excessive crying of infant ^#^ | 3.32 | 2.99 | 3.68 | *** | 0.00139 | 0.00459 | 6.6 | 7.2 |
|  | Disorder of the sleep-wake schedule ^12^ | 3.26 | 2.32 | 4.49 | *** | 0.00014 | 0.00047 | 24.6 | 12.5 |
|  | Motor developmental delay ^13^ | 2.96 | 2.71 | 3.24 | *** | 0.00202 | 0.00597 | 13.2 | 9.9 |
|  | Sleep disorder ^12^ | 2.92 | 2.72 | 3.13 | *** | 0.00338 | 0.00980 | 44.5 | 16.7 |
|  | Cring yet to be investigated ^#^ | 2.27 | 1.87 | 2.74 | *** | 0.00058 | 0.00131 | 8.1 | 4.5 |
|  | Motor developmental retardation ^13^ | 2.14 | 1.59 | 2.82 | ** | 0.00028 | 0.00060 | 12.5 | 7.0 |
|  | Developmental index delay ^14^ | 2.10 | 1.79 | 2.45 | *** | 0.00090 | 0.00189 | 33.9 | 17.5 |
| Brain or Nervous System | Nonepileptic tetanic seizure ^15^ | 4.41 | 2.37 | 7.65 | * | 0.00004 | 0.00016 | 10.4 | 9.9 |
|  | Subependymal cyst * | 3.35 | 2.81 | 3.97 | *** | 0.00050 | 0.00166 | 11.0 | 3.5 |
|  | Subependymal hemorrhage without intraventricular diffusion * | 3.30 | 2.09 | 5.01 | ** | 0.00008 | 0.00027 | 3.3 | 2.7 |
|  | Epilepsy to be checked ^15^ | 2.72 | 1.97 | 3.68 | *** | 0.00019 | 0.00051 | 35.5 | 15.9 |
| Dermatology ^3^ | Erythema Intertrigo ^16^ | 10.62 | 8.20 | 13.67 | *** | 0.00009 | 0.00095 | 15.6 | 12.4 |
|  | Atopic dermatitis ^17^ | 8.32 | 7.41 | 9.32 | *** | 0.00052 | 0.00432 | 64.2 | 57.2 |
|  | Miliaria rubra ^18^ | 6.92 | 5.12 | 9.22 | *** | 0.00009 | 0.00066 | 20.9 | 17.9 |
|  | Miliaria ^18^ | 6.89 | 5.63 | 8.38 | *** | 0.00020 | 0.00140 | 13.8 | 13.6 |
|  | Diaper rash ^19^ | 6.04 | 5.70 | 6.40 | *** | 0.00261 | 0.01555 | 11.5 | 11.1 |
|  | Neonatal erythema ^20^ | 4.81 | 2.91 | 7.61 | *** | 0.00005 | 0.00024 | 0.1 | 0.1 |
|  | Impetigo neonatorum ^16^ | 4.46 | 2.95 | 6.54 | *** | 0.00008 | 0.00034 | 0.5 | 0.0 |
|  | Rash ^21^ | 4.37 | 4.26 | 4.48 | *** | 0.01962 | 0.08042 | 36.9 | 23.7 |
|  | Skin furuncle ^22^ | 4.30 | 3.26 | 5.58 | *** | 0.00017 | 0.00072 | 46.7 | 37.4 |
|  | Tinea cruris ^23^ | 4.28 | 2.72 | 6.50 | *** | 0.00007 | 0.00028 | 58.9 | 37.8 |
|  | Dermatitis ^17^ | 4.14 | 4.05 | 4.23 | *** | 0.02895 | 0.10982 | 39.1 | 25.4 |
|  | Pruritus ^24^ | 4.04 | 3.35 | 4.83 | *** | 0.00038 | 0.00152 | 47.3 | 35.3 |
|  | Molluscum Contagiosum ^25^ | 3.91 | 3.48 | 4.39 | *** | 0.00093 | 0.00365 | 37.1 | 28.0 |
|  | Folliculitis ^26^ | 3.86 | 3.37 | 4.40 | *** | 0.00073 | 0.00282 | 44.2 | 18.2 |
|  | Seborrhoeic Dermatitis ^27^ | 3.86 | 3.26 | 4.53 | *** | 0.00048 | 0.00185 | 58.8 | 26.2 |
|  | Tinea Corporis ^23^ | 3.81 | 2.84 | 5.04 | *** | 0.00016 | 0.00062 | 49.1 | 30.0 |
|  | Frostbite * | 3.80 | 2.97 | 4.81 | *** | 0.00023 | 0.00087 | 37.9 | 28.3 |
|  | Granuloma ^28^ | 3.71 | 2.41 | 5.53 | *** | 0.00008 | 0.00031 | 41.6 | 34.0 |
|  | Head furuncle ^22^ | 3.59 | 2.22 | 5.57 | ** | 0.00007 | 0.00025 | 32.3 | 14.3 |
|  | Papular urticaria ^29^ | 3.56 | 3.38 | 3.74 | *** | 0.00539 | 0.01894 | 35.4 | 24.2 |
|  | Tinea pedis ^23^ | 3.52 | 3.13 | 3.95 | *** | 0.00103 | 0.00361 | 53.6 | 39.2 |
|  | Scalp abscess ^26^ | 3.42 | 2.29 | 4.95 | *** | 0.00010 | 0.00035 | 21.7 | 9.3 |
|  | Impetigo ^30^ | 3.40 | 2.99 | 3.85 | *** | 0.00092 | 0.00310 | 33.7 | 18.9 |
|  | Exanthema subitum(roseola infantum) ^31^ | 3.29 | 2.94 | 3.68 | *** | 0.00116 | 0.00382 | 11.3 | 11.3 |
|  | Dyshidrotic eczema ^32^ | 3.29 | 2.49 | 4.29 | *** | 0.00021 | 0.00068 | 67.2 | 49.9 |
|  | Urticaria ^33^ | 3.14 | 3.03 | 3.25 | *** | 0.01273 | 0.03888 | 42.1 | 25.6 |
|  | Acute Urticaria ^33^ | 2.93 | 2.07 | 4.06 | *** | 0.00015 | 0.00045 | 40.7 | 24.4 |
|  | Neoplasm of skin * | 2.85 | 1.83 | 4.28 | * | 0.00010 | 0.00028 | 41.5 | 25.9 |
|  | Paronychia ^22^ | 2.79 | 2.50 | 3.11 | *** | 0.00145 | 0.00403 | 52.4 | 24.9 |
|  | Amelanotic nevus ^34^ | 2.76 | 2.15 | 3.50 | *** | 0.00030 | 0.00083 | 23.7 | 16.2 |
|  | Angioneurotic edema ^35^ | 2.75 | 2.20 | 3.39 | *** | 0.00038 | 0.00105 | 38.9 | 25.2 |
|  | Lichen Planus ^36^ | 2.71 | 2.33 | 3.13 | *** | 0.00082 | 0.00223 | 42.8 | 35.2 |
|  | Dermapostasis ^22^ | 2.69 | 2.26 | 3.17 | *** | 0.00063 | 0.00168 | 45.1 | 27.2 |
|  | Cheilitis ^37^ | 2.67 | 2.19 | 3.22 | *** | 0.00049 | 0.00130 | 76.5 | 56.0 |
|  | Abscess ^38^ | 2.66 | 2.07 | 3.37 | *** | 0.00031 | 0.00083 | 28.3 | 23.1 |
|  | Skin changes^#^ | 2.54 | 2.12 | 3.02 | *** | 0.00060 | 0.00152 | 41.5 | 23.4 |
|  | Edema ^35^ | 2.50 | 1.84 | 3.34 | *** | 0.00022 | 0.00056 | 41.3 | 20.8 |
|  | Onychodysplasia ^39^ | 2.39 | 2.04 | 2.79 | *** | 0.00080 | 0.00192 | 55.5 | 40.3 |
|  | Nail Fungus ^23^ | 2.36 | 2.02 | 2.74 | *** | 0.00086 | 0.00202 | 54.2 | 32.0 |
|  | Tinea manuum ^23^ | 2.35 | 1.80 | 3.02 | *** | 0.00031 | 0.00073 | 70.2 | 55.1 |
|  | Tinea Versicolor ^23^ | 2.30 | 1.85 | 2.83 | *** | 0.00046 | 0.00106 | 18.2 | 4.6 |
|  | White pityriasis ^40^ | 2.30 | 2.12 | 2.48 | *** | 0.00337 | 0.00770 | 60.6 | 35.8 |
|  | Acne ^41^ | 2.20 | 1.78 | 2.69 | *** | 0.00050 | 0.00110 | 83.7 | 17.7 |
|  | Cellulitis ^42^ | 2.09 | 1.88 | 2.31 | *** | 0.00210 | 0.00437 | 44.6 | 25.9 |
|  | Scalp masses ^26^ | 2.06 | 1.75 | 2.41 | *** | 0.00089 | 0.00184 | 25.5 | 14.1 |
|  | Nevus pigmentous ^34^ | 2.06 | 1.72 | 2.45 | *** | 0.00074 | 0.00152 | 55.0 | 39.1 |
| ENT | Eczema of external auditory canal ^#^ | 21.03 | 14.77 | 29.83 | *** | 0.00003 | 0.00066 | 7.6 | 4.3 |
|  | Eczema of external ear ^#^ | 12.47 | 10.63 | 14.58 | *** | 0.00021 | 0.00256 | 6.9 | 4.9 |
|  | Ear examination ^#^ | 5.49 | 4.90 | 6.14 | *** | 0.00076 | 0.00414 | 34.5 | 19.7 |
|  | Nasolacrimal duct obstruction ^#^ | 5.38 | 4.77 | 6.06 | *** | 0.00068 | 0.00364 | 6.3 | 5.3 |
|  | Dacryocystorhinostenosis ^#^ | 4.90 | 3.23 | 7.21 | *** | 0.00007 | 0.00034 | 9.1 | 5.3 |
|  | External otitis ^43^ | 4.27 | 3.94 | 4.62 | *** | 0.00187 | 0.00795 | 48.2 | 21.7 |
|  | External ear Furuncle ^22^ | 4.11 | 3.33 | 5.03 | *** | 0.00029 | 0.00119 | 25.9 | 13.9 |
|  | Cerumen Impaction ^44^ | 3.89 | 3.60 | 4.20 | *** | 0.00220 | 0.00850 | 49.5 | 35.2 |
|  | Otorrhagia ^#^ | 3.06 | 1.99 | 4.53 | ** | 0.00010 | 0.00031 | 39.5 | 24.3 |
|  | Laryngomalacia * | 3.00 | 2.32 | 3.83 | *** | 0.00027 | 0.00080 | 5.1 | 4.6 |
|  | Pharyngoconjunctival fever ^#^ | 2.84 | 1.99 | 3.96 | *** | 0.00015 | 0.00043 | 43.6 | 27.3 |
|  | Congenital laryngeal stridor * | 2.75 | 2.46 | 3.08 | *** | 0.00139 | 0.00381 | 4.3 | 3.7 |
|  | Otitis media ^22^ | 2.46 | 2.38 | 2.55 | *** | 0.01600 | 0.03853 | 52.4 | 34.3 |
|  | Acute laryngopharyngitis ^22^ | 2.33 | 1.99 | 2.71 | *** | 0.00086 | 0.00199 | 42.7 | 25.1 |
|  | Acute rhinitis ^45^ | 2.32 | 2.24 | 2.40 | *** | 0.01730 | 0.03927 | 48.2 | 33.8 |
|  | Contusion of nose ^#^ | 2.30 | 1.72 | 3.02 | *** | 0.00027 | 0.00061 | 44.2 | 33.8 |
|  | Acute pharyngitis ^22^ | 2.28 | 2.21 | 2.35 | *** | 0.02578 | 0.05685 | 44.0 | 28.6 |
|  | Acute laryngitis ^22^ | 2.13 | 2.01 | 2.26 | *** | 0.00667 | 0.01412 | 32.1 | 23.8 |
|  | Acute nasopharyngitis ^22^ | 2.06 | 1.97 | 2.16 | *** | 0.01039 | 0.02119 | 52.8 | 41.6 |
|  | Otalgia ^#^ | 2.03 | 1.69 | 2.42 | *** | 0.00073 | 0.00148 | 61.1 | 53.3 |
| Gastroenterology | Lactose intolerance ^46^ | 4.83 | 3.42 | 6.68 | *** | 0.00010 | 0.00048 | 4.9 | 3.6 |
|  | Gut microbiota dysbiosis ^47^ | 4.17 | 3.46 | 4.99 | *** | 0.00036 | 0.00152 | 35.6 | 20.0 |
|  | Fecal retention ^48^ | 3.58 | 2.41 | 5.16 | *** | 0.00010 | 0.00036 | 3.8 | 2.8 |
|  | Perianorectal abscess ^22^ | 3.46 | 2.25 | 5.14 | *** | 0.00009 | 0.00031 | 8.3 | 2.0 |
|  | Intestinal colic ^49^ | 3.44 | 2.58 | 4.52 | *** | 0.00019 | 0.00064 | 3.7 | 1.6 |
|  | Persistent diarrhea ^7^ | 3.36 | 2.08 | 5.20 | * | 0.00007 | 0.00025 | 17.1 | 7.2 |
|  | Diarrhea ^7^ | 3.34 | 3.19 | 3.49 | *** | 0.00748 | 0.02452 | 22.2 | 16.2 |
|  | Acute enteritis ^50^ | 3.27 | 3.10 | 3.46 | *** | 0.00497 | 0.01607 | 24.8 | 18.6 |
|  | Intestinal malabsorption ^51^ | 3.25 | 2.28 | 4.53 | *** | 0.00013 | 0.00044 | 15.1 | 12.8 |
|  | Dyspepsia ^52^ | 3.23 | 3.16 | 3.30 | *** | 0.03414 | 0.10248 | 22.2 | 14.3 |
|  | Perianal abscess ^53^ | 3.22 | 2.90 | 3.57 | *** | 0.00141 | 0.00455 | 11.1 | 7.3 |
|  | Gastrointestinal dysfunction ^2^ | 3.11 | 2.76 | 3.50 | *** | 0.00111 | 0.00344 | 37.4 | 21.4 |
|  | Spastic colitis ^54^ | 2.79 | 2.25 | 3.42 | *** | 0.00040 | 0.00111 | 27.9 | 12.7 |
|  | Constipation ^55^ | 2.71 | 2.61 | 2.81 | *** | 0.01257 | 0.03334 | 26.7 | 14.4 |
|  | Enteritis ^50^ | 2.54 | 2.48 | 2.61 | *** | 0.03159 | 0.07663 | 20.9 | 15.5 |
|  | Vomiting ^2^ | 2.48 | 2.41 | 2.55 | *** | 0.02392 | 0.05729 | 39.8 | 28.2 |
|  | Chronice diarrhea ^7^ | 2.39 | 1.82 | 3.09 | *** | 0.00029 | 0.00070 | 23.3 | 8.3 |
|  | Change of stool excretion habits ^56^ | 2.34 | 1.93 | 2.82 | *** | 0.00057 | 0.00132 | 29.0 | 16.1 |
|  | Gastroenteritis ^50^ | 2.33 | 2.18 | 2.48 | *** | 0.00463 | 0.01071 | 42.2 | 27.9 |
|  | Bowel dysfunction ^2^ | 2.29 | 1.98 | 2.64 | *** | 0.00100 | 0.00229 | 50.4 | 19.6 |
|  | Hematochezia ^57^ | 2.29 | 2.13 | 2.46 | *** | 0.00396 | 0.00900 | 25.2 | 11.4 |
|  | Anal fissure ^53^ | 2.17 | 2.01 | 2.35 | *** | 0.00359 | 0.00777 | 37.6 | 24.0 |
|  | Anal fistula ^53^ | 2.13 | 1.66 | 2.69 | *** | 0.00039 | 0.00082 | 26.5 | 15.7 |
|  | Gastroesophageal reflux ^58^ | 2.12 | 1.77 | 2.53 | *** | 0.00070 | 0.00149 | 40.0 | 16.4 |
| Infection | Mastitis ^22^ | 6.68 | 3.12 | 13.12 | * | 0.00002 | 0.00012 | 43.8 | 30.3 |
|  | Herpes virus infection ^59^ | 6.43 | 5.96 | 6.93 | *** | 0.00147 | 0.00940 | 33.3 | 20.5 |
|  | Fungal infection ^22^ | 5.47 | 3.64 | 7.98 | *** | 0.00007 | 0.00036 | 75.6 | 36.1 |
|  | Candidiasis ^60^ | 5.35 | 4.93 | 5.80 | *** | 0.00148 | 0.00786 | 19.2 | 13.1 |
|  | Viral exanthem ^22^ | 5.34 | 4.70 | 6.06 | *** | 0.00061 | 0.00322 | 20.1 | 16.0 |
|  | Herpes Simplex ^59^ | 5.22 | 4.45 | 6.09 | *** | 0.00041 | 0.00212 | 45.2 | 22.6 |
|  | Adenovirus enteritis ^22^ | 3.86 | 2.79 | 5.23 | *** | 0.00014 | 0.00052 | 27.0 | 26.3 |
|  | Bacterial infection ^61^ | 3.58 | 2.40 | 5.20 | *** | 0.00010 | 0.00035 | 34.9 | 28.5 |
|  | Virus infection ^22^ | 3.54 | 3.29 | 3.80 | *** | 0.00268 | 0.00942 | 20.5 | 17.6 |
|  | Rotavirus enteritis ^22^ | 3.26 | 2.95 | 3.59 | *** | 0.00155 | 0.00505 | 20.5 | 19.3 |
|  | Infectious diarrhea ^22^ | 3.07 | 2.81 | 3.34 | *** | 0.00211 | 0.00646 | 20.7 | 18.6 |
|  | local infections of skin and subcutaneous tissue ^22^ | 2.72 | 2.13 | 3.42 | *** | 0.00032 | 0.00087 | 39.6 | 22.6 |
|  | Infectious conjunctivitis ^62^ | 2.54 | 1.94 | 3.27 | *** | 0.00028 | 0.00072 | 20.4 | 5.0 |
|  | Herpes pharyngitis ^59^ | 2.48 | 1.49 | 3.93 | *** | 0.00009 | 0.00022 | 35.1 | 21.5 |
|  | Adenovirus infection ^22^ | 2.45 | 2.05 | 2.91 | *** | 0.00064 | 0.00156 | 54.2 | 42.2 |
|  | Herpangina ^22^ | 2.45 | 2.37 | 2.53 | *** | 0.02045 | 0.04864 | 32.3 | 27.9 |
|  | Herpes stomatitis ^22^ | 2.27 | 1.69 | 2.98 | *** | 0.00027 | 0.00061 | 31.3 | 23.1 |
|  | Vesicular stomatitis virosis ^22^ | 2.26 | 1.83 | 2.76 | *** | 0.00050 | 0.00114 | 32.2 | 24.0 |
|  | HFMD ^22^ | 2.25 | 2.14 | 2.37 | *** | 0.00812 | 0.01810 | 33.2 | 26.1 |
|  | Sepsis ^63^ | 2.20 | 1.83 | 2.62 | *** | 0.00067 | 0.00148 | 32.5 | 23.2 |
|  | Cytomegalovirus infection ^22^ | 2.09 | 1.77 | 2.45 | *** | 0.00087 | 0.00182 | 6.5 | 5.4 |
| Musculoskeletal disorders | Coxarthropathy * | 3.78 | 3.60 | 3.97 | *** | 0.00574 | 0.02135 | 7.9 | 6.0 |
|  | Congenital hip dysplasia * | 3.26 | 2.86 | 3.70 | *** | 0.00089 | 0.00291 | 10.3 | 8.2 |
|  | Joint instability * | 3.13 | 3.01 | 3.26 | *** | 0.00980 | 0.03006 | 14.0 | 9.5 |
|  | Subdislocation of the hip * | 3.07 | 2.36 | 3.92 | *** | 0.00025 | 0.00078 | 10.1 | 4.5 |
|  | Dysplastic unilateral hip disease * | 3.01 | 2.41 | 3.72 | *** | 0.00035 | 0.00105 | 10.2 | 7.3 |
|  | Soft Tissue disorders ^64^ | 2.66 | 2.12 | 3.30 | *** | 0.00037 | 0.00099 | 51.5 | 34.3 |
|  | Dislocations, sprains and strains * | 2.64 | 2.17 | 3.19 | *** | 0.00049 | 0.00129 | 31.6 | 23.9 |
|  | Rickets ^65^ | 2.42 | 2.30 | 2.55 | *** | 0.00688 | 0.01651 | 25.2 | 16.3 |
|  | Torticollis * | 2.40 | 2.22 | 2.59 | *** | 0.00338 | 0.00809 | 15.0 | 7.5 |
|  | Dislocation of the hip * | 2.36 | 1.97 | 2.80 | *** | 0.00066 | 0.00155 | 18.0 | 9.6 |
|  | Dislocation of elbow joint * | 2.36 | 1.99 | 2.77 | *** | 0.00073 | 0.00173 | 26.7 | 19.9 |
|  | Developmental Dysplasia of the Hip * | 2.30 | 1.83 | 2.87 | *** | 0.00041 | 0.00094 | 10.3 | 4.9 |
|  | Arthropathy * | 2.16 | 2.08 | 2.24 | *** | 0.01696 | 0.03590 | 40.1 | 19.3 |
|  | Dislocated radial head * | 2.09 | 1.89 | 2.29 | *** | 0.00247 | 0.00514 | 26.7 | 22.5 |
| Nutrition | Hypervitaminosis D ^66^ | 8.35 | 3.83 | 16.77 | ** | 0.00001 | 0.00012 | 24.9 | 15.0 |
|  | Mild malnutrition ^67^ | 4.06 | 2.90 | 5.57 | *** | 0.00012 | 0.00049 | 44.3 | 21.7 |
|  | Iron deficiency ^68^ | 3.72 | 2.37 | 5.62 | *** | 0.00008 | 0.00028 | 29.4 | 13.7 |
|  | Infantile anemia ^69^ | 3.54 | 2.42 | 5.05 | *** | 0.00011 | 0.00038 | 8.7 | 6.6 |
|  | Iron deficiency anemia ^69^ | 3.22 | 2.50 | 4.10 | *** | 0.00025 | 0.00082 | 24.3 | 10.9 |
|  | Vitamin D deficiency ^66^ | 2.95 | 2.76 | 3.16 | *** | 0.00364 | 0.01066 | 40.6 | 21.7 |
|  | Anemia ^69^ | 2.38 | 2.23 | 2.54 | *** | 0.00443 | 0.01049 | 28.2 | 12.3 |
|  | Deficiency of trace elements ^70^ | 2.06 | 1.79 | 2.37 | *** | 0.00112 | 0.00232 | 54.6 | 33.1 |
| Ophthalmology | Palpebral Eczema^#^ | 22.09 | 17.82 | 27.36 | *** | 0.00008 | 0.00174 | 20.2 | 11.1 |
|  | Blepharitis ^71^ | 6.83 | 4.52 | 10.07 | *** | 0.00005 | 0.00036 | 36.4 | 17.9 |
|  | Neonatal Dacryocystitis ^22^ | 5.02 | 4.21 | 5.96 | *** | 0.00034 | 0.00170 | 2.2 | 2.3 |
|  | Dacryocystitis ^22^ | 4.95 | 4.24 | 5.77 | *** | 0.00044 | 0.00216 | 4.3 | 2.8 |
|  | Chronic dacryocystitis ^22^ | 4.62 | 4.27 | 4.98 | *** | 0.00187 | 0.00858 | 4.9 | 4.0 |
|  | Obstruction of lacrimal passage ^#^ | 4.09 | 3.82 | 4.37 | *** | 0.00262 | 0.01061 | 6.2 | 4.8 |
|  | eyelid trichiasis ^#^ | 3.67 | 3.41 | 3.95 | *** | 0.00244 | 0.00889 | 29.4 | 17.4 |
|  | chronic conjunctivitis ^62^ | 3.55 | 3.09 | 4.06 | *** | 0.00074 | 0.00262 | 44.7 | 35.8 |
|  | Phlyctenular conjunctiva ^62^ | 3.38 | 2.32 | 4.79 | *** | 0.00012 | 0.00039 | 40.1 | 23.7 |
|  | Lacrimal duct stenosis ^#^ | 3.30 | 2.96 | 3.68 | *** | 0.00126 | 0.00415 | 15.9 | 10.8 |
|  | Epiphora ^#^ | 3.24 | 2.05 | 4.91 | ** | 0.00008 | 0.00027 | 17.2 | 10.2 |
|  | Conjunctivitis ^62^ | 3.21 | 3.12 | 3.31 | *** | 0.01934 | 0.05955 | 43.8 | 29.6 |
|  | Conjunctival lithiasis ^#^ | 3.05 | 2.02 | 4.45 | ** | 0.00011 | 0.00033 | 46.1 | 37.5 |
|  | Neonatal conjunctivitis ^62^ | 2.95 | 1.95 | 4.31 | ** | 0.00011 | 0.00033 | 0.6 | 0.8 |
|  | Acute conjunctivitis ^62^ | 2.87 | 2.69 | 3.07 | *** | 0.00378 | 0.01080 | 35.1 | 24.6 |
|  | Palpebral edema ^#^ | 2.87 | 2.16 | 3.75 | *** | 0.00023 | 0.00066 | 36.6 | 26.3 |
|  | Conjunctival hemorrhage ^#^ | 2.62 | 2.18 | 3.12 | *** | 0.00058 | 0.00151 | 41.5 | 19.4 |
|  | Hordeolum ^22^ | 2.47 | 2.19 | 2.77 | *** | 0.00138 | 0.00341 | 52.6 | 37.9 |
|  | Trichiasis ^#^ | 2.43 | 1.86 | 3.12 | *** | 0.00030 | 0.00073 | 24.3 | 8.2 |
|  | Eyelid laceration ^#^ | 2.05 | 1.82 | 2.31 | *** | 0.00158 | 0.00325 | 40.3 | 28.6 |
| Respiratory | Acute laryngotracheitis ^72^ | 3.27 | 2.62 | 4.04 | *** | 0.00032 | 0.00106 | 31.3 | 24.4 |
|  | Acute asthmatic bronchitis ^73^ | 3.26 | 2.83 | 3.73 | *** | 0.00079 | 0.00257 | 23.5 | 22.0 |
|  | Upper airway cough syndrome ^74^ | 2.88 | 2.38 | 3.46 | *** | 0.00049 | 0.00140 | 54.6 | 44.6 |
|  | Respiratory disease ^75^ | 2.84 | 2.45 | 3.29 | *** | 0.00077 | 0.00220 | 43.4 | 31.2 |
|  | Upper respiratory disease ^75^ | 2.77 | 2.41 | 3.18 | *** | 0.00089 | 0.00247 | 43.2 | 30.8 |
|  | Acute bronchitis ^73^ | 2.61 | 2.48 | 2.76 | *** | 0.00635 | 0.01642 | 36.1 | 28.0 |
|  | Non-critical bronchial asthma ^76^ | 2.55 | 1.92 | 3.35 | *** | 0.00025 | 0.00063 | 78.7 | 67.8 |
|  | Capillary bronchitis ^73^ | 2.48 | 2.35 | 2.61 | *** | 0.00665 | 0.01631 | 7.1 | 6.7 |
|  | Asthmatic bronchitis ^73^ | 2.40 | 2.33 | 2.46 | *** | 0.03145 | 0.07217 | 25.7 | 24.3 |
|  | Wheeze ^77^ | 2.27 | 1.76 | 2.89 | *** | 0.00035 | 0.00079 | 24.6 | 26.0 |
|  | Flu ^78^ | 2.24 | 2.13 | 2.35 | *** | 0.00853 | 0.01891 | 55.0 | 40.4 |
|  | Acute upper respiratory infection ^79^ | 2.18 | 2.15 | 2.21 | *** | 0.24099 | 0.40927 | 38.4 | 26.6 |
|  | Chronic cough to be investigated ^74^ | 2.07 | 1.94 | 2.21 | *** | 0.00512 | 0.01053 | 48.1 | 35.8 |
|  | Acute tracheitis ^73^ | 2.01 | 1.94 | 2.08 | *** | 0.02070 | 0.04078 | 33.3 | 23.7 |
| Stomatology | Epithelial pearls * | 6.22 | 3.97 | 9.44 | *** | 0.00005 | 0.00031 | 5.0 | 5.3 |
|  | Tooth deposits ^80^ | 6.10 | 4.43 | 8.27 | *** | 0.00009 | 0.00057 | 38.0 | 27.8 |
|  | Angular cheilitis ^22^ | 3.47 | 2.63 | 4.51 | *** | 0.00020 | 0.00070 | 54.3 | 41.1 |
|  | Lip-tie* | 3.38 | 2.72 | 4.16 | *** | 0.00033 | 0.00110 | 33.9 | 27.0 |
|  | Concussion of the teeth ^80^ | 3.21 | 2.41 | 4.21 | *** | 0.00020 | 0.00064 | 53.2 | 37.1 |
|  | Teeth come out late ^80^ | 2.99 | 2.10 | 4.15 | *** | 0.00015 | 0.00044 | 39.3 | 19.9 |
|  | Oral thrush ^60^ | 2.81 | 2.62 | 3.02 | *** | 0.00325 | 0.00909 | 11.4 | 8.6 |
|  | Luxation of teeth ^80^ | 2.73 | 2.06 | 3.56 | *** | 0.00024 | 0.00066 | 54.2 | 39.5 |
|  | Tongue-tie * | 2.69 | 2.57 | 2.82 | *** | 0.00853 | 0.02265 | 16.0 | 7.2 |
|  | Ankyloglossia * | 2.54 | 2.25 | 2.85 | *** | 0.00134 | 0.00339 | 14.9 | 5.2 |
|  | Chronic pulpitis ^22^ | 2.47 | 1.96 | 3.08 | *** | 0.00039 | 0.00095 | 62.7 | 59.3 |
|  | Superficial oral injury ^#^ | 2.41 | 1.67 | 3.40 | * | 0.00016 | 0.00039 | 36.7 | 23.5 |
|  | Stomatitis ^22^ | 2.22 | 1.95 | 2.52 | *** | 0.00131 | 0.00291 | 41.8 | 29.9 |
|  | Ulcerative stomatitis ^22^ | 2.14 | 1.84 | 2.48 | *** | 0.00099 | 0.00212 | 37.8 | 29.9 |
| Umbilical cord | Urachal fistula * | 4.32 | 2.59 | 6.87 | *** | 0.00005 | 0.00023 | 9.8 | 6.3 |
|  | Omphalitis ^22^ | 3.65 | 3.35 | 3.98 | *** | 0.00177 | 0.00644 | 5.8 | 3.3 |
|  | Umbilical Polyp * | 3.30 | 2.85 | 3.81 | *** | 0.00072 | 0.00236 | 4.0 | 2.3 |
|  | Umbilical hernia * | 3.23 | 2.93 | 3.56 | *** | 0.00159 | 0.00511 | 5.9 | 3.8 |
|  | Umbilical haemorrhages of newborn * | 3.08 | 1.97 | 4.62 | * | 0.00009 | 0.00028 | 0.0 | 0.0 |
| Urogenital system | Vulvar Eczema ^#^ | 6.32 | 4.97 | 7.95 | *** | 0.00016 | 0.00099 | 42.5 | 34.6 |
|  | Scrotal eczema ^#^ | 6.02 | 3.70 | 9.43 | *** | 0.00004 | 0.00026 | 32.8 | 33.7 |
|  | Infantile hydrocoele * | 3.28 | 2.20 | 4.74 | *** | 0.00011 | 0.00035 | 9.3 | 3.1 |
|  | Penis disorders ^#^ | 3.16 | 2.02 | 4.75 | ** | 0.00009 | 0.00028 | 58.9 | 39.8 |
|  | Hymen overlong * | 3.00 | 1.92 | 4.50 | * | 0.00009 | 0.00028 | 23.4 | 13.4 |
|  | Labial adhesion ^#^ | 2.21 | 1.94 | 2.51 | *** | 0.00127 | 0.00280 | 15.5 | 12.5 |
|  | Acute vulvitis ^22^ | 2.17 | 1.96 | 2.38 | *** | 0.00230 | 0.00497 | 47.9 | 33.1 |
| Other | Damp obstruction (TCM) * | 8.54 | 3.74 | 17.81 | * | 0.00001 | 0.00011 | 30.5 | 4.1 |
|  | Hypocalcaemia ^81,#^ | 3.30 | 2.95 | 3.69 | *** | 0.00115 | 0.00380 | 34.1 | 20.2 |
|  | Accidental fall * | 3.30 | 2.09 | 5.01 | ** | 0.00008 | 0.00027 | 39.1 | 9.1 |
|  | High-risk newborn * | 3.21 | 2.22 | 4.52 | *** | 0.00013 | 0.00040 | 2.5 | 2.6 |
|  | Pilonidal sinus * | 2.90 | 2.25 | 3.70 | *** | 0.00028 | 0.00080 | 12.0 | 7.5 |
|  | Symptomatic treatment ^#^ | 2.77 | 2.61 | 2.93 | *** | 0.00523 | 0.01433 | 37.2 | 20.7 |
|  | PFO * | 2.69 | 1.78 | 3.92 | * | 0.00012 | 0.00033 | 13.4 | 6.9 |
|  | Subcutaneous mass ^#^ | 2.66 | 2.01 | 3.46 | *** | 0.00025 | 0.00067 | 41.8 | 19.4 |
|  | Granulocytopenia ^#^ | 2.47 | 1.85 | 3.24 | *** | 0.00025 | 0.00062 | 33.8 | 13.6 |
|  | Lymphadenectasis ^22^ | 2.44 | 2.28 | 2.61 | *** | 0.00416 | 0.01007 | 47.1 | 23.3 |
|  | Lower extremity injury * | 2.38 | 1.84 | 3.03 | *** | 0.00033 | 0.00079 | 64.0 | 41.1 |
|  | Jaundice ^82^ | 2.31 | 2.19 | 2.43 | *** | 0.00747 | 0.01707 | 2.7 | 1.4 |
|  | Purpura ^83^ | 2.25 | 1.81 | 2.76 | *** | 0.00048 | 0.00108 | 50.8 | 25.3 |
|  | Accessory auricle * | 2.12 | 1.71 | 2.59 | *** | 0.00053 | 0.00111 | 20.7 | 9.9 |
|  | Fever ^22^ | 2.11 | 2.07 | 2.14 | *** | 0.09323 | 0.17803 | 41.1 | 29.2 |

*** p<0.001 ** p<0.01 * p<0.05

Reference:

1. Abrams EM, Orkin J, Cummings C, Blair B, Chan ES. Dietary exposures and allergy prevention in high-risk infants. *Paediatr Child Health*. 2021;26(8):504-505. doi:10.1093/PCH/PXAB064

2. Cavarelli C, Cavagni G, Deriu FM, et al. Gastrointestinal symptoms in atopic eczema. *Arch Dis Child*. 1998;78(3):230-234. doi:10.1136/ADC.78.3.230

3. Tsakok T, Woolf R, Smith CH, Weidinger S, Flohr C. Atopic dermatitis: the skin barrier and beyond. *Br J Dermatol*. Published online 2019. doi:10.1111/bjd.16934

4. Ravn NH, Ahmadzay ZF, Christensen TA, et al. Bidirectional association between atopic dermatitis, conjunctivitis, and other ocular surface diseases: A systematic review and meta-analysis. *J Am Acad Dermatol*. 2021;85(2):453-461. doi:10.1016/J.JAAD.2020.11.037

5. Pecova R, Frlickova Z, Pec J, Tatar M. Cough sensitivity in atopic dermatitis. *Pulm Pharmacol Ther*. 2003;16(4):203-206. doi:10.1016/S1094-5539(02)00214-6

6. Blum AE, Burgin S. Eczematous Drug Eruptions. *Am J Clin Dermatol*. 2021;22(3):349-366. doi:10.1007/S40257-021-00586-8

7. Cingi C, Demirbas D, Songu M. Allergic rhinitis caused by food allergies. *Eur Arch Otorhinolaryngol*. 2010;267(9):1327-1335. doi:10.1007/S00405-010-1280-5

8. Yokoe T, Tajima T, Yamaguchi N, Morita Y, Chosa E. Surgical Wound Complications after Knee Cruciate Ligament Reconstruction in Patients with Atopic Dermatitis. *J Knee Surg*. 2021;34(11):1237-1242. doi:10.1055/S-0040-1702186

9. Perkin MR, Bahnson HT, Logan K, et al. Factors influencing adherence in a trial of early introduction of allergenic food. *J Allergy Clin Immunol*. 2019;144(6):1595-1605. doi:10.1016/J.JACI.2019.06.046

10. Caubet JC, Szajewska H, Shamir R, Nowak-Węgrzyn A. Non-IgE-mediated gastrointestinal food allergies in children. *Pediatr Allergy Immunol*. Published online 2017. doi:10.1111/pai.12659

11. Han Y, Lee Y, Park H, Park S, Song K. Nutrient intakes of infants with atopic dermatitis and relationship with feeding type. *Nutr Res Pract*. 2015;9(1):57. doi:10.4162/NRP.2015.9.1.57

12. Bawany F, Northcott CA, Beck LA, Pigeon WR. Sleep Disturbances and Atopic Dermatitis: Relationships, Methods for Assessment, and Therapies. *J allergy Clin Immunol Pract*. 2021;9(4):1488-1500. doi:10.1016/J.JAIP.2020.12.007

13. Salloum S, Hatcher V. Rickets and gross motor delay in a child with atopic dermatitis. *Oxford Med Case Reports*. 2021;2021(2):76-79. doi:10.1093/OMCR/OMAA143

14. Torun EG, Ertugrul A, Tekguc DC, Bostanci I. Sleep Patterns and Development of Children with Atopic Dermatitis. *Int Arch Allergy Immunol*. 2020;181(11):871-878. doi:10.1159/000509402

15. Strom MA, Silverberg JI. Allergic disease is associated with childhood seizures: An analysis of the 1997-2013 National Health Interview Survey. *J Allergy Clin Immunol*. 2016;137(3):951. doi:10.1016/J.JACI.2015.09.021

16. Roh YS, Huang AH, Sutaria N, et al. Real-world comorbidities of atopic dermatitis in the US adult ambulatory population. *J Am Acad Dermatol*. Published online November 2021. doi:10.1016/J.JAAD.2021.11.014

17. NOVAK N. Immune mechanisms leading to atopic dermatitis. *J Allergy Clin Immunol*. 2003;112(6):S128-S139. doi:10.1016/j.jaci.2003.09.032

18. Haque MS, Hailu T, Pritchett E, Cusack CA, Allen HB. The Oldest New Finding in Atopic Dermatitis: Subclinical Miliaria as an Origin. *JAMA Dermatology*. 2013;149(4):436-438. doi:10.1001/2013.JAMADERMATOL.109

19. Fölster-Holst R. Differential diagnoses of diaper dermatitis. *Pediatr Dermatol*. 2018;35 Suppl 1:s10-s18. doi:10.1111/PDE.13484

20. Hoeger PH, Harper JI. Neonatal erythroderma: differential diagnosis and management of the “red baby.” *Arch Dis Child*. 1998;79(2):186-191. doi:10.1136/ADC.79.2.186

21. Leung DYM. Atopic dermatitis: More than a rash. *Ann Allergy Asthma Immunol*. 2018;120(6):555-556. doi:10.1016/J.ANAI.2018.03.023

22. Ren Z, Silverberg JI. Association of Atopic Dermatitis With Bacterial, Fungal, Viral, and Sexually Transmitted Skin Infections. *Dermat contact, atopic, Occup drug*. 2020;31(2):157-164. doi:10.1097/DER.0000000000000526

23. Faergemann J. Atopic Dermatitis and Fungi. *Clin Microbiol Rev*. 2002;15(4):545. doi:10.1128/CMR.15.4.545-563.2002

24. Darsow U, Pfab F, Valet M, et al. Pruritus and atopic dermatitis. *Clin Rev Allergy Immunol*. 2011;41(3):237-244. doi:10.1007/S12016-010-8230-2

25. Olsen JR, Piguet V, Gallacher J, Francis NA. Molluscum contagiosum and associations with atopic eczema in children: a retrospective longitudinal study in primary care. *Br J Gen Pract*. 2016;66(642):e53. doi:10.3399/BJGP15X688093

26. Alexander H, Paller AS, Traidl-Hoffmann C, et al. The role of bacterial skin infections in atopic dermatitis: expert statement and review from the International Eczema Council Skin Infection Group. *Br J Dermatol*. 2020;182(6):1331-1342. doi:10.1111/BJD.18643

27. Zander N, Sommer R, Schäfer I, et al. Epidemiology and dermatological comorbidity of seborrhoeic dermatitis: population-based study in 161 269 employees. *Br J Dermatol*. 2019;181(4):743-748. doi:10.1111/BJD.17826

28. Corrà A, Quintarelli L, Verdelli A, Portelli F, Massi D, Caproni M. Granulomatous Dermatitis and Systemic Disease: An Association to Consider. *Biomed Res Int*. 2020;2020. doi:10.1155/2020/3281380

29. Thomas J, Ravi D, Head &. Papular Urticaria: A Known Early Indicator of Atopic Dermatitis. *Acad J Pediatr Neonatol*. 2018;6(3):1-2. doi:10.19080/AJPN.2018.06.555744

30. Loadsman MEN, Verheij TJM, Van Der Velden AW. Impetigo incidence and treatment: A retrospective study of Dutch routine primary care data. *Fam Pract*. Published online 2019. doi:10.1093/fampra/cmy104

31. Ting S, Nixon R. Clinical features of viral exanthems. *Aust J Gen Pract*. 2021;50(4):231-236. doi:10.31128/AJGP-02-20-5246

32. Gladys TE, Maczuga S, Flamm A. Characterizing demographics and cost of care for dyshidrotic eczema. *Contact Dermatitis*. 2022;86(2). doi:10.1111/COD.14007

33. Paller AS, Mina-Osorio P, Vekeman F, et al. Prevalence of type 2 inflammatory diseases in pediatric patients with atopic dermatitis: Real-world evidence. *J Am Acad Dermatol*. Published online 2021. doi:10.1016/J.JAAD.2021.10.038

34. Dellavalle RP, Hester EJ, Stegner DL, et al. Is high mole count a marker of more than melanoma risk? Eczema diagnosis is associated with melanocytic nevi in children. *Arch Dermatol*. 2004;140(5):577-580. doi:10.1001/ARCHDERM.140.5.577

35. Trakaki A, Marsche G. High-Density Lipoprotein (HDL) in Allergy and Skin Diseases: Focus on Immunomodulating Functions. *Biomedicines*. 2020;8(12):1-25. doi:10.3390/BIOMEDICINES8120558

36. Frew JW. We need to talk about Notch: Notch dysregulation as an epiphenomenon in inflammatory skin disease. *Br J Dermatol*. 2019;180(2):431-432. doi:10.1111/BJD.17414

37. Collet E, Jeudy G, Dalac S. Cheilitis, perioral dermatitis and contact allergy. *Eur J Dermatol*. 2013;23(3):303-307. doi:10.1684/EJD.2013.1932

38. Xiang Q, Zhang L, Liu X, et al. Autosomal dominant hyper IgE syndrome from a single centre in Chongqing, China (2009-2018). *Scand J Immunol*. 2020;91(6). doi:10.1111/SJI.12885

39. Chung BY, Choi YW, Kim HO, Park CW. Nail Dystrophy in Patients with Atopic Dermatitis and Its Association with Disease Severity. *Ann Dermatol*. 2019;31(2):121. doi:10.5021/AD.2019.31.2.121

40. Gawai SR, Asokan N, Narayanan B. Association of Pityriasis Alba with Atopic Dermatitis: A Cross-Sectional Study. *Indian J Dermatol*. 2021;66(5):567-568. doi:10.4103/IJD.IJD_936_20

41. Galobardes B, Patel S, Henderson J, Jeffreys M, Smith GD. The association between irregular menstruations and acne with asthma and atopy phenotypes. *Am J Epidemiol*. 2012;176(8):733-737. doi:10.1093/AJE/KWS161

42. Norazirah MN, Khor IS, Adawiyah J, Tamil AM, Azmawati MN. The risk factors of lower limb cellulitis: A case-control study in a tertiary centre. *Malaysian Fam physician Off J Acad Fam Physicians Malaysia*. 2020;15(1):23-29. Accessed February 2, 2022. https://pubmed.ncbi.nlm.nih.gov/32284801/

43. Yariktas M, Yildirim M, Doner F, Baysal V, Dogru H. Allergic contact dermatitis prevalence in patients with eczematous external otitis. *Asian Pacific J allergy Immunol*. 2004;22(1):7-10. Accessed February 3, 2022. https://pubmed.ncbi.nlm.nih.gov/15366652/

44. Michaudet C, Malaty J. Cerumen Impaction: Diagnosis and Management. *Am Fam Physician*. 2018;98(8):525-529. doi:10.12968/PNUR.2013.24.4.191

45. Ren X, Liu Y, Hu L, et al. Associations between home renovation and asthma, allergic rhinitis, and eczema among preschool children in Wuhan, China. *Int J Environ Health Res*. Published online 2021. doi:10.1080/09603123.2021.1955832

46. Grimbacher B, Peters T, Peter HH. Lactose-intolerance may induce severe chronic eczema. *Int Arch Allergy Immunol*. 1997;113(4):516-518. doi:10.1159/000237632

47. Alghamdi HA, Behieldin A, Edris S. Gut microbiome skin axis in the development of atopic dermatitis. *J Pak Med Assoc*. 2021;71(4):1221-1227. doi:10.5455/JPMA.1415

48. Ridha Z, Quinn R, Croaker GDH. Predictors of slow colonic transit in children. *Pediatr Surg Int*. 2015;31(2):137-142. doi:10.1007/S00383-014-3651-2

49. Slattery J, Macfabe DF, Frye RE. The Significance of the Enteric Microbiome on the Development of Childhood Disease: A Review of Prebiotic and Probiotic Therapies in Disorders of Childhood. *Clin Med Insights Pediatr*. 2016;10:CMPed.S38338. doi:10.4137/CMPED.S38338

50. Wang CH, Fu Y, Chi CC. Association of atopic dermatitis with inflammatory bowel disease: A systematic review and meta-analysis. *Dermatologica Sin*. 2020;38(3):159. doi:10.4103/DS.DS_20_20

51. Marks J, Shuster S. Intestinal malabsorption and the skin. *Gut*. 1971;12(11):938-947. doi:10.1136/GUT.12.11.938

52. Jones MP, Walker MM, Ford AC, Talley NJ. The overlap of atopy and functional gastrointestinal disorders among 23,471 patients in primary care. *Aliment Pharmacol Ther*. 2014;40(4):382-391. doi:10.1111/APT.12846

53. Serban ED. Perianal infectious dermatitis: An underdiagnosed, unremitting and stubborn condition. *World J Clin Pediatr*. 2018;7(4):89. doi:10.5409/WJCP.V7.I4.89

54. PUGH SM, RHODES J, MAYBERRY JF, ROBERTS DL, HEATLEY R V., NEWCOMBE RG. Atopic disease in ulcerative colitis and Crohn’s disease. *Clin Allergy*. 1979;9(3):221-223. doi:10.1111/J.1365-2222.1979.TB01545.X

55. Huang YC, Wu MC, Wang YH, Wei JCC. Influence of constipation on atopic dermatitis: A nationwide population-based cohort study in Taiwan. *Int J Clin Pract*. 2021;75(3). doi:10.1111/IJCP.13691

56. Loo EXL, Wang DY, Siah KTH. Association between Irritable Bowel Syndrome and Allergic Diseases: To Make a Case for Aeroallergen. *Int Arch Allergy Immunol*. 2020;181(1):31-42. doi:10.1159/000503629

57. Mennini M, Fiocchi AG, Cafarotti A, et al. Food protein-induced allergic proctocolitis in infants: Literature review and proposal of a management protocol. *World Allergy Organ J*. 2020;13:100471. doi:10.1016/j.waojou.2020.100471

58. Hait EJ, McDonald DR. Impact of Gastroesophageal Reflux Disease on Mucosal Immunity and Atopic Disorders. *Clin Rev Allergy Immunol*. Published online 2019. doi:10.1007/s12016-018-8701-4

59. Novak N, Weighardt H, Valdelvira R, Izquierdo E, Förster I, Cabanillas B. Herpes simplex virus 1 proteins can induce skin inflammation in an atopic dermatitis‐like mouse model. *Exp Dermatol*. 2021;30(11):1699-1704. doi:10.1111/exd.14327

60. Lehman H, Gordon C. The Skin as a Window into Primary Immune Deficiency Diseases: Atopic Dermatitis and Chronic Mucocutaneous Candidiasis. *J Allergy Clin Immunol Pract*. 2019;7(3):788-798. doi:10.1016/j.jaip.2018.11.026

61. Wang X, Shi X-D, Li L-F, Zhou P, Shen Y-W. Classification and possible bacterial infection in outpatients with eczema and dermatitis in China. *Medicine (Baltimore)*. 2017;96(35):e7955. doi:10.1097/MD.0000000000007955

62. Wu KK, Borba AJ, Deng PH, Armstrong AW. Association between atopic dermatitis and conjunctivitis in adults: a population-based study in the United States. *J Dermatolog Treat*. 2021;32(4):455-459. doi:10.1080/09546634.2019.1659480

63. Sobko T, SchiÃ¶tt J, Ehlin A, Lundberg J, Montgomery S, Norman M. Neonatal sepsis, antibiotic therapy and later risk of asthma and allergy. *Paediatr Perinat Epidemiol*. 2010;24(1):88-92. doi:10.1111/j.1365-3016.2009.01080.x

64. Hobbs MR, Grant CC, Thomas MG, et al. Staphylococcus aureus colonisation and its relationship with skin and soft tissue infection in New Zealand children. *Eur J Clin Microbiol Infect Dis*. 2018;37(10):2001-2010. doi:10.1007/s10096-018-3336-1

65. Elsori DH, Hammoud MS. Vitamin D deficiency in mothers, neonates and children. *J Steroid Biochem Mol Biol*. 2018;175:195-199. doi:10.1016/j.jsbmb.2017.01.023

66. Kim M, Kim S-N, Lee Y, Choe Y, Ahn K. Vitamin D Status and Efficacy of Vitamin D Supplementation in Atopic Dermatitis: A Systematic Review and Meta-Analysis. *Nutrients*. 2016;8(12):789. doi:10.3390/nu8120789

67. Thomas L, Naidoo K, Darné S. Zinc deficiency and severe protein-energy malnutrition in a child with atopic eczema. *Clin Exp Dermatol*. 2019;44(2):215-217. doi:10.1111/ced.13634

68. Leung TF, Wang SS, Kwok FYY, Leung LWS, Chow CM, Hon KL. Assessment of dietary food and nutrient intake and bone density in children with eczema. *Hong Kong Med J*. Published online 2017. doi:10.12809/hkmj164684

69. Drury KE, Schaeffer M, Silverberg JI. Association Between Atopic Disease and Anemia in US Children. *JAMA Pediatr*. 2016;170(1):29. doi:10.1001/jamapediatrics.2015.3065

70. Vaughn AR, Foolad N, Maarouf M, Tran KA, Shi VY. Micronutrients in Atopic Dermatitis: A Systematic Review. *J Altern Complement Med*. 2019;25(6):567-577. doi:10.1089/acm.2018.0363

71. Beare JM. Blepharitis and related conditions. *Proc R Soc Med*. 1969;62(1):5-7.

72. Arslan Z, Çipe FE, Özmen S, Kondolot M, Piskin IE, Yöney A. Evaluation of allergic sensitization and gastroesophageal reflux disease in children with recurrent croup. *Pediatr Int*. 2009;51(5):661-665. doi:10.1111/j.1442-200X.2009.02859.x

73. Frankowska J, Kamer B, Trznadel-Budźko E, Rotsztejn H. The retrospective evaluation of pneumonia and bronchitis cases in infants and small children with atopic dermatitis in the practice of a family doctor – personal observations. *Adv Med Sci*. 2010;55(2):250-253. doi:10.2478/v10039-010-0029-0

74. Lewis HM, Haeney M, Jeacock J, Thomas H. Chronic cough in a hospital population; its relationship to atopy and defects in host defence. *Arch Dis Child*. 1989;64(11):1593-1598. doi:10.1136/adc.64.11.1593

75. Belgrave DCM, Simpson A, Buchan IE, Custovic A. Atopic Dermatitis and Respiratory Allergy: What is the Link. *Curr Dermatol Rep*. 2015;4(4):221-227. doi:10.1007/s13671-015-0121-6

76. Golding J, Emmett PM, Rogers IS. Eczema, asthma and allergy. *Early Hum Dev*. 1997;49:S121-S130. doi:10.1016/S0378-3782(97)00058-3

77. Deng X, Yang M, Wang S, et al. Factors Associated With Childhood Asthma and Wheeze in Chinese Preschool-Aged Children. *Front Med*. 2021;8. doi:10.3389/fmed.2021.742581

78. Grunewald SM, Hahn C, Teufel M, et al. Infection with Influenza A Virus Leads to Flu Antigen-Induced Cutaneous Anaphylaxis in Mice. *J Invest Dermatol*. 2002;118(4):645-651. doi:10.1046/j.1523-1747.2002.01732.x

79. Rantala A, Jaakkola JJK, Jaakkola MS. Respiratory Infections in Adults with Atopic Disease and IgE Antibodies to Common Aeroallergens. Zimmer J, ed. *PLoS One*. 2013;8(7):e68582. doi:10.1371/journal.pone.0068582

80. Perugia C, Saraceno R, Ventura A, et al. Atopic dermatitis and dental manifestations. *Ital J Dermatology Venereol*. 2017;152(2). doi:10.23736/S0392-0488.16.05224-X

81. Bove-Fenderson E, Mannstadt M. Hypocalcemic disorders. *Best Pract Res Clin Endocrinol Metab*. 2018;32(5):639-656. doi:10.1016/j.beem.2018.05.006

82. Wei C-C, Lin C-L, Shen T-C, Kao C-H. Neonatal jaundice and risks of childhood allergic diseases: a population-based cohort study. *Pediatr Res*. 2015;78(2):223-230. doi:10.1038/pr.2015.89

83. Bahoush G, Poorasgari A, Nojomi M. Relationship of primary immune thrombocytopenic purpura and atopia among children: a case control study. *Sci Rep*. 2020;10(1):11717. doi:10.1038/s41598-020-68647-2

**Table S2 The background pediatric diseases distribution in this study.** (Only 1269 diseases with visiting counts more than 100; The original Chinese diagnosis terms were used in this table and the ICD-10 code will help to lookup the corresponding terms in English)

| ICD-10 | Diseases | visiting counts |
| --- | --- | --- |
| J06.900 | 急性上呼吸道感染 | 1412966 |
| J40.03 | 支气管炎 | 576458 |
| R50.901 | 发热待查 | 484794 |
| J18.900 | 肺炎 | 218173 |
| J45.901 | 哮喘性支气管炎 | 150339 |
| J03.900 | 急性扁桃体炎 | 142919 |
| K30.x00 | 消化不良 | 136372 |
| K52.904 | 肠炎 | 128980 |
| R10.401 | 腹痛待查 | 126239 |
| R11.x00a | 呕吐待查 | 122005 |
| L30.902 | 湿疹 | 115359 |
| J06.900x003 | 上呼吸道感染 | 107408 |
| J02.904 | 急性咽炎 | 103532 |
| L30.900 | 皮炎 | 96441 |
| B08.501 | 疱疹性咽峡炎 | 89715 |
| J30.400 | 过敏性鼻炎 | 80628 |
| H66.905 | 中耳炎 | 78969 |
| T14.200 | 骨折 | 78212 |
| Z71.900 | 咨询 | 77266 |
| J45.901 | 哮喘 | 76010 |
| J04.100 | 急性气管炎 | 75647 |
| N47.x00x001 | 包茎 | 75208 |
| R21.x02 | 皮疹 | 74664 |
| M19.991 | 关节病 | 72544 |
| S09.905 | 头部外伤 | 65466 |
| H10.900 | 结膜炎 | 63409 |
| H52.301 | 屈光不正 | 59922 |
| J00.x00 | 急性鼻炎 | 57253 |
| G40.901 | 癫痫 | 56798 |
| D18.000x001 | 血管瘤 | 54559 |
| E30.101 | 性早熟 | 51317 |
| J11.101 | 流行性感冒 | 48114 |
| R63.000 | 食欲不振 | 47374 |
| F95.900 | 抽动症 | 47059 |
| J32.900 | 鼻窦炎 | 46096 |
| L50.901 | 荨麻疹 | 45835 |
| K59.001 | 便秘 | 43071 |
| K02.901 | 龋齿 | 42799 |
| T14.900 | 损伤 | 42195 |
| R50.900 | 发热 | 42131 |
| R62.800a | 生长迟缓 | 41096 |
| R05.x00 | 咳嗽 | 40524 |
| K40.901 | 腹股沟斜疝 | 39455 |
| Z00.001 | 健康查体 | 39010 |
| E03.100 | 先天性甲状腺功能减退症 | 37906 |
| N47.x01 | 包皮过长 | 37680 |
| K29.700 | 胃炎 | 37461 |
| M25.301 | 关节不稳定 | 36848 |
| R04.000 | 鼻出血 | 36714 |
| J00.x00 | 急性鼻咽炎 | 36137 |
| E25.001 | 注意缺陷多动障碍 | 35734 |
| K52.904 | 腹泻 | 33870 |
| Q38.100 | 舌系带过短 | 33401 |
| R10.402 | 腹痛 | 32766 |
| J35.201 | 腺样体肥大 | 32228 |
| J18.008 | 支气管肺炎 | 31865 |
| I51.904 | 心脏病 | 31539 |
| Z01.001 | 眼检查 | 30956 |
| B08.401 | 手足口病 | 29786 |
| R11.x03 | 呕吐 | 29688 |
| F89.x00 | 发育障碍 | 29654 |
| T30.000 | 烧(烫)伤 | 29386 |
| P07.300 | 早产儿 | 29088 |
| Z01.200 | 牙科检查 | 28926 |
| Z00.301 | 青春期女孩 | 28157 |
| Z00.000 | 一般性医学检查 | 27123 |
| J04.000 | 急性喉炎 | 26791 |
| J20.904 | 急性支气管炎 | 26581 |
| R17.x00 | 黄疸 | 25309 |
| R56.803b | 抽搐 | 25253 |
| J03.901 | 急性化脓性扁桃体炎 | 25244 |
| N43.301 | 鞘膜积液 | 24628 |
| J40.03 | 毛细支气管炎 | 24255 |
| A09.905 | 急性肠炎 | 23847 |
| E34.300 | 身材矮小 | 23051 |
| R06.501 | 鼾症 | 22823 |
| E55.001 | 佝偻病 | 22410 |
| R05.x00 | 慢性咳嗽 | 22238 |
| R05.x00 | 慢性咳嗽待查 | 21416 |
| M16.900 | 髋关节病 | 20220 |
| F80.900 | 语言发育落后 | 19932 |
| A09.901 | 胃肠炎 | 19859 |
| N39.001 | 泌尿道感染 | 19612 |
| E60.x00x001 | 锌缺乏 | 19307 |
| M25.591 | 关节痛 | 18447 |
| R32.02 | 遗尿 | 18056 |
| H10.100 | 过敏性结膜炎 | 17714 |
| Z51.901 | 对症治疗 | 17467 |
| Q55.606 | 隐匿性阴茎 | 17386 |
| L50.901 | 丘疹性荨麻疹 | 16634 |
| I88.906 | 淋巴结炎 | 16606 |
| J31.000 | 慢性鼻炎 | 16315 |
| K92.200x00 | 便血 | 16299 |
| N48.101 | 龟头包皮炎 | 15814 |
| D64.903 | 贫血 | 15524 |
| R51.x00 | 头痛待查 | 14946 |
| J45.005 | 咳嗽变异性哮喘 | 14936 |
| Q24.901 | 先天性心脏病 | 14826 |
| R35.x00x001 | 尿频 | 14575 |
| F90.900 | 多动性障碍 | 14457 |
| J01.900 | 急性鼻窦炎 | 14251 |
| D69.004 | 过敏性紫癜 | 14190 |
| A41.901 | 败血症 | 14095 |
| N76.000 | 外阴阴道炎 | 14009 |
| T14.001 | 皮肤挫伤 | 13939 |
| K56.101 | 肠套叠 | 13069 |
| R56.803 | 抽搐待查 | 12695 |
| R59.900 | 淋巴结增大 | 12681 |
| R31.x00 | 血尿 | 12669 |
| M43.600 | 斜颈 | 12390 |
| H02.901 | 眼睑肿物 | 12300 |
| N47.x03 | 包皮粘连 | 12235 |
| J32.900 | 慢性鼻窦炎 | 11957 |
| M65.909 | 滑膜炎 | 11815 |
| E66.900 | 肥胖症 | 11701 |
| T17.202 | 咽异物 | 11563 |
| Z01.102 | 听力检查 | 11547 |
| B37.001 | 鹅口疮 | 11282 |
| G47.900 | 睡眠障碍 | 11203 |
| H10.301 | 急性结膜炎 | 11103 |
| L30.500 | 白色糠疹 | 11020 |
| G70.903 | 体检 | 10989 |
| K60.201 | 肛裂 | 10675 |
| K04.500 | 慢性根尖周炎 | 10649 |
| P59.902 | 新生儿黄疸 | 10642 |
| N13.300 | 肾积水 | 10463 |
| R51.x00 | 头痛 | 10394 |
| R01.100 | 心脏杂音 | 10339 |
| E46.x00x00 | 营养不良 | 10314 |
| R94.501 | 肝功能异常 | 9913 |
| M30.300 | 急性发热性粘膜皮肤淋巴结综合征(川崎病) | 9669 |
| H04.509 | 泪道阻塞 | 9532 |
| J04.102 | 气管炎 | 9473 |
| A04.903 | 感染性腹泻 | 9451 |
| E05.806 | 高TSH血症 | 9394 |
| G93.407 | 脑病 | 9342 |
| K40.900 | 腹股沟疝 | 9311 |
| L22.02 | 尿布疹 | 9297 |
| T14.101 | 皮肤裂伤 | 9269 |
| R22.901 | 局部肿物 | 9237 |
| R42.x00x004a | 头晕待查 | 8987 |
| R52.900 | 疼痛 | 8843 |
| S01.506 | 唇裂伤 | 8801 |
| Z00.302 | 青春期男孩 | 8738 |
| M65.992 | 腱鞘炎 | 8731 |
| Q53.902 | 隐睾 | 8655 |
| K00.708 | 乳牙滞留 | 8622 |
| L03.901 | 蜂窝织炎 | 8370 |
| R14.x00x00 | 腹胀 | 8369 |
| Q21.100 | 房间隔缺损 | 8330 |
| J02.909 | 咽炎 | 8308 |
| B34.901 | 病毒感染 | 8230 |
| G04.916 | 脑炎 | 7960 |
| A08.000 | 轮状病毒肠炎 | 7816 |
| K61.001 | 肛周脓肿 | 7763 |
| F90.902 | 运动发育迟缓 | 7751 |
| Q21.000 | 室间隔缺损 | 7659 |
| E88.903 | 遗传代谢性疾病 | 7511 |
| L90.501 | 瘢痕 | 7475 |
| E30.103 | 青春期快速进展 | 7407 |
| S53.001 | 桡骨头脱位 | 7371 |
| R56.803 | 惊厥 | 7354 |
| J31.000x001 | 鼻炎 | 7310 |
| T18.900 | 消化道异物 | 7280 |
| J03.900x002 | 扁桃体炎 | 7145 |
| H61.200 | 耵聍栓塞 | 7134 |
| I49.900 | 心律失常 | 7052 |
| N76.200 | 急性外阴炎 | 6992 |
| H02.000 | 睑内翻和倒睫 | 6979 |
| R07.401 | 胸痛 | 6759 |
| E55.900 | 维生素D减少症 | 6720 |
| K12.106 | 口炎 | 6650 |
| A49.102 | 链球菌感染 | 6618 |
| T17.101 | 鼻腔异物 | 6446 |
| B00.203 | 疱疹性咽炎 | 6416 |
| J98.800x002a | 反复呼吸道感染 | 6369 |
| B00.201 | 疱疹龈口炎 | 6357 |
| L08.913 | 脐炎 | 6312 |
| E34.301 | 矮小症 | 6134 |
| H04.401 | 慢性泪囊炎 | 6115 |
| W44.900 | 异物进入或穿入眼或自然腔口 | 6015 |
| S01.101 | 眼睑裂伤 | 6014 |
| N76.301 | 慢性外阴炎 | 5955 |
| R47.101 | 构音不全 | 5846 |
| H60.901 | 外耳道炎 | 5832 |
| P59.901 | 新生儿高胆红素血症 | 5713 |
| R05.x00x001a | 百日咳综合症 | 5706 |
| K37.01 | 阑尾炎 | 5689 |
| E55.900 | 维生素D缺乏症 | 5615 |
| R68.101 | 婴儿过度哭闹 | 5585 |
| E41.x00 | 营养性消瘦 | 5326 |
| B07.x04 | 疣 | 5316 |
| N04.900 | 肾病综合征 | 5306 |
| J31.203 | 慢性咽炎 | 5262 |
| X50.901 | 扭伤 | 5112 |
| D69.602 | 血小板减少症 | 5033 |
| F80.900 | 语言发育障碍 | 4979 |
| F95.903 | 多发性抽动症 | 4975 |
| H00.101 | 睑板腺囊肿 | 4932 |
| B00.900 | 疱疹病毒感染 | 4923 |
| B00.901 | EB病毒感染 | 4902 |
| R49.001 | 声嘶 | 4901 |
| J35.101 | 扁桃体肥大 | 4867 |
| L03.001 | 甲沟炎 | 4856 |
| M87200/0 | 痣 | 4788 |
| B37.900 | 念珠菌病 | 4774 |
| K04.005 | 牙髓炎 | 4751 |
| K42.902 | 脐疝 | 4751 |
| K29.100 | 急性胃炎 | 4723 |
| B25.900 | 巨细胞病毒感染 | 4680 |
| Q69.900 | 多指[趾]畸形 | 4402 |
| M84.191 | 骨折,原因未特指者 | 4385 |
| H00.001 | 睑腺炎 | 4349 |
| K12.112 | 口腔炎 | 4297 |
| R62.801 | 生长迟滞 | 4266 |
| Q38.101 | 舌系带短缩 | 4216 |
| M95.401 | 漏斗胸 | 4212 |
| G80.901 | 脑性瘫痪 | 4192 |
| J31.101 | 慢性鼻咽炎 | 4187 |
| P28.801 | 先天性喉喘鸣 | 4186 |
| Q18.102 | 先天性耳前瘘管 | 4076 |
| K92.901 | 胃肠功能紊乱 | 4068 |
| E25.004 | 先天性肾上腺皮质增生症 | 4051 |
| L65.904 | 脱发 | 4046 |
| R07.400a | 胸闷待查 | 4041 |
| S09.901 | 鼻损伤 | 4009 |
| H04.500x004 | 泪道狭窄 | 3980 |
| E23.009 | 生长激素缺乏症 | 3908 |
| N90.808 | 阴唇粘连 | 3887 |
| H10.101 | 变应性结膜炎 | 3874 |
| F80.000 | 特定性言语构音障碍 | 3856 |
| B08.100 | 传染性软疣 | 3828 |
| Q65.801 | 先天性髋关节发育不良 | 3785 |
| Q54.900 | 尿道下裂 | 3777 |
| R22.904 | 皮下结节 | 3776 |
| E83.503 | 低钙血症 | 3753 |
| R63.001 | 厌食 | 3732 |
| S42.000 | 锁骨骨折 | 3716 |
| K12.107 | 溃疡性口炎 | 3702 |
| K07.400 | 安氏III类错合 | 3673 |
| K11.201 | 急性腮腺炎 | 3664 |
| J98.900 | 呼吸道疾病 | 3642 |
| L29.200 | 外阴瘙痒症 | 3597 |
| J35.300 | 扁桃体腺样体肥大 | 3486 |
| J21.901 | 急性喘息性支气管炎 | 3447 |
| N48.102 | 龟头炎 | 3445 |
| Y32.900 | 机动车辆碰撞，意图不确定 | 3431 |
| B35.300 | 脚癣 | 3408 |
| E61.700 | 微量元素缺乏 | 3370 |
| S00.002 | 头皮血肿 | 3352 |
| K29.201 | 幽门螺旋杆菌相关胃炎 | 3316 |
| B34.000 | 腺病毒感染 | 3245 |
| D55.000 | 葡萄糖-6-磷酸脱氢酶缺乏 | 3244 |
| K59.901 | 肠功能紊乱 | 3229 |
| A41.901 | 脓毒症 | 3170 |
| N28.901 | 肾病 | 3162 |
| S30.901 | 阴囊急诊 | 3145 |
| R62.000 | 发育指标延迟 | 3113 |
| R61.901 | 盗汗 | 3106 |
| M79.600 | 肢痛 | 3105 |
| M60.202 | 异物反应 | 3093 |
| B27.900 | 传染性单核细胞增多症 | 3024 |
| R94.600 | 甲状腺功能异常 | 3024 |
| M41.900 | 脊柱侧弯 | 3018 |
| J06.000 | 急性咽喉炎 | 3014 |
| S09.904 | 耳损伤 | 2987 |
| H92.002 | 耳痛 | 2986 |
| H50.005 | 内斜视 | 2985 |
| R80.01 | 蛋白尿 | 2973 |
| R07.000 | 咽痛 | 2967 |
| R55.04 | 晕厥 | 2965 |
| N63.x00 | 乳房肿块 | 2952 |
| R22.002 | 头皮肿物 | 2905 |
| K12.109 | 口腔粘膜溃疡 | 2901 |
| J39.900 | 上呼吸道疾病 | 2900 |
| B35.101 | 甲癣 | 2868 |
| T17.900 | 呼吸道异物 | 2864 |
| R63.300x003a | 喂养不当 | 2825 |
| M67.401 | 腱鞘囊肿 | 2813 |
| E64.300 | 佝偻病后遗症 | 2807 |
| Q43.004 | 脐茸 | 2800 |
| L01.001 | 脓疱病 | 2761 |
| T78.101 | 牛奶蛋白过敏 | 2756 |
| D18.100 | 淋巴管瘤 | 2732 |
| R42.x00x00 | 头晕 | 2727 |
| D69.005+ | 过敏性紫癜肾炎 | 2725 |
| H04.503 | 鼻泪管阻塞 | 2710 |
| R32.01 | 尿失禁 | 2708 |
| F84.000 | 孤独症谱系障碍 | 2696 |
| P23.901 | 新生儿肺炎 | 2696 |
| Q43.901 | 先天性肛门畸形 | 2686 |
| S13.101 | 颈椎半脱位 | 2682 |
| L60.300 | 甲营养不良 | 2633 |
| L23.901 | 过敏性皮炎 | 2625 |
| F93.900 | 情绪障碍 | 2598 |
| R11.x02 | 恶心 | 2583 |
| Q66.001 | 先天性马蹄内翻足 | 2520 |
| E03.101 | 先天性甲状腺机能不全 | 2516 |
| D48.902 | 色素痣 | 2511 |
| B01.901 | 水痘 | 2510 |
| L02.902 | 皮肤脓肿 | 2507 |
| S60.000 | 手指挫伤 | 2503 |
| F90.100 | 多动性品行障碍 | 2486 |
| Z01.101 | 耳检查 | 2485 |
| H50.104 | 外斜视 | 2483 |
| R62.801 | 发育迟缓 | 2479 |
| R47.801 | 言语不清 | 2478 |
| R21.02 | 病毒性皮疹 | 2455 |
| B08.200 | 幼儿急疹(婴儿玫瑰疹) | 2434 |
| R21.02 | 皮疹待查 | 2418 |
| L28.004 | 苔癣 | 2411 |
| E03.900 | 甲状腺机能减退症 | 2383 |
| S73.002 | 髋关节脱位 | 2380 |
| J30.400 | 变应性鼻炎 | 2378 |
| R17.01 | 黄疸待查 | 2358 |
| R04.001 | 鼻出血(鼻衄) | 2339 |
| K56.700 | 肠梗阻 | 2332 |
| K21.900x00 | 胃食管反流 | 2317 |
| J30.401 | 过敏性鼻炎(变应性鼻炎) | 2278 |
| J35.000 | 慢性扁桃体炎 | 2270 |
| J45.004 | 过敏性鼻炎伴哮喘 | 2262 |
| L73.901 | 毛囊炎 | 2253 |
| K72.905 | 肝功能不全 | 2243 |
| J03.904 | 急性化脓性扁桃腺炎 | 2223 |
| R19.600 | 口臭 | 2220 |
| Q67.700 | 鸡胸 | 2187 |
| H10.402 | 慢性结膜炎 | 2160 |
| S53.101 | 肘关节脱位 | 2153 |
| J45.900 | 哮喘伴感染 | 2146 |
| N92.600 | 月经不规则 | 2141 |
| Q17.003 | 副耳 | 2131 |
| S69.903 | 手指损伤 | 2125 |
| K21.900 | 胃食管返流病 | 2115 |
| T56.001 | 铅中毒 | 2075 |
| L20.901 | 特应性皮炎 | 2068 |
| E05.900 | 甲状腺机能亢进症 | 2032 |
| S42.301 | 肱骨骨折 | 2009 |
| H66.900x002 | 急性中耳炎 | 2001 |
| Z00.300x001a | 生长痛 | 1991 |
| R06.503 | 磨牙症 | 1987 |
| K04.002 | 慢性牙髓炎 | 1977 |
| H02.400 | 上睑下垂 | 1971 |
| Q04.303 | 脑发育落后 | 1954 |
| F81.900 | 学习技能发育障碍 | 1946 |
| F98.501 | 口吃 | 1939 |
| G51.002 | 面神经麻痹 | 1938 |
| K12.103 | 口腔感染 | 1927 |
| Q04.303b | 脑发育不良 | 1923 |
| P36.901 | 新生儿败血症 | 1917 |
| G93.001 | 蛛网膜囊肿 | 1880 |
| R56.001 | 热性惊厥 | 1878 |
| R23.801 | 皮肤改变 | 1869 |
| R19.400 | 大便习惯改变 | 1866 |
| K92.000 | 呕血 | 1824 |
| E30.102 | 真性性早熟 | 1814 |
| M13.991 | 关节炎 | 1802 |
| S93.401 | 踝关节扭伤 | 1790 |
| J37.002 | 慢性喉炎 | 1776 |
| Q35.900 | 腭裂 | 1767 |
| G91.900 | 脑积水 | 1759 |
| H65.000x002a | 亚急性分泌性中耳炎 | 1758 |
| G93.002a | 室管膜下囊肿 | 1754 |
| Q25.000 | 动脉导管未闭 | 1717 |
| A93.801 | 疱疹性口炎病毒病 | 1713 |
| S02.500 | 牙折断 | 1711 |
| I50.906 | 心肌损害 | 1710 |
| L63.900 | 斑秃 | 1693 |
| R60.100 | 浮肿 | 1693 |
| R05.x00 | 上气道咳嗽综合征 | 1681 |
| L72.902 | 皮样囊肿 | 1672 |
| F98.200 | 婴儿和儿童期喂养障碍 | 1667 |
| G70.002 | 重症肌无力，眼肌型 | 1665 |
| R15.01 | 大便失禁 | 1660 |
| r42 02 | 哭吵待查 | 1654 |
| H11.301 | 结膜出血 | 1651 |
| N76.202 | 外阴炎 | 1644 |
| J04.200 | 急性喉气管炎 | 1618 |
| H91.900 | 耳聋 | 1617 |
| G51.800 | 面神经炎 | 1593 |
| H90.500 | 感音神经性耳聋 | 1587 |
| K52.905 | 急性胃肠炎 | 1579 |
| F79.900 | 精神发育迟缓 | 1575 |
| L70.901 | 痤疮 | 1572 |
| R58.x00x006 | 出血 | 1560 |
| L51.202 | 多处烫伤(烧伤) | 1556 |
| R22.101 | 颈部局部肿物 | 1548 |
| R63.500b | 超重 | 1537 |
| Q44.200 | 先天性胆道闭锁 | 1533 |
| Z13.900 | 特殊筛查 | 1521 |
| L21.900 | 脂溢性皮炎 | 1518 |
| D69.204 | 紫癜 | 1515 |
| I51.400 | 心肌炎 | 1512 |
| H93.200 | 听力减退 | 1506 |
| J02.904 | 咽峡炎 | 1502 |
| F84.001 | 自闭症 | 1497 |
| D69.406 | 血小板减少性紫癜 | 1495 |
| T14.300 | 脱位、扭伤和劳损 | 1489 |
| K13.013 | 唇炎 | 1488 |
| B36.000 | 花斑癣 | 1479 |
| F84.000 | 童年孤独症 | 1476 |
| T17.200 | 咽部异物 | 1475 |
| E60 .01 | 锌缺乏[症] | 1469 |
| L67.101 | 白发 | 1463 |
| Q17.302 | 先天性耳廓畸形 | 1461 |
| B00.902 | 单纯疱疹 | 1434 |
| R00.000 | 心动过速 | 1420 |
| K04.401 | 急性根尖周炎 | 1418 |
| S01.001 | 头皮裂伤 | 1402 |
| R06.000a | 气促 | 1398 |
| T88.701 | 药物反应 | 1394 |
| H04.300x004 | 泪囊炎 | 1387 |
| B07.03 | 寻常疣 | 1378 |
| P38.01 | 新生儿脐炎 | 1369 |
| R94.500a | 肝功能损害 | 1366 |
| Q55.601 | 隐匿性阴茎(先天性) | 1365 |
| K60.300 | 肛瘘 | 1347 |
| K59.300 | 巨结肠 | 1340 |
| G24.902 | 运动障碍 | 1339 |
| L72.105 | 皮脂腺囊肿 | 1334 |
| H65.301 | 慢性分泌性中耳炎 | 1326 |
| R19.001 | 腹部肿物 | 1317 |
| T13.900 | 下肢损伤 | 1308 |
| Q89.202 | 甲状舌管囊肿 | 1306 |
| K52.904 | 腹泻病 | 1293 |
| K58.902 | 肠痉挛 | 1293 |
| F95.801 | 眨眼症 | 1286 |
| M79.900 | 软组织疾患 | 1286 |
| K04.501 | 根尖牙周炎 | 1280 |
| K59.101 | 肠道菌群失调 | 1279 |
| R22.903 | 皮肤肿物 | 1260 |
| V89.900 | 车辆事故中人员损伤 | 1257 |
| J11.000 | 流行性感冒伴肺炎 | 1250 |
| E22.802 | 中枢性性早熟 | 1235 |
| R47.101 | 构音障碍 | 1220 |
| Q21.001 | 先天性室间隔缺损 | 1219 |
| T78.300 | 血管神经性水肿 | 1216 |
| K29.502 | 慢性胃炎 | 1212 |
| B00.204 | 疱疹性龈口炎 | 1210 |
| J18.000a | 急性支气管肺炎 | 1209 |
| Q65.803 | 髋关节发育不良 | 1207 |
| R35 .01 | 多尿 | 1203 |
| H00.002 | 眼睑脓肿 | 1196 |
| M16.301 | 发育异常性单侧髋关节病 | 1193 |
| T11.900 | 上肢损伤 | 1188 |
| R23.301 | 皮肤出血 | 1186 |
| M53.203 | 颈椎不稳定 | 1184 |
| Q55.601 | 蹼状阴茎 | 1184 |
| H65.900x001 | 分泌性中耳炎 | 1180 |
| K52.912 | 慢性腹泻 | 1179 |
| F79.901 | 智力低下 | 1166 |
| K52.203 | 过敏性肠炎 | 1157 |
| Q70.400 | 多指[趾]和并指[趾]畸形 | 1157 |
| L29.900x001 | 瘙痒症 | 1154 |
| J00.01 | 鼻咽炎 | 1145 |
| R23.000 | 发绀 | 1129 |
| G06.006 | 颅内感染 | 1127 |
| N20.000 | 肾结石 | 1119 |
| H60.001 | 外耳疖 | 1115 |
| L08.900 | 皮肤和皮下组织局部感染 | 1110 |
| J44.001 | 喘息型支气管炎 | 1102 |
| B08.200 | 幼儿急疹(第六病婴儿玫瑰疹) | 1099 |
| R47.100 | 构音困难 | 1098 |
| G81.903 | 偏瘫 | 1066 |
| R06.200 | 喘息 | 1064 |
| Q38.001 | 唇系带短缩 | 1061 |
| M95.405 | 胸壁畸形 | 1051 |
| E83.001 | 肝豆状核变性 | 1036 |
| E70.000 | 苯丙酮尿症 | 1033 |
| S52.801 | 桡骨骨折 | 1032 |
| S03.200 | 牙脱位 | 1026 |
| A49.809 | 幽门螺旋杆菌感染 | 1023 |
| M54.502 | 腰痛 | 1023 |
| L08.801 | 皮肤感染性窦道 | 1021 |
| R63.500 | 生长过快 | 1021 |
| H10.201 | 卡他性结膜炎 | 1020 |
| N13.501 | 肾盂输尿管连接处狭窄 | 1020 |
| H16.901 | 角膜炎 | 1019 |
| P39.101 | 新生儿泪囊炎 | 1019 |
| K11.203 | 化脓性腮腺炎 | 1007 |
| L08.907 | 皮肤感染 | 1007 |
| J45.903 | 支气管哮喘，非危重 | 1004 |
| R59.001 | 颈淋巴结肿大 | 1003 |
| D70.x01 | 粒细胞减少 | 999 |
| E66.900x001 | 单纯性肥胖 | 999 |
| M93.901 | 骨骺炎 | 999 |
| S00.501 | 舌裂伤 | 994 |
| F98.902 | 行为问题(障碍) | 985 |
| R06.001 | 呼吸困难 | 982 |
| H66.001 | 急性化脓性中耳炎 | 976 |
| L05.900 | 潜毛窦 | 969 |
| H53.001 | 弱视 | 968 |
| J04.002 | 喉炎 | 968 |
| M48.900 | 脊椎病 | 965 |
| L08.002 | 脓疱疹 | 963 |
| K40.314 | 嵌顿性腹股沟斜疝 | 954 |
| M79.604 | 足痛 | 954 |
| N39.900 | 泌尿系统疾病 | 953 |
| B35.200 | 手癣 | 950 |
| N02.801 | IgA肾病 | 945 |
| S72.900 | 股骨骨折 | 945 |
| B00.203 | 疱疹病毒性口炎 | 941 |
| S82.202 | 胫骨骨折 | 939 |
| K35.101 | 阑尾脓肿 | 935 |
| K83.502 | 胆总管囊肿 | 935 |
| M21.061 | 后天性膝外翻 | 935 |
| R90.000 | 颅内占位性病变 | 935 |
| S02.900 | 颅骨骨折 | 931 |
| K92.210 | 消化道出血 | 922 |
| S39.903 | 会阴损伤 | 920 |
| F80.301 | 运动发育落后 | 912 |
| Q21.102 | 先天性房间隔缺损 | 911 |
| R20.801 | 肢体麻木 | 911 |
| S00.001 | 头皮挫伤 | 911 |
| T16.x00 | 耳内异物 | 911 |
| J35.201 | 儿童多动综合症 | 901 |
| Q04.902 | 先天性脑发育异常 | 899 |
| S42.401 | 肱骨髁上骨折 | 898 |
| R31.01 | 血尿待查 | 896 |
| K04.700 | 根尖周脓肿 | 894 |
| F98.800 | 情感性擦腿综合征 | 893 |
| I88.000 | 肠系膜淋巴结炎 | 887 |
| M87300/0 | 无色素痣 | 879 |
| S00.100 | 眼睑挫伤 | 879 |
| Q55.602 | 小阴茎 | 874 |
| K00.100 | 多生牙 | 871 |
| D50.902 | 缺铁性贫血 | 867 |
| R59.901 | 淋巴结肿大 | 863 |
| T17.500 | 支气管内异物 | 863 |
| R62.801 | 生长发育迟缓 | 862 |
| F95.800 | 多发性抽动 | 857 |
| H02.004 | 倒睫 | 847 |
| F98.802 | 咬指甲 | 844 |
| M53.200 | 脊柱不稳定性疾患 | 841 |
| K13.006 | 唇囊肿 | 836 |
| Z04.100 | 交通事故后接受检查和观察 | 834 |
| I86.101 | 精索静脉曲张 | 827 |
| D75.201 | 血小板增多 | 822 |
| S00.302 | 鼻挫伤 | 821 |
| Q53.902 | 睾丸下降不全 | 817 |
| Q53.903 | 隐睾[症] | 814 |
| K07.400 | 安氏II类错合 | 813 |
| F90.901 | 注意力缺陷多动障碍 | 811 |
| J02.903 | 溃疡性咽炎 | 806 |
| S01.500 | 口腔开放性伤口 | 803 |
| Q43.105 | 先天性巨结肠(赫希施普隆氏病) | 802 |
| H93.100 | 耳鸣 | 793 |
| S52.202 | 尺骨骨折 | 792 |
| I84.901 | 痔 | 791 |
| A08.201 | 腺病毒肠炎 | 789 |
| D48.903 | 肿瘤 | 789 |
| J45.004 | 过敏性咳嗽 | 787 |
| H10.801 | 感染性结膜炎 | 785 |
| P21.900a | 新生儿窒息 | 782 |
| K03.800 | 牙震荡 | 778 |
| R22.902 | 皮下肿物 | 775 |
| M87.900 | 骨坏死 | 771 |
| R59.901 | 淋巴结反应性增生 | 768 |
| W13.900 | 从房屋或建筑结构上跌落或跌出，从建筑物上跌落 | 762 |
| H65.000 | 急性分泌性中耳炎 | 758 |
| H60.501 | 外耳湿疹 | 757 |
| S06.202 | 脑挫伤 | 756 |
| Q18.001 | 先天性鳃裂瘘管 | 755 |
| T56.001 | 高铅血症 | 750 |
| R09.001 | 窒息 | 749 |
| M89.823 | 骨痛 | 740 |
| Q61.400 | 肾发育不良 | 740 |
| R60.901 | 水肿 | 740 |
| K14.100 | 地图样舌 | 738 |
| H52.102 | 近视 | 735 |
| H52.201 | 散光 | 734 |
| M92200/0 | 软骨瘤 | 734 |
| K07.302 | 牙列不齐 | 733 |
| L81.900 | 皮肤色素沉着 | 733 |
| H02.806 | 眼睑水肿 | 732 |
| H05.901 | 眼眶肿物 | 730 |
| S73.001 | 髋关节半脱位 | 728 |
| L30.500x002 | 单纯糠疹 | 727 |
| E44.101 | 轻度营养不良 | 722 |
| L51.900 | 多形红斑 | 720 |
| R26.802 | 下肢跛行 | 720 |
| B30.201+H13.1* | 咽结膜热 | 714 |
| Q70.900 | 并指[趾]畸形 | 712 |
| F41.101 | 焦虑状态 | 709 |
| J38.200 | 声带结节 | 704 |
| S02.200 | 鼻骨骨折 | 704 |
| Q74.102 | 先天性膝外翻 | 702 |
| Z98.800x405 | 先天性心脏病术后 | 698 |
| N28.101 | 肾囊肿 | 697 |
| B89.x00a | 寄生虫感染 | 694 |
| K75.804 | 胆汁淤积性肝炎 | 694 |
| B86 01 | 疥疮 | 693 |
| H50.900 | 斜视 | 691 |
| T69.100 | 冻疮 | 690 |
| H60.900 | 外耳炎 | 686 |
| Q82.507 | 疣状痣 | 686 |
| N62.x02 | 男性乳房发育 | 682 |
| R10.401 | 肠绞痛 | 682 |
| E30.900a | 青春期变异 | 681 |
| N76.101 | 阴道炎 | 680 |
| N62.x00 | 乳房肥大 | 665 |
| E04.103 | 甲状腺结节 | 663 |
| Q74.200 | 下肢畸形 | 658 |
| E74.002 | 葡萄糖-6-磷酸酶缺乏 | 655 |
| W44.901 | 异物进入眼或其它腔口 | 654 |
| R00.200 | 心悸 | 651 |
| K40.303 | 嵌顿性腹股沟疝 | 647 |
| Q31.500 | 先天性喉软骨软化病 | 646 |
| B00.904 | 疱疹感染 | 644 |
| Q21.300 | 法洛四联症 | 644 |
| F98.802 | 屏气发作 | 642 |
| S00.803 | 面部挫伤 | 642 |
| K12.002 | 口腔阿弗他溃疡 | 640 |
| Q18.002 | 先天性鳃裂囊肿 | 639 |
| R35 .01 | 神经性尿频 | 636 |
| Q68.002 | 先天性斜颈 | 635 |
| R72.x00x001 | 白细胞异常 | 635 |
| A02.05 | 蛲虫病 | 634 |
| G40.901 | 癫痫待排 | 632 |
| d23.900b | 毛母质瘤 | 629 |
| H16.202 | 干眼症 | 627 |
| H04.503 | 泪道梗阻 | 626 |
| K13.015 | 口角炎 | 623 |
| E70.101 | 高苯丙氨酸血症 | 619 |
| L30.100 | 汗疱疹 | 617 |
| N44.x00 | 睾丸附件扭转坏死 | 617 |
| F95.900 | 抽动障碍 | 615 |
| H50.403 | 上斜视 | 613 |
| J06.900a | 反复上呼吸道感染 | 613 |
| L02.901 | 皮肤疖 | 612 |
| R64.x00x002 | 消瘦 | 612 |
| G04.907 | 骨髓炎 | 606 |
| K04.701 | 根尖脓肿 | 605 |
| L74.300 | 痱 | 603 |
| Q66.801 | 先天性马蹄足 | 600 |
| L80.x00 | 白癜风 | 597 |
| R04.201 | 咯血 | 593 |
| I78.102 | 蜘蛛痣 | 588 |
| L81.500 | 白斑病 | 586 |
| J18.900 | 重症肺炎 | 585 |
| N01.900 | 肾炎综合症 | 581 |
| K26.901 | 十二指肠溃疡 | 572 |
| E80.604 | 高胆红素血症 | 571 |
| K83.102 | 胆汁淤积症 | 571 |
| Q33.001 | 先天性肺囊性病变 | 571 |
| D69.000x006+N08.2* | 过敏性紫癜性肾炎 | 566 |
| J35.302 | 扁桃体和腺样体肥大 | 565 |
| M60.900 | 肌炎 | 565 |
| B35.402 | 体癣 | 564 |
| L30.903 | 外阴湿疹 | 563 |
| M85.691 | 骨囊肿 | 559 |
| P92.000 | 新生儿呕吐 | 558 |
| D66.x02 | 血友病 | 557 |
| M95.401 | 后天性胸廓畸形 | 551 |
| J69.001 | 吸入性肺炎 | 544 |
| D72.801 | 白细胞增多症 | 542 |
| E02.x00 | 亚临床甲状腺功能减退症 | 537 |
| M25.561 | 膝关节痛 | 537 |
| R47.802 | 言语障碍 | 537 |
| J20.904 | 急性喉气管支气管炎 | 534 |
| M94.808 | 软骨炎 | 529 |
| T56.001 | 血铅过高 | 521 |
| H55.02 | 眼球震颤 | 520 |
| K07.301 | 牙错位 | 510 |
| R26.802 | 走路不稳 | 509 |
| K92.901 | 胃食道返流 | 508 |
| T79.300x001 | 创伤后伤口感染 | 506 |
| F07.201 | 脑外伤后综合征 | 503 |
| H50.301 | 间歇性外斜视 | 501 |
| S00.501 | 口腔浅表损伤 | 500 |
| K13.705 | 口腔出血 | 497 |
| N34.201 | 尿道口炎 | 497 |
| P78.802 | 新生儿腹胀 | 493 |
| E72.900 | 氨基酸代谢紊乱 | 491 |
| G47.902 | 睡眠问题(非器质性) | 489 |
| J98.601 | 膈膨升 | 489 |
| K00.604 | 牙齿萌出过晚 | 489 |
| R22.005 | 面部肿物 | 489 |
| R23.201 | 皮肤潮红 | 489 |
| R10.102 | 胃痛 | 487 |
| S05.802 | 眼挫伤 | 487 |
| D17.902 | 脂肪瘤 | 486 |
| Z90.40x | 先天性巨结肠术后 | 486 |
| Q90.900 | 唐氏综合征 | 478 |
| Q65.200 | 先天性髋关节脱位 | 475 |
| B99.02 | 感染性发热 | 474 |
| K90.900 | 肠吸收不良 | 474 |
| S62.602 | 指骨骨折 | 474 |
| N50.809 | 阴囊肿大 | 473 |
| V89.202 | 上下车时非碰撞的机动车交通事故 | 470 |
| D69.005+ | 过敏性紫癜相关性肾炎 | 467 |
| H00.101 | 睑板腺囊肿(霰粒肿) | 465 |
| K44.901 | 食管裂孔疝 | 462 |
| L50.801 | 急性荨麻疹 | 462 |
| J98.411 | 肺囊肿 | 460 |
| Y30.900 | 从高处跌落、跳下或被推下，意图不确定 | 459 |
| K40.903 | 腹股沟滑动疝 | 458 |
| N47.x00 | 疤痕包茎 | 458 |
| Q99.900 | 染色体异常 | 458 |
| K11.204 | 慢性腮腺炎 | 457 |
| L81.300 | 咖啡牛奶色斑 | 456 |
| K80.200 | 胆囊结石 | 455 |
| L42.x00 | 玫瑰糠疹 | 455 |
| H00.002 | 睑腺炎(麦粒肿) | 444 |
| T75.400x00 | 电击伤 | 444 |
| N76.003 | 细菌性阴道炎 | 443 |
| Q54.901 | 男性尿道下裂 | 443 |
| Q69.901 | 多指[畸形] | 437 |
| T17.804 | 肺异物 | 434 |
| I84.501 | 外痔 | 432 |
| S06.804 | 创伤性颅内出血 | 432 |
| A49.902 | 细菌性感染 | 429 |
| S01.802 | 面部裂伤 | 425 |
| Q74.101 | 先天性膝内翻 | 424 |
| S30.100 | 腹壁挫伤 | 424 |
| Z35.902 | 高危新生儿 | 423 |
| N83.201 | 卵巢囊肿 | 422 |
| K09.900x001 | 口腔囊肿 | 421 |
| E88.905 | 代谢性疾病 | 420 |
| W45.x00 | 异物或物体经皮肤进入 | 419 |
| R73.900 | 高血糖 | 417 |
| R31.x00a | 单纯性血尿 | 416 |
| M25.700 | 骨赘 | 415 |
| G47.200 | 睡眠-觉醒节律障碍 | 414 |
| R19.002 | 腹部肿胀 | 412 |
| T17.400 | 气管内异物 | 408 |
| E23.200 | 尿崩症 | 405 |
| E87.205 | 有机酸血症 | 405 |
| T15.000 | 角膜异物 | 405 |
| R61.901 | 盗汗(中医) | 403 |
| S30.301 | 包皮撕裂伤 | 403 |
| E71.300 | 脂肪酸代谢紊乱 | 401 |
| H53.101 | 眼疲劳 | 398 |
| E71.302 | 原发性肉碱缺乏症 | 397 |
| F42.002 | 交叉性擦腿 | 394 |
| R06.502 | 打鼾 | 394 |
| M54.200 | 颈痛 | 392 |
| G71.000 | 肌营养不良症 | 389 |
| E61.900 | 营养元素缺乏 | 388 |
| K52.908a | 迁延性腹泻 | 385 |
| Q21.103 | 先天性卵圆孔未闭 | 385 |
| N92.200 | 青春期月经过多 | 384 |
| B07.x01 | 扁平疣 | 383 |
| K08.302 | 残冠 | 383 |
| K13.702 | 口腔肿物 | 382 |
| Q21.102 | 房间隔缺损（继发孔型） | 381 |
| E79.000 | 高尿酸血症 | 380 |
| H60.501 | 急性外耳道炎 | 378 |
| L51.202 | 热液和热气化合物含水蒸气烧或烫伤 | 377 |
| H61.201 | 耵聍嵌塞 | 376 |
| P51.900 | 新生儿脐带出血 | 376 |
| K09.808 | 口腔粘液腺囊肿 | 375 |
| K07.400 | 安氏I类错合 | 374 |
| K35.900 | 急性阑尾炎 | 374 |
| L02.801 | 头皮脓肿 | 374 |
| R56.001 | 高热惊厥 | 370 |
| S05.100x004 | 眼球挫伤 | 370 |
| M20.000 | 手指变形 | 369 |
| Q04.303 | 脑发育不全 | 368 |
| J04.104 | 喘息性气管炎 | 367 |
| K62.302 | 直肠脱垂 | 366 |
| R06.600 | 呃逆 | 364 |
| X40.900 | 药物过量意外中毒 | 362 |
| Z48.800f | 先天性巨结肠术后的随诊医疗 | 362 |
| G93.811 | 脑室扩张 | 361 |
| I62.900 | 颅内出血(原因不明) | 361 |
| S05.001 | 角膜擦伤 | 359 |
| E73.900 | 乳糖不耐受症 | 358 |
| M90800/1 | 畸胎瘤 | 357 |
| L72.101 | 皮脂囊肿 | 356 |
| L53.000 | 中毒性红斑 | 354 |
| R58.x02 | 瘀斑 | 354 |
| R53.x00x002a | 乏力待查 | 352 |
| L85.802 | 毛发角化病 | 349 |
| N47.x02 | 包皮嵌顿 | 348 |
| S08.000 | 头皮撕脱 | 348 |
| S42.402 | 肱骨外髁骨折 | 348 |
| T16.x00 | 外耳异物 | 348 |
| Q56.000x001 | 两性畸形 | 346 |
| A49.300 | 支原体感染 | 345 |
| K14.000 | 舌炎 | 345 |
| H10.103 | 泡性结膜炎 | 342 |
| K44.900 | 膈疝 | 342 |
| M76.603 | 跟腱痛 | 341 |
| P39.100 | 新生儿结膜炎 | 341 |
| Q75.000 | 颅缝早闭 | 340 |
| S93.601 | 足扭伤 | 340 |
| W13.x00a | 意外坠落 | 340 |
| A02.01 | 蛔虫病 | 337 |
| E83.504 | 高钙尿症 | 337 |
| I10.x00 | 高血压病 | 336 |
| F79.903 | 语言发育迟缓 | 334 |
| B26.900 | 流行性腮腺炎 | 333 |
| L91.801 | 皮肤赘生物 | 333 |
| M24.491 | 关节习惯性脱位 | 333 |
| D56.900 | 地中海贫血 | 332 |
| K59.002 | 粪便潴留 | 332 |
| H11.105 | 结膜结石 | 331 |
| H61.105 | 耳廓肿物 | 330 |
| L68.900 | 多毛 | 329 |
| K62.101 | 直肠息肉 | 326 |
| L04.904 | 急性淋巴结炎 | 326 |
| J02.902 | 急性咽峡炎 | 325 |
| Q76.801 | 先天性胸廓发育畸形 | 325 |
| N45.903 | 附睾炎 | 322 |
| K58.900x003 | 肠易激综合征 | 321 |
| P52.000a | 室管膜下出血不伴有脑室内扩散 | 319 |
| K91.401 | 肠造瘘术后 | 318 |
| L81.200 | 雀斑 | 317 |
| Q63.001 | 重复肾 | 317 |
| E44.000 | 中度营养不良 | 316 |
| J18.004 | 哮喘性支气管肺炎 | 316 |
| L20.805 | 擦烂红斑 | 316 |
| S53.001 | 桡肱关节脱位 | 316 |
| J34.808 | 鼻前庭炎 | 315 |
| D64.905 | 婴儿贫血 | 313 |
| K50.900 | 克罗恩病 | 312 |
| M91800/0 | 骨瘤 | 311 |
| L74.000 | 红痱 | 310 |
| K14.807 | 舌囊肿 | 309 |
| H92.200 | 耳出血 | 307 |
| E04.901 | 甲状腺肿 | 306 |
| K25.900x001 | 胃溃疡 | 306 |
| H26.901 | 白内障 | 304 |
| F95.201 | 抽动秽语综合征 | 303 |
| Q05.901 | 脑脊膜膨出 | 302 |
| N43.302 | 婴儿鞘膜积液 | 301 |
| S01.503 | 牙龈裂伤 | 301 |
| E03.802 | 甲状腺机能减退 | 300 |
| F79.901 | 智力障碍 | 300 |
| K85.07 | 急性胰腺炎 | 299 |
| Q74.104 | 膝内翻 | 299 |
| B35.301 | 足癣 | 297 |
| T14.701 | 挤压伤 | 295 |
| T17.501 | 支气管异物 | 295 |
| S10.901 | 颈部挫伤 | 293 |
| K03.600 | 牙齿沉积物[增积物] | 292 |
| K52.917 | 小儿肠炎 | 292 |
| K75.901 | 婴儿肝炎综合征 | 291 |
| I61.900 | 脑出血 | 289 |
| K61.200 | 肛门直肠脓肿 | 289 |
| M32.100 | 系统性红斑狼疮，累及器官或系统 | 289 |
| A86.x00 | 病毒性脑炎 | 288 |
| D61.903 | 血二系减少 | 288 |
| H61.103 | 耳廓囊肿 | 288 |
| Q65.201 | 先天性髋脱位术后(换石膏) | 287 |
| S53.002 | 尺桡关节脱位 | 286 |
| Q06.801 | 先天性脊髓栓系综合征 | 285 |
| F42.100 | 习惯性擦腿 | 284 |
| K80.800 | 胆石症 | 281 |
| D66.x01 | 血友病A型 | 279 |
| L92.903 | 肉芽肿 | 279 |
| S27.301 | 肺挫伤 | 279 |
| E05.901 | 甲状腺机能亢进 | 278 |
| Q05.900 | 脊柱裂 | 278 |
| B00.205 | 疱疹病毒性咽炎 | 277 |
| Q52.405 | 处女膜过长 | 277 |
| L27.202 | 食物过敏性皮炎 | 275 |
| N48.900 | 阴茎疾患 | 275 |
| Q96.900 | 特纳综合征 | 275 |
| T19.202 | 阴道内异物 | 275 |
| D61.900 | 再生障碍性贫血 | 273 |
| K40.306 | 单侧嵌顿性腹股沟斜疝 | 272 |
| P78.300x002 | 新生儿肠炎 | 271 |
| E16.200x001 | 低血糖症 | 270 |
| R49.801 | 语音障碍 | 269 |
| N76.000 | 急性阴道炎 | 268 |
| Q33.200 | 隔离肺 | 268 |
| G72.901 | 肌病 | 267 |
| N90.809 | 小阴唇粘连 | 267 |
| K14.101 | 地图舌 | 265 |
| M54.900 | 背痛 | 264 |
| H05.001 | 眶蜂窝织炎 | 261 |
| J35.900 | 扁桃体和腺样体病 | 261 |
| T50.901 | 药物中毒(反应) | 261 |
| N32.805 | 膀胱过度活跃综合症 | 260 |
| G04.919 | 小脑炎 | 259 |
| J18.900a | 急性肺炎 | 259 |
| H15.802 | 巩膜黑变病 | 258 |
| M95.410 | 后天性肋骨畸形 | 258 |
| H01.1 | 眼睑湿疹 | 257 |
| S00.401 | 耳廓挫伤 | 257 |
| S09.903 | 耳外伤(异物等) | 257 |
| A03.901 | 细菌性痢疾 | 256 |
| M43.601 | 肌性斜颈 | 256 |
| L00.01 | 新生儿脓疱病 | 255 |
| L53.900 | 红斑 | 254 |
| K05.000 | 急性牙龈炎 | 253 |
| P05.100 | 小于胎龄 | 253 |
| D58.901 | 溶血性贫血 | 252 |
| K20.x00 | 食管炎 | 251 |
| Q42.200 | 先天性肛门缺如、闭锁和狭窄，伴瘘 | 251 |
| Q62.501 | 双肾盂双输尿管 | 250 |
| N25.802 | 肾小管酸中毒 | 249 |
| N44.01 | 睾丸扭转 | 249 |
| M92100/0 | 骨软骨瘤 | 248 |
| I86.802 | 颈静脉扩张 | 247 |
| K40.309 | 腹股沟嵌顿性滑疝 | 247 |
| D68.900 | 凝血谱异常 | 246 |
| R22.101 | 颈部肿物 | 246 |
| K27.901 | 消化性溃疡 | 245 |
| Q42.300 | 先天性肛门缺如、闭锁和狭窄 | 244 |
| S05.303 | 结膜裂伤 | 242 |
| I49.400 | 早搏 | 241 |
| Q35.901 | 耳聋[症] | 241 |
| F81.002 | 学习困难 | 239 |
| Q64.400 | 脐尿管畸形 | 238 |
| Q87.106 | Prader-Willi综合征（普拉德-威利） | 238 |
| D61.903 | 血三系减少 | 237 |
| R22.100 | 颈部局部肿胀、肿物和肿块 | 237 |
| I42.900 | 心肌病 | 236 |
| O36.101 | ABO血型不合 | 236 |
| F45.403 | 功能性腹痛综合征 | 235 |
| I07.008 | 三尖瓣反流 | 235 |
| I10.x03 | 高血压1级 | 235 |
| S05.803 | 角膜损伤 | 235 |
| K76.001 | 脂肪肝 | 232 |
| E87.602 | 低钾血症 | 231 |
| K86.107 | 胰腺炎 | 231 |
| P77 01 | 新生儿坏死性结肠炎 | 230 |
| E61.100 | 铁缺乏 | 229 |
| L02.803 | 头部疖 | 228 |
| D23.400 | 头皮良性肿瘤 | 226 |
| S90.301 | 足挫伤 | 225 |
| J06.904 | 新生儿上呼吸道感染 | 224 |
| R22.001 | 头部局部肿物 | 224 |
| S06.400 | 创伤性硬膜外出血 | 224 |
| T81.208 | 肌腱断裂，操作中 | 222 |
| K12.003 | 疱疹样口炎 | 219 |
| M89.920 | 骨病变 | 219 |
| T75.101 | 溺水 | 219 |
| H04.508 | 鼻泪管狭窄 | 218 |
| M25.601 | 肢体僵硬 | 218 |
| NA | 脾虚(中医) | 218 |
| Q17.200 | 小耳畸形 | 217 |
| S60.202 | 手挫伤 | 217 |
| S14.300x001 | 臂丛神经损伤 | 216 |
| B35.601 | 股癣 | 215 |
| K43.902 | 腹壁疝 | 215 |
| M95.403 | 胸廓畸形 | 215 |
| N63.x01 | 乳房结节 | 213 |
| B49.x00x007 | 真菌感染 | 212 |
| L91.000 | 瘢痕疙瘩 | 211 |
| S83.601 | 膝关节扭伤 | 211 |
| H02.807 | 眼睑出血 | 210 |
| M79.100 | 肌痛 | 210 |
| R22.206 | 骶尾部肿物 | 210 |
| P81.902 | 新生儿发热 | 209 |
| D38.301 | 纵隔肿瘤 | 208 |
| E30.101 | 不完全性性早熟 | 208 |
| B25.100+K77.0* | 巨细胞病毒性肝炎 | 207 |
| B27.901 | 传染性单核细胞增多综合征 | 207 |
| R34.x01 | 少尿 | 207 |
| K62.200 | 脱肛 | 205 |
| R22.003 | 颞部肿物 | 205 |
| R63.000 | 食欲缺乏 | 205 |
| K81.901 | 胆囊炎 | 204 |
| M47.902 | 脊柱变性 | 204 |
| M95000/3 | 神经母细胞瘤 | 204 |
| K11.101 | 腮腺肥大 | 203 |
| N76.101 | 慢性阴道炎 | 201 |
| K62.815 | 肛周炎 | 199 |
| Q52.701 | 会阴裂 | 199 |
| S67.001 | 手指挤压伤 | 199 |
| M79.681 | 肢体疼痛 | 198 |
| F84.002 | 儿童期孤独症 | 197 |
| Q64.401 | 脐尿管瘘 | 196 |
| R63.300 | 婴幼儿母乳喂养问题 | 196 |
| R94.303 | 心电图异常 | 196 |
| G47.000 | 失眠 | 195 |
| J20.902 | 急性气管支气管炎 | 195 |
| J33.900 | 鼻息肉 | 195 |
| K62.401 | 肛门狭窄 | 195 |
| G25.301 | 肌阵挛 | 194 |
| H93.801 | 耳后肿物 | 194 |
| N36.807 | 尿道旁管囊肿 | 194 |
| S09.905 | 耳廓损伤 | 194 |
| M25.507 | 踝关节痛 | 193 |
| J45.004 | 过敏性鼻炎伴有哮喘 | 192 |
| N00.902 | 急性肾小球肾炎 | 192 |
| P77.x01 | 新生儿坏死性小肠结肠炎 | 191 |
| H91.903 | 听力障碍 | 190 |
| M32.901 | 系统性红斑狼疮 | 190 |
| N92.201 | 青春期出血(青春期月经过多) | 190 |
| Q61.300 | 多囊肾 | 189 |
| S52.300 | 孟氏骨折(闭合性) | 189 |
| H60.303 | 耳廓瘘感染 | 188 |
| S52.301 | 孟氏骨折 | 188 |
| K65.008 | 肝周脓肿 | 187 |
| M40.200 | 脊柱后凸 | 187 |
| Z38.500 | 双胎 | 187 |
| F95.201 | 多动秽语综合征 | 185 |
| J44.800 | 闭塞性(毛)细支气管炎 | 185 |
| K11.608 | 粘液腺囊肿(下唇) | 185 |
| K21.001 | 反流性食管炎 | 185 |
| R60.901 | 浮肿待查 | 185 |
| Z54.800x005 | 脑炎恢复期 | 185 |
| K60.401 | 直肠会阴瘘 | 183 |
| R05.x00 | 感染后咳嗽 | 183 |
| S30.206 | 阴茎挫伤 | 183 |
| E06.901 | 甲状腺炎 | 182 |
| M17.900 | 膝关节病 | 182 |
| N00.900 | 急性肾炎综合征 | 182 |
| Q52.500 | 阴唇融合 | 182 |
| K14.001 | 舌溃疡 | 181 |
| S00.802 | 面部擦伤 | 181 |
| K62.805 | 肛周感染 | 180 |
| G70.001 | 重症肌无力(眼肌型) | 179 |
| G71.201 | 先天性肌病 | 179 |
| T78.101 | 牛奶过敏反应 | 179 |
| B35.001 | 头癣 | 178 |
| P55.100 | 新生儿ABO溶血症 | 178 |
| S00.202 | 眼睑血肿 | 178 |
| T04.400 | 上肢伴下肢多部位挤压伤 | 178 |
| T30.000x00 | 烧伤 | 178 |
| I83.906 | 静脉曲张 | 177 |
| Q40.001 | 先天性肥大性幽门狭窄 | 177 |
| R22.203 | 腹壁肿物 | 177 |
| Z54.800 | 化脓性脑膜炎恢复期 | 177 |
| G70.903 | 肌无力 | 176 |
| M24.400 | 关节复发性脱位和不全脱位 | 176 |
| I37.000 | 肺动脉瓣狭窄 | 175 |
| R26.802 | 跛行待查 | 175 |
| K02.400b | 牙冠折断(复杂冠折) | 174 |
| Q17.900 | 先天性耳畸形 | 174 |
| S37.002 | 肾挫伤 | 174 |
| T17.202 | 喉、咽异物 | 174 |
| L00.03 | 金黄色葡萄球菌烫伤样综合症 | 173 |
| N36.806 | 尿道囊肿 | 173 |
| S01.506 | 口腔外伤 | 173 |
| E30.003 | 性发育落后 | 172 |
| G71.001 | 进行性肌营养不良 | 172 |
| L40.900 | 银屑病 | 172 |
| R74.803 | 心肌酶谱异常 | 172 |
| S39.905 | 腹部损伤 | 172 |
| E03.001 | 先天性甲状腺肿 | 170 |
| H01.901 | 眼睑炎 | 170 |
| K05.105 | 牙龈炎 | 170 |
| M87.901 | 股骨头缺血性坏死 | 170 |
| N48.802 | 包皮囊肿 | 170 |
| Q66.601 | 先天性马蹄外翻足 | 170 |
| R16.000 | 肝大 | 170 |
| D72.100 | 嗜酸细胞增多症 | 169 |
| G93.821 | 硬膜下积液 | 169 |
| N49.202 | 阴囊疖肿 | 169 |
| A09.902 | 结肠炎 | 168 |
| L68.900 | 多毛症 | 168 |
| N13.701 | 膀胱输尿管反流 | 168 |
| Q44.301 | 先天性胆管闭塞性黄疸 | 168 |
| R59.011 | 腹腔淋巴结肿大 | 168 |
| L73.803 | 皮脂腺增生 | 167 |
| M24.300 | 关节病理性脱位和不全脱位 | 167 |
| J93.900 | 气胸 | 166 |
| S30.205 | 阴囊挫伤 | 166 |
| P83.801 | 新生儿红斑 | 165 |
| R30.000 | 排尿困难 | 165 |
| T29.200 | 多处二度烧(烫)伤 | 165 |
| K00.703 | 乳牙迟萌 | 164 |
| K04.002 | 急性牙髓炎 | 164 |
| D48.001 | 骨肿瘤 | 162 |
| H60.403 | 外耳道肉芽肿 | 162 |
| H70.001 | 耳后脓肿 | 162 |
| P28.201 | 新生儿青紫 | 162 |
| P70.401 | 新生儿低血糖症 | 162 |
| R22.202 | 胸壁肿物 | 162 |
| W19.900 | 跌倒 | 162 |
| Q18.000 | 先天性鳃裂窦 | 161 |
| R00.100 | 心动过缓 | 161 |
| R22.004 | 颊部肿物 | 161 |
| S92.301 | 跖骨骨折 | 161 |
| D18.013 | 肝血管瘤 | 160 |
| H04.201 | 泪溢 | 160 |
| R06.500 | 口呼吸 | 160 |
| A09.903 | 婴儿腹泻 | 159 |
| F45.300 | 自主神经功能紊乱 | 159 |
| G00.901 | 化脓性脑膜炎 | 159 |
| N05.301 | 系膜增生性肾小球肾炎 | 159 |
| F45.306 | 心脏神经官能症 | 158 |
| H65.903 | 渗出性中耳炎 | 158 |
| H72.902 | 鼓膜穿孔 | 158 |
| K06.804 | 牙龈出血 | 157 |
| R13.x00 | 吞咽困难 | 157 |
| R22.900 | 局部肿胀、肿物和肿块 | 157 |
| R33.x00 | 尿潴留 | 156 |
| H18.806 | 角膜上皮脱落 | 155 |
| K00.200x002a | 上皮珠 | 155 |
| Q18.101 | 先天性耳廓瘘 | 155 |
| R22.006 | 耳部肿物 | 155 |
| P22.002 | 新生儿呼吸窘迫综合征 | 154 |
| R47.102 | 发音不清 | 154 |
| S05.101 | 眼眶挫伤 | 154 |
| T14.003 | 皮下血肿 | 154 |
| E23.013 | 生长激素缺乏(低下) | 153 |
| I28.803 | 肺动脉狭窄 | 153 |
| E34.300x012 | 身材矮小症 | 152 |
| K08.801 | 牙痛 | 151 |
| N34.204 | 急性尿道炎 | 151 |
| R42.x00 | 眩晕 | 151 |
| H35.900 | 视网膜疾患 | 150 |
| L60.100 | 甲剥离 | 150 |
| Q60.500 | 肾发育不全 | 150 |
| R19.002 | 盆腔肿物 | 150 |
| R56.001 | 热性惊厥附加症 | 150 |
| E06.304 | 桥本甲状腺炎 | 149 |
| E74.201 | 半乳糖血症 | 149 |
| R63.001 | 厌食(食欲缺乏) | 149 |
| F50.401 | 婴儿喂养障碍(喂养困难) | 148 |
| G03.900 | 脑膜炎 | 148 |
| N94.600 | 痛经 | 147 |
| S13.601 | 颈部扭伤 | 147 |
| G93.102 | 缺氧缺血性脑病 | 146 |
| R16.100x001 | 脾大 | 146 |
| S30.205 | 阴囊外伤 | 146 |
| C95.901 | 白血病 | 145 |
| J94.804 | 胸腔积液 | 145 |
| S01.802 | 面部外伤 | 144 |
| E27.803 | 肾上腺皮质增生 | 143 |
| K22.104 | 食管溃疡 | 143 |
| R40.200 | 昏迷 | 143 |
| D68.903 | 凝血时间延长 | 142 |
| H60.500x002 | 外耳道湿疹 | 142 |
| R22.204 | 腹股沟肿物 | 142 |
| H35.100 | 早产儿视网膜病变 | 141 |
| K12.203 | 颊部脓肿 | 141 |
| Z48.800h | 食道闭锁术后的随诊医疗 | 141 |
| H04.302 | 急性泪囊炎 | 140 |
| Q66.801 | 马蹄足术后(换石膏) | 140 |
| J12.000 | 腺病毒肺炎 | 139 |
| Q69.901 | (多趾[畸形]) | 139 |
| G93.002 | 透明隔囊肿 | 138 |
| K76.901 | 肝肿物 | 138 |
| D70.x00 | 粒细胞缺乏 | 137 |
| J21.900 | 急性细支气管炎 | 137 |
| L30.904 | 阴囊湿疹 | 136 |
| R27.000 | 共济失调 | 136 |
| J18.000x002 | 毛细管支气管性肺炎 | 135 |
| K00.605 | 乳齿过早脱落 | 135 |
| L80.01 | 白斑(白癜风) | 135 |
| G93.001 | [脑]蛛网膜囊肿 | 134 |
| H52.001 | 远视 | 134 |
| G70.000 | 重症肌无力 | 133 |
| H01.101 | 眼睑皮炎 | 133 |
| N50.811 | 睾丸疼痛 | 133 |
| N90.900 | 外阴非炎性疾病 | 133 |
| Q44.400 | 先天性胆总管囊肿 | 133 |
| H61.901 | 外耳道肿物 | 131 |
| I87.117 | 胡桃夹综合征 | 131 |
| L50.802 | 慢性荨麻疹 | 131 |
| M79.292 | 神经炎 | 131 |
| Q10.303 | 内眦赘皮 | 131 |
| R94.400 | 肾功能异常 | 131 |
| I27.001 | 肺动脉高压 | 130 |
| S05.901 | 眼损伤 | 130 |
| M76.600 | 跟腱炎 | 129 |
| N05.900x003 | 肾炎 | 129 |
| Q43.400 | 肠重复畸形 | 129 |
| Q87.806a | 心面综合征(歪嘴哭综合征) | 129 |
| K56.701 | 不完全性肠梗阻 | 128 |
| M25.502 | 肘关节痛 | 128 |
| M60.992 | 肌炎(无瘫痪) | 128 |
| M72.991 | 纤维瘤 | 128 |
| Q31.803 | 喉软骨软化 | 128 |
| Q42.301 | 先天性肛门闭锁 | 128 |
| K63.500 | 结肠息肉 | 127 |
| N34.200x002 | 尿道炎 | 127 |
| E14.900x00 | 糖尿病 | 126 |
| K91.201 | 短肠综合征(肠切除后综合征) | 126 |
| NA | 自汗(中医) | 126 |
| K07.203 | 前牙反合 | 125 |
| K22.206 | 食管狭窄 | 125 |
| N45.902 | 睾丸炎 | 125 |
| Q63.002 | 双肾双肾盂 | 125 |
| Q82.502 | 血管痣 | 125 |
| E72.202 | 瓜氨酸血症 | 124 |
| J98.400 | 支气管肺发育不良 | 124 |
| Q66.000 | 马蹄内翻足 | 124 |
| G40.400x001b | 婴儿痉挛症 | 123 |
| H10.102 | 春季卡他性结膜炎 | 123 |
| K62.900 | 肛门和直肠疾患 | 123 |
| M72.591 | 筋膜炎 | 123 |
| R23.301 | 皮下出血 | 123 |
| D68.801 | 凝血因子缺乏 | 122 |
| E30.801 | 乳腺过早发育 | 122 |
| H11.900 | 结膜疾患 | 122 |
| K06.803 | 牙龈瘤 | 122 |
| K43.900 | 腹白线疝 | 122 |
| P78.300x00 | 新生儿腹泻 | 122 |
| B02.900 | 带状疱疹 | 121 |
| K21.901 | 食管反流 | 121 |
| P54.900 | 新生儿出血 | 121 |
| Q43.105 | 先天性巨结肠类源病 | 121 |
| D20.103 | 脐尿管囊肿 | 120 |
| S01.201 | 鼻开放性外伤 | 120 |
| F71.901 | 智力缺欠(或低下) | 119 |
| H01.000 | 睑缘炎 | 119 |
| S53.402 | 肘关节扭伤 | 119 |
| T18.200 | 胃内异物 | 119 |
| E83.001 | 肝豆状核变性(威尔逊氏病) | 118 |
| H66.400 | 化脓性中耳炎 | 118 |
| K21.903 | 喉咽反流 | 118 |
| K55.004b | 坏死性小肠结肠炎 | 118 |
| K76.811 | 肝囊肿 | 118 |
| K91.402 | 肠造口术后功能障碍 | 118 |
| I97.102 | 先天性心脏病术后心功能不全 | 117 |
| M67.001 | 跟腱挛缩 | 117 |
| Q28.901 | 先天性心血管畸形 | 117 |
| Q55.604 | 先天性阴茎下弯 | 117 |
| K83.817 | 胆总管扩张 | 116 |
| S00.802 | 面部软组织挫伤 | 116 |
| K52.804 | 嗜酸细胞性胃肠炎 | 115 |
| K62.901 | 肛旁肿物 | 115 |
| K71.600x002 | 中毒性肝炎 | 115 |
| R22.205 | 背部肿物 | 115 |
| H46.x00 | 视神经炎 | 114 |
| I84.202 | 内痔 | 114 |
| L84.x00x002 | 胼胝 | 114 |
| M21.161 | 后天性膝内翻 | 114 |
| H71.06 | 中耳胆脂瘤 | 113 |
| K14.901 | 舌肿物 | 113 |
| R42.x00 | 头晕和眩晕 | 113 |
| R49.001 | 声音嘶哑 | 113 |
| Z54.000 | 手术后恢复期 | 113 |
| I78.801 | 毛细血管扩张症 | 112 |
| K10.808 | 上腭穿孔 | 112 |
| N35.900 | 尿道狭窄 | 112 |
| K76.903 | 肝占位性病变 | 111 |
| L00.x00 | 葡萄球菌性烫伤样皮肤综合征 | 111 |
| M08.900 | 幼年特发性关节炎 | 111 |
| Q65.200 | 先天性髋关节脱位DDH | 111 |
| T29.001 | 多处烧伤 | 111 |
| E83.103 | 肺含铁血黄素沉积症 | 110 |
| K05.100 | 慢性牙龈炎 | 110 |
| S01.103 | 眼睑皮肤裂伤 | 110 |
| S20.200 | 胸部挫伤 | 110 |
| D31.001 | 结膜良性肿瘤 | 109 |
| G71.100 | 非癫痫性强直样发作 | 108 |
| R56.800a | 胃肠炎相关性惊厥 | 108 |
| S50.000 | 肘部挫伤 | 108 |
| Z86.702 | 脑出血个人史 | 108 |
| M85.200 | 颅骨肥大 | 107 |
| R63.101 | 多饮 | 107 |
| L84.x00 | 鸡眼 | 106 |
| M81.901 | 骨质疏松 | 106 |
| Q43.301 | 先天性肠旋转不良 | 106 |
| Q63.201 | 异位肾 | 106 |
| D55.001 | 蚕豆病 | 105 |
| G41.900 | 癫痫持续状态 | 105 |
| M16.200 | 发育异常性双侧髋关节病 | 105 |
| D72.806 | 淋巴细胞增多症 | 104 |
| E83.308 | 维生素Ｄ依赖性佝偻病 | 104 |
| K29.900 | 胃十二指肠炎 | 104 |
| Q02.x00 | 小头畸形 | 104 |
| T29.000 | 多处烧(烫)伤 | 104 |
| P22.101 | 新生儿湿肺 | 103 |
| P61.201 | 早产儿贫血 | 103 |
| S09.905 | 头皮撕裂伤 | 103 |
| D76.301 | 黄色肉芽肿 | 102 |
| G93.409 | 新生儿脑病 | 102 |
| S02.902 | 颅骨凹陷性骨折 | 102 |
| S05.601 | 角膜穿通伤 | 102 |
| D80.901 | 免疫球蛋白缺乏 | 101 |
| I47.112 | 阵发性室上性心动过速 | 101 |
| P05.001 | 低体重儿 | 101 |
| Q18.003 | 梨状窝瘘 | 101 |
| T14.400 | 神经损伤 | 101 |
| G04.201 | 化脓性脑膜脑炎 | 100 |
| G04.907 | 脊髓炎 | 100 |
| H04.602 | 泪囊瘘 | 100 |
| K66.802 | 肠系膜囊肿 | 100 |
| Q44.503 | 先天性胆总管扩张 | 100 |

Table S3 All eczema concomitant phenotypes in rare diseases.

| **hpo_id** | **hpo_term** | **classfication** | **Count^#^** | **Score^*^** |
| --- | --- | --- | --- | --- |
| HP:0000964 | Eczema | (Abnormality of the integument);(Abnormality of the immune system); | 39 | 18.55 |
| HP:0001249 | Intellectual disability | (Abnormality of the nervous system ); | 13 | 10.3 |
| HP:0001263 | Global developmental delay | (Abnormality of the nervous system ); | 13 | 9.95 |
| HP:0004322 | Short stature | (Growth abnormality ); | 12 | 7.5 |
| HP:0010783 | Erythema | (Abnormality of the integument );(Abnormality of the cardiovascular system ); | 10 | 7.2 |
| HP:0001250 | Seizures | (Abnormality of the nervous system ); | 12 | 7.15 |
| HP:0001252 | Muscular hypotonia | (Abnormality of the musculature ); | 9 | 6.25 |
| HP:0002205 | Recurrent respiratory infections | (Abnormality of the respiratory system );(Abnormality of the immune system ); | 8 | 5.8 |
| HP:0000164 | Abnormality of the teeth | (Abnormality of head and neck ); | 9 | 5.55 |
| HP:0001510 | Growth delay | (Growth abnormality ); | 8 | 5.4 |
| HP:0000958 | Dry skin | (Abnormality of the integument ); | 8 | 5 |
| HP:0000252 | Microcephaly | (Abnormality of the nervous system );(Abnormality of the skeletal system );(Abnormality of head and neck ); | 7 | 4.85 |
| HP:0002024 | Malabsorption | (Abnormality of the digestive system ); | 8 | 4.65 |
| HP:0008064 | Ichthyosis | (Abnormality of the integument ); | 7 | 4.5 |
| HP:0006482 | Abnormality of dental morphology | (Abnormality of head and neck ); | 5 | 4.5 |
| HP:0200034 | Papule | (Abnormality of the integument ); | 7 | 4.45 |
| HP:0002719 | Recurrent infections | (Abnormality of the immune system ); | 8 | 4.3 |
| HP:0007400 | Irregular hyperpigmentation | (Abnormality of the integument ); | 6 | 4.3 |
| HP:0000028 | Cryptorchidism | (Abnormality of the genitourinary system ); | 8 | 4.3 |
| HP:0008066 | Abnormal blistering of the skin | (Abnormality of the integument ); | 6 | 4.3 |
| HP:0001025 | Urticaria | (Abnormality of the integument );(Abnormality of the cardiovascular system ); | 8 | 4.2 |
| HP:0000989 | Pruritus | (Abnormality of the integument ); | 5 | 4.15 |
| HP:0000708 | Behavioral abnormality | (Abnormality of the nervous system ); | 5 | 4.15 |
| HP:0001596 | Alopecia | (Abnormality of the integument ); | 7 | 4.15 |
| HP:0000316 | Hypertelorism | (Abnormality of head and neck );(Abnormality of the eye ); | 7 | 4.1 |
| HP:0000508 | Ptosis | (Abnormality of the eye ); | 6 | 4 |
| HP:0000365 | Hearing impairment | (Abnormality of the ear ); | 7 | 3.75 |
| HP:0001597 | Abnormality of the nail | (Abnormality of the integument ); | 7 | 3.75 |
| HP:0000311 | Round face | (Abnormality of head and neck ); | 4 | 3.6 |
| HP:0000286 | Epicanthus | (Abnormality of head and neck ); | 8 | 3.55 |
| HP:0001873 | Thrombocytopenia | (Abnormality of blood and blood-forming tissues ); | 7 | 3.35 |
| HP:0000325 | Triangular face | (Abnormality of head and neck ); | 4 | 3.25 |
| HP:0000963 | Thin skin | (Abnormality of the integument ); | 4 | 3.25 |
| HP:0000750 | Delayed speech and language development | (Abnormality of the nervous system ); | 4 | 3.25 |
| HP:0002750 | Delayed skeletal maturation | (Abnormality of the skeletal system ); | 6 | 3.2 |
| HP:0000486 | Strabismus | (Abnormality of the eye ); | 5 | 3.05 |
| HP:0002099 | Asthma | (Abnormality of the respiratory system );(Abnormality of the immune system ); | 5 | 3 |
| HP:0000347 | Micrognathia | (Abnormality of head and neck );(Abnormality of the skeletal system ); | 6 | 2.9 |
| HP:0012733 | Macule | (Abnormality of the integument ); | 4 | 2.85 |
| HP:0000494 | Downslanted palpebral fissures | (Abnormality of head and neck ); | 6 | 2.85 |
| HP:0002650 | Scoliosis | (Abnormality of the skeletal system ); | 8 | 2.8 |
| HP:0200042 | Skin ulcer | (Abnormality of the integument ); | 6 | 2.8 |
| HP:0000463 | Anteverted nares | (Abnormality of head and neck ); | 5 | 2.7 |
| HP:0002007 | Frontal bossing | (Abnormality of head and neck );(Abnormality of the skeletal system ); | 5 | 2.7 |
| HP:0002705 | High, narrow palate | (Abnormality of head and neck ); | 3 | 2.7 |
| HP:0003212 | Increased IgE level | (Abnormality of blood and blood-forming tissues );(Abnormality of metabolism/homeostasis );(Abnormality of the immune system ); | 3 | 2.7 |
| HP:0001006 | Hypotrichosis | (Abnormality of the integument ); | 3 | 2.7 |
| HP:0000389 | Chronic otitis media | (Abnormality of the ear );(Abnormality of the immune system ); | 5 | 2.65 |
| HP:0002223 | Absent eyebrow | (Abnormality of head and neck );(Abnormality of the integument ); | 4 | 2.55 |
| HP:0002721 | Immunodeficiency | (Abnormality of the immune system ); | 4 | 2.55 |
| HP:0000343 | Long philtrum | (Abnormality of head and neck ); | 4 | 2.55 |
| HP:0000246 | Sinusitis | (Abnormality of the skeletal system );(Abnormality of head and neck );(Abnormality of the immune system ); | 4 | 2.5 |
| HP:0000982 | Palmoplantar keratoderma | (Abnormality of the integument ); | 4 | 2.5 |
| HP:0001508 | Failure to thrive | (Growth abnormality ); | 6 | 2.45 |
| HP:0001328 | Specific learning disability | (Abnormality of the nervous system ); | 3 | 2.35 |
| HP:0002960 | Autoimmunity | (Abnormality of the immune system ); | 3 | 2.35 |
| HP:0000506 | Telecanthus | (Abnormality of head and neck ); | 3 | 2.35 |
| HP:0001231 | Abnormality of the fingernails | (Abnormality of the integument ); | 5 | 2.35 |
| HP:0001595 | Abnormality of the hair | (Abnormality of the integument ); | 3 | 2.35 |
| HP:0008391 | Dystrophic fingernails | (Abnormality of the integument ); | 3 | 2.35 |
| HP:0001513 | Obesity | (Growth abnormality ); | 3 | 2.35 |
| HP:0008404 | Nail dystrophy | (Abnormality of the integument ); | 3 | 2.35 |
| HP:0005692 | Joint hyperflexibility | (Abnormality of the skeletal system ); | 5 | 2.3 |
| HP:0000470 | Short neck | (Abnormality of head and neck );(Abnormality of the skeletal system ); | 5 | 2.3 |
| HP:0001156 | Brachydactyly syndrome | (Abnormality of the skeletal system );(Abnormality of limbs ); | 4 | 2.15 |
| HP:0001511 | Intrauterine growth retardation | (Growth abnormality ); | 4 | 2.15 |
| HP:0000369 | Low-set ears | (Abnormality of the ear ); | 4 | 2.15 |
| HP:0200055 | Small hand | (Abnormality of the skeletal system );(Abnormality of limbs ); | 4 | 2.15 |
| HP:0001903 | Anemia | (Abnormality of blood and blood-forming tissues ); | 6 | 2.1 |
| HP:0000992 | Cutaneous photosensitivity | (Abnormality of the integument ); | 4 | 2.1 |
| HP:0006101 | Finger syndactyly | (Abnormality of the skeletal system );(Abnormality of limbs ); | 6 | 2.1 |
| HP:0000047 | Hypospadias | (Abnormality of the genitourinary system ); | 3 | 2 |
| HP:0000368 | Low-set, posteriorly rotated ears | (Abnormality of the ear ); | 3 | 2 |
| HP:0002209 | Sparse scalp hair | (Abnormality of head and neck );(Abnormality of the integument ); | 3 | 2 |
| HP:0002213 | Fine hair | (Abnormality of the integument ); | 3 | 2 |
| HP:0005280 | Depressed nasal bridge | (Abnormality of head and neck ); | 3 | 2 |
| HP:0002019 | Constipation | (Abnormality of the digestive system ); | 3 | 2 |
| HP:0002119 | Ventriculomegaly | (Abnormality of the nervous system ); | 3 | 2 |
| HP:0001373 | Joint dislocation | (Abnormality of the skeletal system ); | 3 | 2 |
| HP:0000233 | Thin vermilion border | (Abnormality of head and neck ); | 3 | 2 |
| HP:0000966 | Hypohidrosis | (Abnormality of the integument ); | 3 | 2 |
| HP:0001053 | Hypopigmented skin patches | (Abnormality of the integument ); | 3 | 2 |
| HP:0001824 | Weight loss | (Growth abnormality ); | 3 | 1.95 |
| HP:0000988 | Skin rash | (Abnormality of the integument );(Abnormality of the immune system ); | 3 | 1.95 |
| HP:0001945 | Fever | (Abnormality of metabolism/homeostasis ); | 3 | 1.95 |
| HP:0000160 | Narrow mouth | (Abnormality of head and neck ); | 5 | 1.95 |
| HP:0000232 | Everted lower lip vermilion | (Abnormality of head and neck ); | 3 | 1.95 |
| HP:0000023 | Inguinal hernia | (Abnormality of connective tissue );(Abnormality of the digestive system ); | 5 | 1.9 |
| HP:0000388 | Otitis media | (Abnormality of the ear );(Abnormality of the immune system ); | 2 | 1.8 |
| HP:0006510 | Chronic obstructive pulmonary disease | (Abnormality of the respiratory system ); | 2 | 1.8 |
| HP:0001770 | Toe syndactyly | (Abnormality of the skeletal system );(Abnormality of limbs ); | 4 | 1.8 |
| HP:0007018 | Attention deficit hyperactivity disorder | (Abnormality of the nervous system ); | 4 | 1.8 |
| HP:0000175 | Cleft palate | (Abnormality of head and neck ); | 4 | 1.8 |
| HP:0003103 | Abnormal cortical bone morphology | (Abnormality of the skeletal system ); | 2 | 1.8 |
| HP:0200043 | Verrucae | (Abnormality of the integument );(Neoplasm ); | 2 | 1.8 |
| HP:0001810 | Dystrophic toenail | (Abnormality of the integument ); | 2 | 1.8 |
| HP:0006323 | Premature loss of primary teeth | (Abnormality of head and neck ); | 2 | 1.8 |
| HP:0000670 | Carious teeth | (Abnormality of head and neck ); | 4 | 1.8 |
| HP:0001999 | Abnormal facial shape | (Abnormality of head and neck ); | 2 | 1.8 |
| HP:0001290 | Generalized hypotonia | (Abnormality of the musculature ); | 2 | 1.8 |
| HP:0000581 | Blepharophimosis | (Abnormality of head and neck ); | 3 | 1.65 |
| HP:0003196 | Short nose | (Abnormality of head and neck ); | 3 | 1.65 |
| HP:0000938 | Osteopenia | (Abnormality of the skeletal system ); | 3 | 1.65 |
| HP:0100760 | Clubbing of toes | (Abnormality of the skeletal system );(Abnormality of limbs ); | 3 | 1.65 |
| HP:0001935 | Microcytic anemia | (Abnormality of blood and blood-forming tissues ); | 3 | 1.6 |
| HP:0000230 | Gingivitis | (Abnormality of head and neck ); | 3 | 1.6 |
| HP:0000154 | Wide mouth | (Abnormality of head and neck ); | 3 | 1.6 |
| HP:0000684 | Delayed eruption of teeth | (Abnormality of head and neck ); | 3 | 1.6 |
| HP:0000767 | Pectus excavatum | (Abnormality of the skeletal system ); | 3 | 1.6 |
| HP:0000174 | Abnormality of the palate | (Abnormality of head and neck ); | 3 | 1.6 |
| HP:0000256 | Macrocephaly | (Abnormality of head and neck );(Abnormality of the skeletal system ); | 3 | 1.6 |
| HP:0004397 | Ectopic anus | (Abnormality of the digestive system ); | 3 | 1.6 |
| HP:0002097 | Emphysema | (Abnormality of the respiratory system ); | 3 | 1.6 |
| HP:0000929 | Abnormality of the skull | (Abnormality of head and neck );(Abnormality of the skeletal system ); | 3 | 1.6 |
| HP:0000682 | Abnormality of dental enamel | (Abnormality of connective tissue );(Abnormality of the skeletal system );(Abnormality of head and neck ); | 3 | 1.6 |
| HP:0001096 | Keratoconjunctivitis | (Abnormality of head and neck );(Abnormality of the eye );(Abnormality of the immune system ); | 3 | 1.6 |
| HP:0012471 | Thick vermilion border | (Abnormality of head and neck ); | 3 | 1.6 |
| HP:0001760 | Abnormality of the foot | (Abnormality of limbs ); | 3 | 1.6 |
| HP:0000967 | Petechiae | (Abnormality of the integument );(Abnormality of the cardiovascular system );(Abnormality of blood and blood-forming tissues ); | 2 | 1.45 |
| HP:0000978 | Bruising susceptibility | (Abnormality of the integument );(Abnormality of the cardiovascular system );(Abnormality of blood and blood-forming tissues ); | 2 | 1.45 |
| HP:0001888 | Lymphopenia | (Abnormality of blood and blood-forming tissues );(Abnormality of the immune system ); | 2 | 1.45 |
| HP:0000260 | Wide anterior fontanel | (Abnormality of head and neck );(Abnormality of the skeletal system ); | 2 | 1.45 |
| HP:0000270 | Delayed cranial suture closure | (Abnormality of head and neck );(Abnormality of the skeletal system ); | 2 | 1.45 |
| HP:0000319 | Smooth philtrum | (Abnormality of head and neck ); | 2 | 1.45 |
| HP:0001634 | Mitral valve prolapse | (Abnormality of the cardiovascular system ); | 2 | 1.45 |
| HP:0002615 | Hypotension | (Abnormality of the cardiovascular system ); | 2 | 1.45 |
| HP:0010318 | aplasia/Hypoplasia of the abdominal wall musculature | (Abnormality of the musculature );(Abnormality of the digestive system ); | 2 | 1.45 |
| HP:0002757 | Recurrent fractures | (Abnormality of the skeletal system ); | 2 | 1.45 |
| HP:0001072 | Thickened skin | (Abnormality of the integument ); | 2 | 1.45 |
| HP:0001880 | Eosinophilia | (Abnormality of blood and blood-forming tissues );(Abnormality of the immune system ); | 2 | 1.45 |
| HP:0009886 | Trichorrhexis nodosa | (Abnormality of the integument ); | 2 | 1.45 |
| HP:0002714 | Downturned corners of mouth | (Abnormality of head and neck ); | 2 | 1.45 |
| HP:0004279 | Short palm | (Abnormality of the skeletal system );(Abnormality of limbs ); | 2 | 1.45 |
| HP:0000239 | Large fontanelles | (Abnormality of head and neck );(Abnormality of the skeletal system ); | 2 | 1.45 |
| HP:0000975 | Hyperhidrosis | (Abnormality of the integument ); | 2 | 1.45 |
| HP:0002046 | Heat intolerance | (Abnormality of metabolism/homeostasis ); | 2 | 1.45 |
| HP:0012742 | Thin fingernail | (Abnormality of the integument ); | 2 | 1.45 |
| HP:0000561 | Absent eyelashes | (Abnormality of head and neck );(Abnormality of the integument ); | 2 | 1.45 |
| HP:0007392 | Excessive wrinkled skin | (Abnormality of the integument ); | 2 | 1.45 |
| HP:0010976 | B lymphocytopenia | (Abnormality of blood and blood-forming tissues );(Abnormality of the immune system ); | 2 | 1.45 |
| HP:0002217 | Slow-growing hair | (Abnormality of the integument ); | 2 | 1.45 |
| HP:0000716 | Depression | (Abnormality of the nervous system ); | 2 | 1.45 |
| HP:0002123 | Generalized myoclonic seizures | (Abnormality of the nervous system ); | 2 | 1.45 |
| HP:0002788 | Recurrent upper respiratory tract infections | (Abnormality of head and neck );(Abnormality of the respiratory system );(Abnormality of the immune system ); | 2 | 1.45 |
| HP:0002017 | Nausea and vomiting | (Abnormality of the digestive system ); | 2 | 1.45 |
| HP:0011127 | Perioral eczema | (Abnormality of the integument );(Abnormality of the immune system ); | 2 | 1.45 |
| HP:0002665 | Lymphoma | (Abnormality of blood and blood-forming tissues );(Neoplasm ); | 4 | 1.35 |
| HP:0002240 | Hepatomegaly | (Abnormality of the digestive system ); | 4 | 1.35 |
| HP:0001369 | Arthritis | (Abnormality of the skeletal system ); | 3 | 1.25 |
| HP:0004209 | Clinodactyly of the 5th finger | (Abnormality of the skeletal system );(Abnormality of limbs ); | 3 | 1.25 |
| HP:0001629 | Ventricular septal defect | (Abnormality of the cardiovascular system ); | 3 | 1.25 |
| HP:0100840 | aplasia/Hypoplasia of the eyebrow | (Abnormality of head and neck );(Abnormality of the integument ); | 3 | 1.25 |
| HP:0001324 | Muscle weakness | (Abnormality of the musculature ); | 3 | 1.25 |
| HP:0002015 | Dysphagia | (Abnormality of the nervous system );(Abnormality of the digestive system ); | 3 | 1.25 |
| HP:0011220 | Prominent forehead | (Abnormality of head and neck ); | 3 | 1.25 |
| HP:0000962 | Hyperkeratosis | (Abnormality of the integument ); | 3 | 1.25 |
| HP:0000217 | Xerostomia | (Abnormality of head and neck ); | 3 | 1.25 |
| HP:0002021 | Pyloric stenosis | (Abnormality of the digestive system ); | 3 | 1.2 |
| HP:0000768 | Pectus carinatum | (Abnormality of the skeletal system ); | 3 | 1.2 |
| HP:0100326 | Immunologic hypersensitivity | (Abnormality of the immune system ); | 3 | 1.2 |
| HP:0008069 | Neoplasm of the skin | (Abnormality of the integument );(Neoplasm ); | 3 | 1.2 |
| HP:0001163 | Abnormality of the metacarpal bones | (Abnormality of the skeletal system );(Abnormality of limbs ); | 3 | 1.2 |
| HP:0000969 | Edema | (Abnormality of metabolism/homeostasis ); | 5 | 1.15 |
| HP:0011675 | Arrhythmia | (Abnormality of the cardiovascular system ); | 2 | 1.1 |
| HP:0000275 | Narrow face | (Abnormality of head and neck ); | 2 | 1.1 |
| HP:0001773 | Short foot | (Abnormality of the skeletal system );(Abnormality of limbs ); | 2 | 1.1 |
| HP:0001852 | Sandal gap | (Abnormality of the skeletal system );(Abnormality of limbs ); | 2 | 1.1 |
| HP:0000348 | High forehead | (Abnormality of head and neck ); | 2 | 1.1 |
| HP:0001622 | Premature birth | (Abnormality of prenatal development or birth ); | 2 | 1.1 |
| HP:0010761 | Broad columella | (Abnormality of head and neck ); | 2 | 1.1 |
| HP:0001537 | Umbilical hernia | (Abnormality of connective tissue );(Abnormality of the digestive system ); | 2 | 1.1 |
| HP:0002014 | Diarrhea | (Abnormality of the digestive system ); | 2 | 1.1 |
| HP:0002027 | Abdominal pain | (Abnormality of the nervous system );(Abnormality of the digestive system ); | 2 | 1.1 |
| HP:0000490 | Deeply set eye | (Abnormality of head and neck );(Abnormality of the eye ); | 2 | 1.1 |
| HP:0012735 | Cough | (Abnormality of the respiratory system ); | 2 | 1.1 |
| HP:0000430 | Underdeveloped nasal alae | (Abnormality of head and neck ); | 2 | 1.1 |
| HP:0002553 | Highly arched eyebrow | (Abnormality of head and neck );(Abnormality of the integument ); | 2 | 1.1 |
| HP:0006610 | Wide intermamillary distance | (Abnormality of the breast ); | 2 | 1.1 |
| HP:0001386 | Joint swelling | (Abnormality of the skeletal system );(Abnormality of metabolism/homeostasis ); | 2 | 1.1 |
| HP:0002829 | Arthralgia | (Abnormality of the skeletal system ); | 2 | 1.1 |
| HP:0001268 | Mental deterioration | (Abnormality of the nervous system ); | 2 | 1.1 |
| HP:0000262 | Turricephaly | (Abnormality of head and neck );(Abnormality of the skeletal system ); | 2 | 1.1 |
| HP:0002110 | Bronchiectasis | (Abnormality of the respiratory system ); | 2 | 1.1 |
| HP:0001875 | Neutropenia | (Abnormality of blood and blood-forming tissues );(Abnormality of the immune system ); | 2 | 1.05 |
| HP:0002028 | Chronic diarrhea | (Abnormality of the digestive system ); | 2 | 1.05 |
| HP:0001874 | Abnormality of neutrophils | (Abnormality of blood and blood-forming tissues );(Abnormality of the immune system ); | 2 | 1.05 |
| HP:0003298 | Spina bifida occulta | (Abnormality of the nervous system ); | 2 | 1.05 |
| HP:0005528 | Bone marrow hypocellularity | (Abnormality of blood and blood-forming tissues ); | 2 | 1.05 |
| HP:0000592 | Blue sclerae | (Abnormality of the eye ); | 2 | 1.05 |
| HP:0009804 | Reduced number of teeth | (Abnormality of head and neck ); | 2 | 1.05 |
| HP:0002653 | Bone pain | (Abnormality of the nervous system );(Abnormality of the skeletal system ); | 2 | 1.05 |
| HP:0200037 | skin vesicle | (Abnormality of the integument ); | 2 | 1.05 |
| HP:0001019 | Erythroderma | (Abnormality of the integument );(Abnormality of the immune system ); | 2 | 1.05 |
| HP:0200039 | Pustule | (Abnormality of the integument );(Abnormality of the immune system ); | 2 | 1.05 |
| HP:0001047 | Atopic dermatitis | (Abnormality of the integument );(Abnormality of the immune system ); | 2 | 1.05 |
| HP:0002754 | Osteomyelitis | (Abnormality of the skeletal system );(Abnormality of the immune system ); | 2 | 1.05 |
| HP:0000956 | Acanthosis nigricans | (Abnormality of the integument ); | 2 | 1.05 |
| HP:0011800 | Midface retrusion | (Abnormality of head and neck ); | 2 | 1.05 |
| HP:0004097 | Deviation of finger | (Abnormality of the skeletal system );(Abnormality of limbs ); | 2 | 1.05 |
| HP:0000483 | Astigmatism | (Abnormality of the eye ); | 2 | 1.05 |
| HP:0000613 | Photophobia | (Abnormality of the eye );(Abnormality of the nervous system ); | 2 | 1.05 |
| HP:0100825 | Cheilitis | (Abnormality of head and neck ); | 2 | 1.05 |
| HP:0001051 | Seborrheic dermatitis | (Abnormality of the integument );(Abnormality of the immune system ); | 2 | 1.05 |
| HP:0005930 | Abnormality of the epiphyses | (Abnormality of the skeletal system ); | 2 | 1.05 |
| HP:0008736 | Hypoplasia of penis | (Abnormality of the genitourinary system ); | 2 | 1.05 |
| HP:0002231 | Sparse body hair | (Abnormality of the integument ); | 2 | 1.05 |
| HP:0100679 | Lack of skin elasticity | (Abnormality of the integument ); | 2 | 1.05 |
| HP:0001097 | Keratoconjunctivitis sicca | (Abnormality of head and neck );(Abnormality of the eye );(Abnormality of the immune system ); | 2 | 1.05 |
| HP:0002079 | Hypoplasia of the corpus callosum | (Abnormality of the nervous system ); | 2 | 1.05 |
| HP:0010306 | Short thorax | (Abnormality of the skeletal system ); | 2 | 1.05 |
| HP:0002664 | Neoplasm | (Neoplasm ); | 4 | 1 |
| HP:0001363 | Craniosynostosis | (Abnormality of head and neck );(Abnormality of the skeletal system ); | 4 | 1 |
| HP:0100490 | Camptodactyly of finger | (Abnormality of the musculature );(Abnormality of limbs );(Abnormality of connective tissue );(Abnormality of the skeletal system ); | 4 | 1 |
| HP:0000968 | Ectodermal dysplasia | (Abnormality of the integument ); | 1 | 0.9999 |
| HP:0000973 | Cutis laxa | (Abnormality of the integument ); | 1 | 0.9999 |
| HP:0003010 | Prolonged bleeding time | (Abnormality of blood and blood-forming tissues ); | 1 | 0.9 |
| HP:0007420 | Spontaneous hematomas | (Abnormality of the integument );(Abnormality of the cardiovascular system );(Abnormality of blood and blood-forming tissues ); | 1 | 0.9 |
| HP:0011029 | Internal hemorrhage | (Abnormality of the cardiovascular system );(Abnormality of blood and blood-forming tissues ); | 1 | 0.9 |
| HP:0011875 | Abnormal platelet morphology | (Abnormality of blood and blood-forming tissues ); | 1 | 0.9 |
| HP:0001034 | Hypermelanotic macule | (Abnormality of the integument ); | 1 | 0.9 |
| HP:0002575 | Tracheoesophageal fistula | (Abnormality of the respiratory system );(Abnormality of the digestive system ); | 1 | 0.9 |
| HP:0100721 | Mediastinal lymphadenopathy | (Abnormality of the immune system );(Abnormality of the thoracic cavity ); | 1 | 0.9 |
| HP:0009601 | aplasia/Hypoplasia of the thumb | (Abnormality of the skeletal system );(Abnormality of limbs ); | 1 | 0.9 |
| HP:0009602 | Abnormality of thumb phalanx | (Abnormality of the skeletal system );(Abnormality of limbs ); | 1 | 0.9 |
| HP:0009891 | Underdeveloped supraorbital ridges | (Abnormality of head and neck ); | 1 | 0.9 |
| HP:0011304 | Broad thumb | (Abnormality of the skeletal system );(Abnormality of limbs ); | 1 | 0.9 |
| HP:0008872 | Feeding difficulties in infancy | (Abnormality of the digestive system ); | 1 | 0.9 |
| HP:0000157 | Abnormality of the tongue | (Abnormality of head and neck ); | 1 | 0.9 |
| HP:0000168 | Abnormality of the gingiva | (Abnormality of head and neck ); | 1 | 0.9 |
| HP:0000501 | Glaucoma | (Abnormality of the eye ); | 1 | 0.9 |
| HP:0000822 | Hypertension | (Abnormality of the cardiovascular system ); | 1 | 0.9 |
| HP:0000974 | Hyperextensible skin | (Abnormality of the integument ); | 1 | 0.9 |
| HP:0000987 | Atypical scarring of skin | (Abnormality of the integument );(Abnormality of connective tissue ); | 1 | 0.9 |
| HP:0001065 | Striae distensae | (Abnormality of the integument ); | 1 | 0.9 |
| HP:0001166 | Arachnodactyly | (Abnormality of the skeletal system );(Abnormality of limbs ); | 1 | 0.9 |
| HP:0003307 | Hyperlordosis | (Abnormality of the skeletal system ); | 1 | 0.9 |
| HP:0005105 | Abnormal nasal morphology | (Abnormality of head and neck ); | 1 | 0.9 |
| HP:0005294 | Arterial dissection | (Abnormality of the cardiovascular system ); | 1 | 0.9 |
| HP:0009721 | Shagreen patch | (Abnormality of the integument );(Abnormality of connective tissue ); | 1 | 0.9 |
| HP:0010620 | Malar prominence | (Abnormality of head and neck );(Abnormality of the skeletal system ); | 1 | 0.9 |
| HP:0011830 | Abnormality of oral mucosa | (Abnormality of head and neck ); | 1 | 0.9 |
| HP:0011354 | Generalized abnormality of skin | (Abnormality of the integument ); | 1 | 0.9 |
| HP:0100750 | Atelectasis | (Abnormality of the respiratory system ); | 1 | 0.9 |
| HP:0007479 | Congenital nonbullous ichthyosiform erythroderma | (Abnormality of the integument ); | 1 | 0.9 |
| HP:0001070 | Mottled pigmentation | (Abnormality of the integument ); | 1 | 0.9 |
| HP:0000496 | Abnormality of eye movement | (Abnormality of the eye ); | 1 | 0.9 |
| HP:0001315 | Reduced tendon reflexes | (Abnormality of the nervous system ); | 1 | 0.9 |
| HP:0001338 | Partial agenesis of the corpus callosum | (Abnormality of the nervous system ); | 1 | 0.9 |
| HP:0007559 | Localized epidermolytic hyperkeratosis | (Abnormality of the integument ); | 1 | 0.9 |
| HP:0001608 | Abnormality of the voice | (Abnormality of the voice ); | 1 | 0.9 |
| HP:0010468 | aplasia/Hypoplasia of the testes | (Abnormality of the genitourinary system ); | 1 | 0.9 |
| HP:0100585 | Teleangiectasia of the skin | (Abnormality of the integument );(Abnormality of the cardiovascular system ); | 1 | 0.9 |
| HP:0002300 | Mutism | (Abnormality of the nervous system ); | 1 | 0.9 |
| HP:0002357 | Dysphasia | (Abnormality of the nervous system ); | 1 | 0.9 |
| HP:0002381 | Aphasia | (Abnormality of the nervous system ); | 1 | 0.9 |
| HP:0009928 | Thick nasal alae | (Abnormality of head and neck ); | 1 | 0.9 |
| HP:0010529 | Echolalia | (Abnormality of the nervous system ); | 1 | 0.9 |
| HP:0000951 | Abnormality of the skin | (Abnormality of the integument ); | 1 | 0.9 |
| HP:0001582 | Redundant skin | (Abnormality of the integument ); | 1 | 0.9 |
| HP:0001643 | Patent ductus arteriosus | (Abnormality of the cardiovascular system ); | 1 | 0.9 |
| HP:0005222 | Bowel diverticulosis | (Abnormality of the digestive system ); | 1 | 0.9 |
| HP:0007495 | Prematurely aged appearance | (Abnormality of the integument ); | 1 | 0.9 |
| HP:0007703 | Abnormal retinal pigmentation | (Abnormality of the eye ); | 1 | 0.9 |
| HP:0100628 | Esophageal diverticulum | (Abnormality of the digestive system ); | 1 | 0.9 |
| HP:0004332 | Abnormality of lymphocytes | (Abnormality of blood and blood-forming tissues );(Abnormality of the immune system ); | 1 | 0.9 |
| HP:0200035 | Skin plaque | (Abnormality of the integument ); | 1 | 0.9 |
| HP:0002090 | Pneumonia | (Abnormality of the respiratory system );(Abnormality of the immune system ); | 1 | 0.9 |
| HP:0004429 | Recurrent viral infections | (Abnormality of the immune system ); | 1 | 0.9 |
| HP:0005401 | Recurrent candida infections | (Abnormality of the immune system ); | 1 | 0.9 |
| HP:0005403 | T lymphocytopenia | (Abnormality of blood and blood-forming tissues );(Abnormality of the immune system ); | 2 | 0.9 |
| HP:0005406 | Recurrent bacterial skin infections | (Abnormality of the integument );(Abnormality of the immune system ); | 1 | 0.9 |
| HP:0011108 | Recurrent sinusitis | (Abnormality of the skeletal system );(Abnormality of the respiratory system );(Abnormality of head and neck );(Abnormality of the immune system ); | 1 | 0.9 |
| HP:0012203 | Onychomycosis | (Abnormality of the immune system ); | 1 | 0.9 |
| HP:0200036 | Skin nodule | (Abnormality of the integument ); | 1 | 0.9 |
| HP:0100678 | Premature skin wrinkling | (Abnormality of the integument ); | 1 | 0.9 |
| HP:0000327 | Hypoplasia of the maxilla | (Abnormality of head and neck );(Abnormality of the skeletal system ); | 1 | 0.9 |
| HP:0010978 | Abnormality of immune system physiology | (Abnormality of the immune system ); | 1 | 0.9 |
| HP:0000158 | Macroglossia | (Abnormality of head and neck );(Abnormality of the musculature ); | 1 | 0.9 |
| HP:0000577 | Exotropia | (Abnormality of the eye ); | 1 | 0.9 |
| HP:0000629 | Periorbital fullness | (Abnormality of head and neck ); | 1 | 0.9 |
| HP:0000646 | Amblyopia | (Abnormality of the eye ); | 1 | 0.9 |
| HP:0000805 | Enuresis | (Abnormality of the genitourinary system ); | 1 | 0.9 |
| HP:0001269 | Hemiparesis | (Abnormality of the nervous system ); | 1 | 0.9 |
| HP:0001716 | Wolff-Parkinson-White syndrome | (Abnormality of the cardiovascular system ); | 1 | 0.9 |
| HP:0002136 | Broad-based gait | (Abnormality of the nervous system ); | 1 | 0.9 |
| HP:0012680 | Abnormality of the pineal gland | (Abnormality of the endocrine system ); | 1 | 0.9 |
| HP:0100703 | Tongue thrusting | (Abnormality of the nervous system ); | 1 | 0.9 |
| HP:0001738 | Exocrine pancreatic insufficiency | (Abnormality of the digestive system ); | 1 | 0.9 |
| HP:0000712 | Emotional lability | (Abnormality of the nervous system ); | 1 | 0.9 |
| HP:0000739 | Anxiety | (Abnormality of the nervous system ); | 1 | 0.9 |
| HP:0002150 | Hypercalciuria | (Abnormality of the genitourinary system );(Abnormality of metabolism/homeostasis ); | 1 | 0.9 |
| HP:0002356 | Writer's cramp | (Abnormality of the nervous system ); | 1 | 0.9 |
| HP:0002901 | Hypocalcemia | (Abnormality of metabolism/homeostasis ); | 1 | 0.9 |
| HP:0003401 | Paresthesia | (Abnormality of the nervous system ); | 1 | 0.9 |
| HP:0003457 | EMG abnormality | (Abnormality of the musculature ); | 1 | 0.9 |
| HP:0003473 | Fatigable weakness | (Abnormality of the nervous system );(Abnormality of the musculature ); | 1 | 0.9 |
| HP:0040148 | ##### | (Abnormality of the nervous system ); | 1 | 0.9 |
| HP:0000293 | Full cheeks | (Abnormality of head and neck ); | 1 | 0.9 |
| HP:0000322 | Short philtrum | (Abnormality of head and neck ); | 1 | 0.9 |
| HP:0002263 | Exaggerated cupid's bow | (Abnormality of head and neck ); | 1 | 0.9 |
| HP:0000543 | Optic disc pallor | (Abnormality of the eye ); | 1 | 0.9 |
| HP:0000649 | Abnormality of vision evoked potentials | (Abnormality of the eye ); | 1 | 0.9 |
| HP:0000713 | Agitation | (Abnormality of the nervous system ); | 1 | 0.9 |
| HP:0000729 | Autistic behavior | (Abnormality of the nervous system ); | 1 | 0.9 |
| HP:0010554 | Cutaneous finger syndactyly | (Abnormality of the skeletal system );(Abnormality of limbs ); | 1 | 0.9 |
| HP:0002521 | Hypsarrhythmia | (Abnormality of the nervous system ); | 1 | 0.9 |
| HP:0003236 | Elevated serum creatine phosphokinase | (Abnormality of metabolism/homeostasis ); | 1 | 0.9 |
| HP:0003282 | Low alkaline phosphatase | (Abnormality of metabolism/homeostasis ); | 1 | 0.9 |
| HP:0003700 | Generalized amyotrophy | (Abnormality of the musculature ); | 1 | 0.9 |
| HP:0004691 | 2-3 toe syndactyly | (Abnormality of the skeletal system );(Abnormality of limbs ); | 1 | 0.9 |
| HP:0001054 | Numerous nevi | (Abnormality of the integument );(Neoplasm ); | 1 | 0.9 |
| HP:0007328 | Impaired pain sensation | (Abnormality of the nervous system ); | 1 | 0.9 |
| HP:0009908 | Anterior creases of earlobe | (Abnormality of the ear ); | 1 | 0.9 |
| HP:0011265 | Cleft earlobe | (Abnormality of the ear ); | 1 | 0.9 |
| HP:0011343 | Moderate global developmental delay | (Abnormality of the nervous system ); | 1 | 0.9 |
| HP:0012751 | Abnormal basal ganglia MRI signal intensity | (Abnormality of the nervous system ); | 1 | 0.9 |
| HP:0030353 | Decreased serum insulin-like growth factor 1 | (Abnormality of the endocrine system ); | 1 | 0.9 |
| HP:0100739 | Bulimia | (Abnormality of the nervous system ); | 1 | 0.9 |
| HP:0000668 | Hypodontia | (Abnormality of head and neck ); | 1 | 0.9 |
| HP:0000009 | Functional abnormality of the bladder | (Abnormality of the genitourinary system ); | 1 | 0.9 |
| HP:0002242 | Abnormality of the intestine | (Abnormality of the digestive system ); | 1 | 0.9 |
| HP:0002728 | Chronic mucocutaneous candidiasis | (Abnormality of the integument );(Abnormality of the immune system ); | 1 | 0.9 |
| HP:0000819 | Diabetes mellitus | (Abnormality of the endocrine system );(Abnormality of metabolism/homeostasis ); | 1 | 0.9 |
| HP:0008065 | aplasia/Hypoplasia of the skin | (Abnormality of the integument ); | 1 | 0.9 |
| HP:0002293 | Alopecia of scalp | (Abnormality of head and neck );(Abnormality of the integument ); | 1 | 0.9 |
| HP:0100643 | Abnormality of nail color | (Abnormality of the integument ); | 1 | 0.9 |
| HP:0001010 | Hypopigmentation of the skin | (Abnormality of the integument ); | 1 | 0.9 |
| HP:0004923 | Hyperphenylalaninemia | (Abnormality of metabolism/homeostasis ); | 1 | 0.9 |
| HP:0005599 | Hypopigmentation of hair | (Abnormality of the integument ); | 1 | 0.9 |
| HP:0010864 | Intellectual disability, severe | (Abnormality of the nervous system ); | 1 | 0.9 |
| HP:0005979 | Metabolic ketoacidosis | (Abnormality of metabolism/homeostasis ); | 1 | 0.9 |
| HP:0000737 | Irritability | (Abnormality of the nervous system ); | 1 | 0.9 |
| HP:0002039 | Anorexia | (Abnormality of the digestive system ); | 1 | 0.9 |
| HP:0010472 | Abnormality of the heme biosynthetic pathway | (Abnormality of metabolism/homeostasis ); | 1 | 0.9 |
| HP:0000248 | Brachycephaly | (Abnormality of head and neck );(Abnormality of the skeletal system ); | 1 | 0.9 |
| HP:0000889 | Abnormality of the clavicles | (Abnormality of the skeletal system ); | 1 | 0.9 |
| HP:0002023 | Anal atresia | (Abnormality of the digestive system ); | 1 | 0.9 |
| HP:0002697 | Parietal foramina | (Abnormality of head and neck );(Abnormality of the skeletal system ); | 1 | 0.9 |
| HP:0004440 | Coronal craniosynostosis | (Abnormality of head and neck );(Abnormality of the skeletal system ); | 1 | 0.9 |
| HP:0004491 | Large posterior fontanelle | (Abnormality of head and neck );(Abnormality of the skeletal system ); | 1 | 0.9 |
| HP:0006660 | aplastic clavicles | (Abnormality of the skeletal system ); | 1 | 0.9 |
| HP:0008368 | Tarsal synostosis | (Abnormality of the skeletal system );(Abnormality of limbs ); | 1 | 0.9 |
| HP:0100589 | Urogenital fistula | (Abnormality of the genitourinary system ); | 1 | 0.9 |
| HP:0200044 | Porokeratosis | (Abnormality of the integument ); | 1 | 0.9 |
| HP:0030680 | Abnormality of cardiovascular system morphology | (Abnormality of the cardiovascular system ); | 3 | 0.85 |
| HP:0000431 | Wide nasal bridge | (Abnormality of head and neck ); | 3 | 0.85 |
| HP:0001763 | Pes planus | (Abnormality of limbs ); | 3 | 0.85 |
| HP:0001155 | Abnormality of the hand | (Abnormality of limbs ); | 3 | 0.85 |
| HP:0000518 | Cataract | (Abnormality of the eye ); | 5 | 0.75 |
| HP:0000225 | Gingival bleeding | (Abnormality of head and neck );(Abnormality of blood and blood-forming tissues ); | 2 | 0.7 |
| HP:0002573 | Hematochezia | (Abnormality of the cardiovascular system );(Abnormality of blood and blood-forming tissues );(Abnormality of the digestive system ); | 2 | 0.7 |
| HP:0012378 | Fatigue | (Abnormality of the nervous system ); | 2 | 0.7 |
| HP:0100533 | Inflammatory abnormality of the eye | (Abnormality of the eye );(Abnormality of the immune system ); | 2 | 0.7 |
| HP:0000238 | Hydrocephalus | (Abnormality of the nervous system ); | 2 | 0.7 |
| HP:0002093 | Respiratory insufficiency | (Abnormality of the respiratory system ); | 2 | 0.7 |
| HP:0000482 | Microcornea | (Abnormality of the eye ); | 2 | 0.7 |
| HP:0002758 | Osteoarthritis | (Abnormality of the skeletal system ); | 2 | 0.7 |
| HP:0100823 | Genital hernia | (Abnormality of connective tissue ); | 2 | 0.7 |
| HP:0001974 | Leukocytosis | (Abnormality of blood and blood-forming tissues );(Abnormality of the immune system ); | 2 | 0.7 |
| HP:0011024 | Abnormality of the gastrointestinal tract | (Abnormality of the digestive system ); | 2 | 0.7 |
| HP:0000271 | Abnormality of the face | (Abnormality of head and neck ); | 2 | 0.7 |
| HP:0000535 | Sparse eyebrow | (Abnormality of head and neck );(Abnormality of the integument ); | 2 | 0.7 |
| HP:0000717 | Autism | (Abnormality of the nervous system ); | 2 | 0.7 |
| HP:0000400 | Macrotia | (Abnormality of the ear ); | 2 | 0.7 |
| HP:0200020 | Corneal erosion | (Abnormality of the eye ); | 2 | 0.7 |
| HP:0000072 | Hydroureter | (Abnormality of the genitourinary system ); | 2 | 0.7 |
| HP:0003510 | Severe short stature | (Growth abnormality ); | 2 | 0.7 |
| HP:0007957 | Corneal opacity | (Abnormality of the eye ); | 2 | 0.7 |
| HP:0000280 | Coarse facial features | (Abnormality of head and neck ); | 2 | 0.7 |
| HP:0010720 | Abnormal hair pattern | (Abnormality of the integument ); | 2 | 0.7 |
| HP:0011362 | Abnormal hair quantity | (Abnormality of the integument ); | 2 | 0.7 |
| HP:0003272 | Abnormality of the hip bone | (Abnormality of the skeletal system ); | 2 | 0.7 |
| HP:0100790 | Hernia | (Abnormality of connective tissue ); | 2 | 0.7 |
| HP:0000457 | Depressed nasal ridge | (Abnormality of head and neck ); | 2 | 0.7 |
| HP:0001357 | Plagiocephaly | (Abnormality of head and neck );(Abnormality of the skeletal system ); | 2 | 0.7 |
| HP:0000100 | Nephrotic syndrome | (Abnormality of the genitourinary system ); | 2 | 0.7 |
| HP:0100651 | Type I diabetes mellitus | (Abnormality of the endocrine system );(Abnormality of metabolism/homeostasis ); | 2 | 0.7 |
| HP:0000648 | Optic atrophy | (Abnormality of the eye ); | 2 | 0.7 |
| HP:0000445 | Wide nose | (Abnormality of head and neck ); | 2 | 0.7 |
| HP:0001581 | Recurrent skin infections | (Abnormality of the integument );(Abnormality of the immune system ); | 2 | 0.7 |
| HP:0001276 | Hypertonia | (Abnormality of the nervous system );(Abnormality of the musculature ); | 2 | 0.7 |
| HP:0001251 | Ataxia | (Abnormality of the nervous system ); | 2 | 0.7 |
| HP:0007549 | Desquamation of skin soon after birth | (Abnormality of the integument ); | 2 | 0.7 |
| HP:0000126 | Hydronephrosis | (Abnormality of the genitourinary system ); | 4 | 0.6 |
| HP:0000545 | Myopia | (Abnormality of the eye ); | 4 | 0.6 |
| HP:0001635 | Congestive heart failure | (Abnormality of the cardiovascular system ); | 4 | 0.6 |
| HP:0000979 | Purpura | (Abnormality of the integument );(Abnormality of the cardiovascular system );(Abnormality of blood and blood-forming tissues ); | 1 | 0.55 |
| HP:0001878 | Hemolytic anemia | (Abnormality of blood and blood-forming tissues ); | 1 | 0.55 |
| HP:0001879 | Abnormality of eosinophils | (Abnormality of blood and blood-forming tissues );(Abnormality of the immune system ); | 1 | 0.55 |
| HP:0002037 | Inflammation of the large intestine | (Abnormality of the immune system );(Abnormality of the digestive system ); | 1 | 0.55 |
| HP:0002094 | Dyspnea | (Abnormality of the respiratory system ); | 1 | 0.55 |
| HP:0002248 | Hematemesis | (Abnormality of the cardiovascular system );(Abnormality of blood and blood-forming tissues );(Abnormality of the digestive system ); | 1 | 0.55 |
| HP:0000055 | Abnormality of female external genitalia | (Abnormality of the genitourinary system ); | 1 | 0.55 |
| HP:0000176 | Submucous cleft hard palate | (Abnormality of head and neck ); | 1 | 0.55 |
| HP:0000294 | Low anterior hairline | (Abnormality of head and neck );(Abnormality of the integument ); | 1 | 0.55 |
| HP:0000340 | Sloping forehead | (Abnormality of head and neck ); | 1 | 0.55 |
| HP:0000411 | Protruding ear | (Abnormality of the ear ); | 1 | 0.55 |
| HP:0005338 | Sparse lateral eyebrow | (Abnormality of head and neck );(Abnormality of the integument ); | 1 | 0.55 |
| HP:0008897 | Postnatal growth retardation | (Growth abnormality ); | 1 | 0.55 |
| HP:0009738 | Abnormality of the antihelix | (Abnormality of the ear ); | 1 | 0.55 |
| HP:0000324 | Facial asymmetry | (Abnormality of head and neck ); | 1 | 0.55 |
| HP:0000921 | Missing ribs | (Abnormality of the skeletal system ); | 1 | 0.55 |
| HP:0001831 | Short toe | (Abnormality of the skeletal system );(Abnormality of limbs ); | 1 | 0.55 |
| HP:0001847 | Long hallux | (Abnormality of the skeletal system );(Abnormality of limbs ); | 1 | 0.55 |
| HP:0001863 | Toe clinodactyly | (Abnormality of the skeletal system );(Abnormality of limbs ); | 1 | 0.55 |
| HP:0003312 | Abnormal form of the vertebral bodies | (Abnormality of the skeletal system ); | 1 | 0.55 |
| HP:0009906 | aplasia/Hypoplasia of the earlobes | (Abnormality of the ear ); | 1 | 0.55 |
| HP:0010059 | Broad hallux phalanx | (Abnormality of the skeletal system );(Abnormality of limbs ); | 1 | 0.55 |
| HP:0001063 | Acrocyanosis | (Abnormality of the integument );(Abnormality of the respiratory system ); | 1 | 0.55 |
| HP:0001704 | Tricuspid valve prolapse | (Abnormality of the cardiovascular system ); | 1 | 0.55 |
| HP:0002036 | Hiatus hernia | (Abnormality of connective tissue );(Abnormality of the digestive system ); | 1 | 0.55 |
| HP:0003326 | Myalgia | (Abnormality of the musculature ); | 1 | 0.55 |
| HP:0005293 | Venous insufficiency | (Abnormality of the cardiovascular system ); | 1 | 0.55 |
| HP:0100720 | Hypoplasia of the ear cartilage | (Abnormality of the ear ); | 1 | 0.55 |
| HP:0008392 | Subungual hyperkeratosis | (Abnormality of the integument ); | 1 | 0.55 |
| HP:0002013 | Vomiting | (Abnormality of the digestive system ); | 1 | 0.55 |
| HP:0002570 | Steatorrhea | (Abnormality of the digestive system ); | 1 | 0.55 |
| HP:0003073 | Hypoalbuminemia | (Abnormality of metabolism/homeostasis ); | 1 | 0.55 |
| HP:0003193 | Allergic rhinitis | (Abnormality of head and neck );(Abnormality of the immune system ); | 1 | 0.55 |
| HP:0011227 | Elevated C-reactive protein level | (Abnormality of metabolism/homeostasis ); | 1 | 0.55 |
| HP:0000118 | Phenotypic abnormality |  | 1 | 0.55 |
| HP:0001818 | Paronychia | (Abnormality of the integument ); | 1 | 0.55 |
| HP:0004313 | Hypogammaglobulinemia | (Abnormality of blood and blood-forming tissues );(Abnormality of metabolism/homeostasis );(Abnormality of the immune system ); | 1 | 0.55 |
| HP:0000582 | Upslanted palpebral fissure | (Abnormality of head and neck ); | 1 | 0.55 |
| HP:0002558 | Supernumerary nipples | (Abnormality of the breast ); | 1 | 0.55 |
| HP:0007598 | Bilateral single transverse palmar creases | (Abnormality of the integument );(Abnormality of limbs ); | 1 | 0.55 |
| HP:0010049 | Short metacarpal | (Abnormality of the skeletal system );(Abnormality of limbs ); | 1 | 0.55 |
| HP:0001387 | Joint stiffness | (Abnormality of the skeletal system ); | 1 | 0.55 |
| HP:0002815 | Abnormality of the knees | (Abnormality of the skeletal system );(Abnormality of limbs ); | 1 | 0.55 |
| HP:0002992 | Abnormality of the tibia | (Abnormality of the skeletal system );(Abnormality of limbs ); | 1 | 0.55 |
| HP:0000499 | Abnormality of the eyelashes | (Abnormality of head and neck );(Abnormality of the integument ); | 1 | 0.55 |
| HP:0000614 | Abnormality of the nasolacrimal system | (Abnormality of head and neck ); | 1 | 0.55 |
| HP:0000726 | Dementia | (Abnormality of the nervous system ); | 1 | 0.55 |
| HP:0001804 | Hypoplastic fingernail | (Abnormality of the integument ); | 1 | 0.55 |
| HP:0001812 | Hyperconvex fingernails | (Abnormality of the integument ); | 1 | 0.55 |
| HP:0002376 | Developmental regression | (Abnormality of the nervous system ); | 1 | 0.55 |
| HP:0002718 | Recurrent bacterial infections | (Abnormality of the immune system ); | 1 | 0.55 |
| HP:0004370 | Abnormality of temperature regulation | (Abnormality of metabolism/homeostasis ); | 1 | 0.55 |
| HP:0011968 | Feeding difficulties | (Abnormality of the digestive system ); | 1 | 0.55 |
| HP:0045074 | Thin eyebrow | (Abnormality of head and neck );(Abnormality of the integument ); | 1 | 0.55 |
| HP:0001061 | Acne | (Abnormality of the integument );(Abnormality of the immune system ); | 1 | 0.55 |
| HP:0001376 | Limitation of joint mobility | (Abnormality of the skeletal system ); | 1 | 0.55 |
| HP:0002797 | Osteolysis | (Abnormality of the skeletal system ); | 1 | 0.55 |
| HP:0010541 | Cutis gyrata of scalp | (Abnormality of head and neck );(Abnormality of the integument ); | 1 | 0.55 |
| HP:0008499 | High-grade hypermetropia | (Abnormality of the eye ); | 1 | 0.55 |
| HP:0012393 | Allergy | (Abnormality of the immune system ); | 1 | 0.55 |
| HP:0040189 | Scaling skin | (Abnormality of the integument ); | 1 | 0.55 |
| HP:0000377 | Abnormality of the pinna | (Abnormality of the ear ); | 1 | 0.55 |
| HP:0002289 | Alopecia universalis | (Abnormality of the integument ); | 1 | 0.55 |
| HP:0002299 | Brittle hair | (Abnormality of the integument ); | 1 | 0.55 |
| HP:0002552 | Trichodysplasia | (Abnormality of the integument ); | 1 | 0.55 |
| HP:0007439 | Generalized keratosis follicularis | (Abnormality of the integument ); | 1 | 0.55 |
| HP:0008394 | Congenital onychodystrophy | (Abnormality of the integument ); | 1 | 0.55 |
| HP:0010562 | Keloids | (Abnormality of the integument );(Abnormality of connective tissue ); | 1 | 0.55 |
| HP:0012725 | Cutaneous syndactyly | (Abnormality of the skeletal system );(Abnormality of limbs ); | 1 | 0.55 |
| HP:0030953 | Conjunctival hyperemia | (Abnormality of head and neck );(Abnormality of the eye );(Abnormality of the cardiovascular system ); | 1 | 0.55 |
| HP:0410030 | Cleft lip | (Abnormality of head and neck ); | 1 | 0.55 |
| HP:0000972 | Palmoplantar hyperkeratosis | (Abnormality of the integument );(Abnormality of limbs ); | 1 | 0.55 |
| HP:0002745 | Oral leukoplakia | (Abnormality of head and neck ); | 1 | 0.55 |
| HP:0010296 | Ankyloglossia | (Abnormality of head and neck ); | 1 | 0.55 |
| HP:0100587 | Abnormality of the preputium | (Abnormality of the genitourinary system ); | 1 | 0.55 |
| HP:0100669 | Abnormal pigmentation of the oral mucosa | (Abnormality of head and neck ); | 1 | 0.55 |
| HP:0000035 | Abnormality of the testis | (Abnormality of the genitourinary system ); | 1 | 0.55 |
| HP:0000446 | Narrow nasal bridge | (Abnormality of head and neck ); | 1 | 0.55 |
| HP:0000527 | Long eyelashes | (Abnormality of head and neck );(Abnormality of the integument ); | 1 | 0.55 |
| HP:0001167 | Abnormality of finger | (Abnormality of the skeletal system );(Abnormality of limbs ); | 1 | 0.55 |
| HP:0002121 | Absence seizures | (Abnormality of the nervous system ); | 1 | 0.55 |
| HP:0002133 | Status epilepticus | (Abnormality of the nervous system ); | 1 | 0.55 |
| HP:0007665 | Curly eyelashes | (Abnormality of head and neck );(Abnormality of the integument ); | 1 | 0.55 |
| HP:0009836 | Broad distal phalanx of finger | (Abnormality of the skeletal system );(Abnormality of limbs ); | 1 | 0.55 |
| HP:0011097 | Epileptic spasms | (Abnormality of the nervous system ); | 1 | 0.55 |
| HP:0012745 | Short palpebral fissure | (Abnormality of head and neck ); | 1 | 0.55 |
| HP:0000010 | Recurrent urinary tract infections | (Abnormality of the genitourinary system );(Abnormality of the immune system ); | 1 | 0.55 |
| HP:0000076 | Vesicoureteral reflux | (Abnormality of the genitourinary system ); | 1 | 0.55 |
| HP:0000366 | Abnormality of the nose | (Abnormality of head and neck ); | 1 | 0.55 |
| HP:0000474 | Thickened nuchal skin fold | (Abnormality of head and neck );(Abnormality of prenatal development or birth ); | 1 | 0.55 |
| HP:0002607 | Bowel incontinence | (Abnormality of the digestive system ); | 1 | 0.55 |
| HP:0010295 | aplasia/Hypoplasia of the tongue | (Abnormality of head and neck ); | 1 | 0.55 |
| HP:0001029 | Poikiloderma | (Abnormality of the integument ); | 1 | 0.55 |
| HP:0002716 | Lymphadenopathy | (Abnormality of the immune system ); | 1 | 0.55 |
| HP:0012192 | Cutaneous T-cell lymphoma | (Abnormality of blood and blood-forming tissues );(Neoplasm ); | 1 | 0.55 |
| HP:0100963 | Hyperesthesia | (Abnormality of the nervous system ); | 1 | 0.55 |
| HP:0004298 | Abnormality of the abdominal wall | (Abnormality of the digestive system ); | 1 | 0.55 |
| HP:0011358 | Generalized hypopigmentation of hair | (Abnormality of the integument ); | 1 | 0.55 |
| HP:0000944 | Abnormality of the metaphyses | (Abnormality of the skeletal system );(Abnormality of limbs ); | 1 | 0.55 |
| HP:0000121 | Nephrocalcinosis | (Abnormality of the genitourinary system ); | 1 | 0.55 |
| HP:0002793 | Abnormal pattern of respiration | (Abnormality of the respiratory system ); | 1 | 0.55 |
| HP:0002905 | Hyperphosphatemia | (Abnormality of metabolism/homeostasis ); | 1 | 0.55 |
| HP:0002917 | Hypomagnesemia | (Abnormality of metabolism/homeostasis ); | 1 | 0.55 |
| HP:0012608 | Hypermagnesiuria | (Abnormality of the genitourinary system );(Abnormality of metabolism/homeostasis ); | 1 | 0.55 |
| HP:0000647 | Sclerocornea | (Abnormality of the eye ); | 1 | 0.55 |
| HP:0001611 | Nasal speech | (Abnormality of the voice ); | 1 | 0.55 |
| HP:0010489 | Absent palmar crease | (Abnormality of the integument );(Abnormality of limbs ); | 1 | 0.55 |
| HP:0000818 | Abnormality of the endocrine system | (Abnormality of the endocrine system ); | 1 | 0.55 |
| HP:0000823 | Delayed puberty | (Abnormality of the endocrine system );(Growth abnormality ); | 1 | 0.55 |
| HP:0000832 | Primary hypothyroidism | (Abnormality of the endocrine system ); | 1 | 0.55 |
| HP:0001433 | Hepatosplenomegaly | (Abnormality of the immune system );(Abnormality of the digestive system ); | 1 | 0.55 |
| HP:0001890 | Autoimmune hemolytic anemia | (Abnormality of blood and blood-forming tissues );(Abnormality of the immune system ); | 1 | 0.55 |
| HP:0001920 | Renal artery stenosis | (Abnormality of the genitourinary system );(Abnormality of the cardiovascular system ); | 1 | 0.55 |
| HP:0002958 | Immune dysregulation | (Abnormality of the immune system ); | 1 | 0.55 |
| HP:0004387 | Enterocolitis | (Abnormality of the immune system );(Abnormality of the digestive system ); | 1 | 0.55 |
| HP:0004944 | Cerebral aneurysm | (Abnormality of the nervous system );(Abnormality of the cardiovascular system ); | 1 | 0.55 |
| HP:0005353 | Susceptibility to herpesvirus | (Abnormality of the immune system ); | 1 | 0.55 |
| HP:0011123 | Inflammatory abnormality of the skin | (Abnormality of the integument );(Abnormality of the immune system ); | 1 | 0.55 |
| HP:0011473 | Villous atrophy | (Abnormality of the digestive system ); | 1 | 0.55 |
| HP:0012163 | Carotid artery aneurysm | (Abnormality of the cardiovascular system ); | 1 | 0.55 |
| HP:0040160 | ##### | (Abnormality of the skeletal system ); | 1 | 0.55 |
| HP:0100646 | Thyroiditis | (Abnormality of the endocrine system );(Abnormality of the immune system ); | 1 | 0.55 |
| HP:0100817 | Renovascular hypertension | (Abnormality of the genitourinary system );(Abnormality of the cardiovascular system ); | 1 | 0.55 |
| HP:0003765 | Psoriasis | (Abnormality of the integument );(Abnormality of the immune system ); | 1 | 0.55 |
| HP:0000221 | Furrowed tongue | (Abnormality of head and neck ); | 1 | 0.55 |
| HP:0001056 | Milia | (Abnormality of the integument ); | 1 | 0.55 |
| HP:0001602 | Laryngeal stenosis | (Abnormality of the respiratory system ); | 1 | 0.55 |
| HP:0002043 | Esophageal stricture | (Abnormality of the digestive system ); | 1 | 0.55 |
| HP:0005830 | Flexion contracture of toe | (Abnormality of the musculature );(Abnormality of limbs );(Abnormality of connective tissue );(Abnormality of the skeletal system ); | 1 | 0.55 |
| HP:0100758 | Gangrene | (Abnormality of metabolism/homeostasis ); | 1 | 0.55 |
| HP:0000202 | Oral cleft | (Abnormality of head and neck ); | 1 | 0.55 |
| HP:0000478 | Abnormality of the eye | (Abnormality of the eye ); | 1 | 0.55 |
| HP:0000504 | Abnormality of vision | (Abnormality of the eye ); | 1 | 0.55 |
| HP:0002557 | Hypoplastic nipples | (Abnormality of the breast ); | 1 | 0.55 |
| HP:0001337 | Tremor | (Abnormality of the nervous system ); | 1 | 0.55 |
| HP:0002354 | Memory impairment | (Abnormality of the nervous system ); | 1 | 0.55 |
| HP:0001987 | Hyperammonemia | (Abnormality of metabolism/homeostasis ); | 1 | 0.55 |
| HP:0001992 | Organic aciduria | (Abnormality of the genitourinary system );(Abnormality of metabolism/homeostasis ); | 1 | 0.55 |
| HP:0002098 | Respiratory distress | (Abnormality of the respiratory system ); | 1 | 0.55 |
| HP:0002789 | Tachypnea | (Abnormality of the respiratory system ); | 1 | 0.55 |
| HP:0000272 | Malar flattening | (Abnormality of head and neck );(Abnormality of the skeletal system ); | 1 | 0.55 |
| HP:0000520 | Proptosis | (Abnormality of head and neck );(Abnormality of the eye ); | 1 | 0.55 |
| HP:0000034 | Hydrocele testis | (Abnormality of the genitourinary system ); | 1 | 0.55 |
| HP:0000204 | Cleft upper lip | (Abnormality of head and neck ); | 1 | 0.55 |
| HP:0000510 | Retinitis pigmentosa | (Abnormality of the eye ); | 1 | 0.55 |
| HP:0000512 | Abnormal electroretinogram | (Abnormality of the eye ); | 1 | 0.55 |
| HP:0000529 | Progressive visual loss | (Abnormality of the eye ); | 1 | 0.55 |
| HP:0003100 | Slender long bone | (Abnormality of the skeletal system ); | 1 | 0.55 |
| HP:0005268 | Spontaneous abortion | (Abnormality of prenatal development or birth ); | 1 | 0.55 |
| HP:0000426 | Prominent nasal bridge | (Abnormality of head and neck ); | 1 | 0.55 |
| HP:0002342 | Intellectual disability, moderate | (Abnormality of the nervous system ); | 1 | 0.55 |
| HP:0002984 | Hypoplasia of the radius | (Abnormality of the skeletal system );(Abnormality of limbs ); | 1 | 0.55 |
| HP:0003022 | Hypoplasia of the ulna | (Abnormality of the skeletal system );(Abnormality of limbs ); | 1 | 0.55 |
| HP:0012368 | Flat face | (Abnormality of head and neck ); | 1 | 0.55 |
| HP:0000491 | Keratitis | (Abnormality of the eye );(Abnormality of the immune system ); | 3 | 0.45 |
| HP:0000498 | Blepharitis | (Abnormality of head and neck );(Abnormality of the immune system );(Abnormality of the eye ); | 3 | 0.45 |
| HP:0000509 | Conjunctivitis | (Abnormality of head and neck );(Abnormality of the immune system );(Abnormality of the eye ); | 3 | 0.45 |
| HP:0001744 | Splenomegaly | (Abnormality of the immune system );(Abnormality of the digestive system ); | 3 | 0.45 |
| HP:0000639 | Nystagmus | (Abnormality of the eye ); | 3 | 0.45 |
| HP:0001000 | Abnormality of skin pigmentation | (Abnormality of the integument ); | 3 | 0.45 |
| HP:0000656 | Ectropion | (Abnormality of head and neck ); | 3 | 0.45 |
| HP:0002808 | Kyphosis | (Abnormality of the skeletal system ); | 3 | 0.45 |
| HP:0002860 | Squamous cell carcinoma | (Abnormality of the integument );(Neoplasm ); | 3 | 0.45 |
| HP:0001287 | Meningitis | (Abnormality of the nervous system );(Abnormality of the immune system ); | 2 | 0.3 |
| HP:0100806 | Sepsis | (Abnormality of the immune system ); | 2 | 0.3 |
| HP:0100820 | Glomerulopathy | (Abnormality of the genitourinary system ); | 2 | 0.3 |
| HP:0000218 | High palate | (Abnormality of head and neck ); | 2 | 0.3 |
| HP:0007370 | aplasia/Hypoplasia of the corpus callosum | (Abnormality of the nervous system ); | 2 | 0.3 |
| HP:0000003 | Multicystic kidney dysplasia | (Abnormality of the genitourinary system ); | 2 | 0.3 |
| HP:0001161 | Hand polydactyly | (Abnormality of the skeletal system );(Abnormality of limbs ); | 2 | 0.3 |
| HP:0001274 | Agenesis of corpus callosum | (Abnormality of the nervous system ); | 2 | 0.3 |
| HP:0001650 | Aortic valve stenosis | (Abnormality of the cardiovascular system ); | 2 | 0.3 |
| HP:0004378 | Abnormality of the anus | (Abnormality of the digestive system ); | 2 | 0.3 |
| HP:0000091 | Abnormality of the renal tubule | (Abnormality of the genitourinary system ); | 2 | 0.3 |
| HP:0000691 | Microdontia | (Abnormality of head and neck ); | 2 | 0.3 |
| HP:0001631 | Defect in the atrial septum | (Abnormality of the cardiovascular system ); | 2 | 0.3 |
| HP:0004326 | Cachexia | (Growth abnormality ); | 2 | 0.3 |
| HP:0004372 | Reduced consciousness/confusion | (Abnormality of the nervous system ); | 2 | 0.3 |
| HP:0010807 | Open bite | (Abnormality of head and neck ); | 2 | 0.3 |
| HP:0100543 | Cognitive impairment | (Abnormality of the nervous system ); | 2 | 0.3 |
| HP:0100725 | Lichenification | (Abnormality of the integument ); | 2 | 0.3 |
| HP:0000405 | Conductive hearing impairment | (Abnormality of the ear ); | 2 | 0.3 |
| HP:0000722 | Obsessive-compulsive behavior | (Abnormality of the nervous system ); | 2 | 0.3 |
| HP:0000492 | Abnormality of the eyelid | (Abnormality of head and neck ); | 2 | 0.3 |
| HP:0100257 | Ectrodactyly | (Abnormality of the skeletal system );(Abnormality of limbs ); | 2 | 0.3 |
| HP:0005561 | Abnormality of bone marrow cell morphology | (Abnormality of blood and blood-forming tissues ); | 2 | 0.3 |
| HP:0007730 | Iris hypopigmentation | (Abnormality of the eye ); | 2 | 0.3 |
| HP:0002047 | Malignant hyperthermia | (Abnormality of metabolism/homeostasis ); | 2 | 0.3 |
| HP:0001254 | Lethargy | (Abnormality of the nervous system ); | 2 | 0.3 |
| HP:0001259 | Coma | (Abnormality of the nervous system ); | 2 | 0.3 |
| HP:0000112 | Nephropathy | (Abnormality of the genitourinary system ); | 1 | 0.15 |
| HP:0000140 | Abnormality of the menstrual cycle | (Abnormality of the genitourinary system );(Abnormality of the endocrine system ); | 1 | 0.15 |
| HP:0000421 | Epistaxis | (Abnormality of head and neck );(Abnormality of blood and blood-forming tissues ); | 1 | 0.15 |
| HP:0000778 | Hypoplasia of the thymus | (Abnormality of the endocrine system );(Abnormality of the immune system ); | 1 | 0.15 |
| HP:0001645 | Sudden cardiac death | (Abnormality of the cardiovascular system ); | 1 | 0.15 |
| HP:0002170 | Intracranial hemorrhage | (Abnormality of the nervous system );(Abnormality of the cardiovascular system );(Abnormality of blood and blood-forming tissues ); | 1 | 0.15 |
| HP:0002488 | Acute leukemia | (Abnormality of blood and blood-forming tissues );(Neoplasm );(Abnormality of the immune system ); | 1 | 0.15 |
| HP:0002633 | Vasculitis | (Abnormality of the cardiovascular system ); | 1 | 0.15 |
| HP:0005558 | Chronic leukemia | (Abnormality of blood and blood-forming tissues );(Neoplasm );(Abnormality of the immune system ); | 1 | 0.15 |
| HP:0006535 | Recurrent intrapulmonary hemorrhage | (Abnormality of the cardiovascular system );(Abnormality of blood and blood-forming tissues );(Abnormality of the respiratory system ); | 1 | 0.15 |
| HP:0009830 | Peripheral neuropathy | (Abnormality of the nervous system ); | 1 | 0.15 |
| HP:0011869 | Abnormal platelet function | (Abnormality of blood and blood-forming tissues ); | 1 | 0.15 |
| HP:0100749 | Chest pain | (Abnormality of the nervous system );(Abnormality of the skeletal system ); | 1 | 0.15 |
| HP:0100774 | Hyperostosis | (Abnormality of the skeletal system ); | 1 | 0.15 |
| HP:0100523 | Liver abscess | (Abnormality of the immune system );(Abnormality of the digestive system ); | 1 | 0.15 |
| HP:0000829 | Hypoparathyroidism | (Abnormality of the endocrine system ); | 1 | 0.15 |
| HP:0000960 | Sacral dimple | (Abnormality of the skeletal system );(Abnormality of the integument ); | 1 | 0.15 |
| HP:0000965 | Cutis marmorata | (Abnormality of the integument );(Abnormality of the cardiovascular system ); | 1 | 0.15 |
| HP:0001800 | Hypoplastic toenails | (Abnormality of the integument ); | 1 | 0.15 |
| HP:0001840 | Metatarsus adductus | (Abnormality of the skeletal system );(Abnormality of limbs ); | 1 | 0.15 |
| HP:0002025 | Anal stenosis | (Abnormality of the digestive system ); | 1 | 0.15 |
| HP:0002035 | Rectal prolapse | (Abnormality of the digestive system ); | 1 | 0.15 |
| HP:0006721 | Acute lymphatic leukemia | (Abnormality of blood and blood-forming tissues );(Neoplasm );(Abnormality of the immune system ); | 1 | 0.15 |
| HP:0000243 | Trigonocephaly | (Abnormality of head and neck );(Abnormality of the skeletal system ); | 1 | 0.15 |
| HP:0000465 | Webbed neck | (Abnormality of head and neck ); | 1 | 0.15 |
| HP:0000612 | Iris coloboma | (Abnormality of head and neck );(Abnormality of the eye ); | 1 | 0.15 |
| HP:0000625 | Cleft eyelid | (Abnormality of head and neck ); | 1 | 0.15 |
| HP:0001302 | Pachygyria | (Abnormality of the nervous system ); | 1 | 0.15 |
| HP:0001522 | Death in infancy | (Mortality/Aging ); | 1 | 0.15 |
| HP:0001680 | Coarctation of aorta | (Abnormality of the cardiovascular system ); | 1 | 0.15 |
| HP:0001734 | Annular pancreas | (Abnormality of the digestive system ); | 1 | 0.15 |
| HP:0001883 | Talipes | (Abnormality of limbs ); | 1 | 0.15 |
| HP:0002059 | Cerebral atrophy | (Abnormality of the nervous system ); | 1 | 0.15 |
| HP:0002247 | Duodenal atresia | (Abnormality of the digestive system ); | 1 | 0.15 |
| HP:0002414 | Spina bifida | (Abnormality of the nervous system ); | 1 | 0.15 |
| HP:0002566 | Intestinal malrotation | (Abnormality of the digestive system ); | 1 | 0.15 |
| HP:0002827 | Hip dislocation | (Abnormality of the skeletal system );(Abnormality of limbs ); | 1 | 0.15 |
| HP:0004383 | Hypoplastic left heart | (Abnormality of the cardiovascular system ); | 1 | 0.15 |
| HP:0007302 | Bipolar affective disorder | (Abnormality of the nervous system ); | 1 | 0.15 |
| HP:0100753 | Schizophrenia | (Abnormality of the nervous system ); | 1 | 0.15 |
| HP:0000541 | Retinal detachment | (Abnormality of the eye ); | 1 | 0.15 |
| HP:0000689 | Dental malocclusion | (Abnormality of head and neck ); | 1 | 0.15 |
| HP:0001083 | Ectopia lentis | (Abnormality of the eye ); | 1 | 0.15 |
| HP:0001119 | Keratoglobus | (Abnormality of the eye ); | 1 | 0.15 |
| HP:0001131 | Corneal dystrophy | (Abnormality of the eye ); | 1 | 0.15 |
| HP:0002076 | Migraine | (Abnormality of the nervous system ); | 1 | 0.15 |
| HP:0002107 | Pneumothorax | (Abnormality of the respiratory system ); | 1 | 0.15 |
| HP:0002101 | Abnormal lung lobation | (Abnormality of the respiratory system ); | 1 | 0.15 |
| HP:0100818 | Long thorax | (Abnormality of the skeletal system ); | 1 | 0.15 |
| HP:0000163 | Abnormality of the oral cavity | (Abnormality of head and neck ); | 1 | 0.15 |
| HP:0001541 | Ascites | (Abnormality of the digestive system ); | 1 | 0.15 |
| HP:0002243 | Protein-losing enteropathy | (Abnormality of the digestive system ); | 1 | 0.15 |
| HP:0003565 | Elevated erythrocyte sedimentation rate | (Abnormality of metabolism/homeostasis ); | 1 | 0.15 |
| HP:0002617 | Aneurysm | (Abnormality of the cardiovascular system ); | 1 | 0.15 |
| HP:0100658 | Cellulitis | (Abnormality of connective tissue ); | 1 | 0.15 |
| HP:0000086 | Ectopic kidney | (Abnormality of the genitourinary system ); | 1 | 0.15 |
| HP:0000653 | Sparse eyelashes | (Abnormality of head and neck );(Abnormality of the integument ); | 1 | 0.15 |
| HP:0001944 | Dehydration | (Abnormality of metabolism/homeostasis ); | 1 | 0.15 |
| HP:0003355 | Aminoaciduria | (Abnormality of the genitourinary system );(Abnormality of metabolism/homeostasis ); | 1 | 0.15 |
| HP:0000733 | Stereotypic behavior | (Abnormality of the nervous system ); | 1 | 0.15 |
| HP:0000776 | Congenital diaphragmatic hernia | (Abnormality of the musculature );(Abnormality of connective tissue ); | 1 | 0.15 |
| HP:0001601 | Laryngomalacia | (Abnormality of the respiratory system ); | 1 | 0.15 |
| HP:0001679 | Abnormality of the aorta | (Abnormality of the cardiovascular system ); | 1 | 0.15 |
| HP:0002360 | Sleep disturbance | (Abnormality of the nervous system ); | 1 | 0.15 |
| HP:0002667 | Nephroblastoma (Wilms tumor) | (Abnormality of the genitourinary system );(Neoplasm ); | 1 | 0.15 |
| HP:0002779 | Tracheomalacia | (Abnormality of the respiratory system ); | 1 | 0.15 |
| HP:0000077 | Abnormality of the kidney | (Abnormality of the genitourinary system ); | 1 | 0.15 |
| HP:0000453 | Choanal atresia | (Abnormality of head and neck ); | 1 | 0.15 |
| HP:0000554 | Uveitis | (Abnormality of the eye );(Abnormality of the immune system ); | 1 | 0.15 |
| HP:0000925 | Abnormality of the vertebral column | (Abnormality of the skeletal system ); | 1 | 0.15 |
| HP:0000926 | Platyspondyly | (Abnormality of the skeletal system ); | 1 | 0.15 |
| HP:0001321 | Cerebellar hypoplasia | (Abnormality of the nervous system ); | 1 | 0.15 |
| HP:0001331 | Absent septum pellucidum | (Abnormality of the nervous system ); | 1 | 0.15 |
| HP:0001539 | Omphalocele | (Abnormality of connective tissue );(Abnormality of the digestive system ); | 1 | 0.15 |
| HP:0002120 | Cerebral cortical atrophy | (Abnormality of the nervous system ); | 1 | 0.15 |
| HP:0002251 | Aganglionic megacolon | (Abnormality of the nervous system );(Abnormality of the digestive system ); | 1 | 0.15 |
| HP:0003468 | Abnormality of the vertebrae | (Abnormality of the skeletal system ); | 1 | 0.15 |
| HP:0010935 | Abnormality of the upper urinary tract | (Abnormality of the genitourinary system ); | 1 | 0.15 |
| HP:0012157 | Subcortical cerebral atrophy | (Abnormality of the nervous system ); | 1 | 0.15 |
| HP:0012165 | Oligodactyly | (Abnormality of the skeletal system );(Abnormality of limbs ); | 1 | 0.15 |
| HP:0040163 | Abnormal pelvis bone morphology | (Abnormality of the skeletal system ); | 1 | 0.15 |
| HP:0100308 | Cerebral cortical hemiatrophy | (Abnormality of the nervous system ); | 1 | 0.15 |
| HP:0100532 | Scleritis | (Abnormality of the eye );(Abnormality of the immune system ); | 1 | 0.15 |
| HP:0100534 | Episcleritis | (Abnormality of the eye );(Abnormality of the immune system ); | 1 | 0.15 |
| HP:0000771 | Gynecomastia | (Abnormality of the breast ); | 1 | 0.15 |
| HP:0000845 | Growth hormone excess | (Abnormality of the nervous system );(Abnormality of the endocrine system ); | 1 | 0.15 |
| HP:0000939 | Osteoporosis | (Abnormality of the skeletal system ); | 1 | 0.15 |
| HP:0000976 | Eczematoid dermatitis | (Abnormality of the integument );(Abnormality of the immune system ); | 1 | 0.15 |
| HP:0002239 | Gastrointestinal hemorrhage | (Abnormality of the cardiovascular system );(Abnormality of blood and blood-forming tissues );(Abnormality of the digestive system ); | 1 | 0.15 |
| HP:0002970 | Genu varum | (Abnormality of the skeletal system );(Abnormality of limbs ); | 1 | 0.15 |
| HP:0004398 | Peptic ulcer | (Abnormality of the digestive system ); | 1 | 0.15 |
| HP:0010829 | Impaired temperature sensation | (Abnormality of the nervous system ); | 1 | 0.15 |
| HP:0010885 | Aseptic necrosis | (Abnormality of the skeletal system ); | 1 | 0.15 |
| HP:0100021 | Cerebral palsy | (Abnormality of the nervous system ); | 1 | 0.15 |
| HP:0100526 | Neoplasm of the lungs | (Abnormality of the respiratory system );(Neoplasm ); | 1 | 0.15 |
| HP:0004299 | Hernia of the abdominal wall | (Abnormality of connective tissue );(Abnormality of the digestive system ); | 1 | 0.15 |
| HP:0000953 | Hyperpigmentation of the skin | (Abnormality of the integument ); | 1 | 0.15 |
| HP:0007605 | Excessive wrinkling of palmar skin | (Abnormality of the integument );(Abnormality of limbs ); | 1 | 0.15 |
| HP:0200041 | Skin erosion | (Abnormality of the integument ); | 1 | 0.15 |
| HP:0000211 | Trismus | (Abnormality of head and neck );(Abnormality of the skeletal system ); | 1 | 0.15 |
| HP:0000772 | Abnormality of the ribs | (Abnormality of the skeletal system ); | 1 | 0.15 |
| HP:0000795 | Abnormality of the urethra | (Abnormality of the genitourinary system ); | 1 | 0.15 |
| HP:0000924 | Abnormality of the skeletal system | (Abnormality of the skeletal system ); | 1 | 0.15 |
| HP:0006740 | Transitional cell carcinoma of the bladder | (Abnormality of the genitourinary system );(Neoplasm ); | 1 | 0.15 |
| HP:0007759 | Opacification of the corneal stroma | (Abnormality of the eye ); | 1 | 0.15 |
| HP:0005616 | Accelerated skeletal maturation | (Abnormality of the skeletal system ); | 1 | 0.15 |
| HP:0000821 | Hypothyroidism | (Abnormality of the endocrine system ); | 1 | 0.15 |
| HP:0001116 | Macular coloboma | (Abnormality of head and neck );(Abnormality of the eye ); | 1 | 0.15 |
| HP:0001654 | Abnormality of the heart valves | (Abnormality of the cardiovascular system ); | 1 | 0.15 |
| HP:0001939 | Abnormality of metabolism/homeostasis | (Abnormality of metabolism/homeostasis ); | 1 | 0.15 |
| HP:0100777 | Exostoses | (Abnormality of the skeletal system );(Neoplasm ); | 1 | 0.15 |
| HP:0006763 | Anal canal squamous carcinoma | (Neoplasm );(Abnormality of the digestive system ); | 1 | 0.15 |
| HP:0030417 | Squamous cell carcinoma of the vulva | (Abnormality of the genitourinary system );(Neoplasm ); | 1 | 0.15 |
| HP:0001055 | Erysipelas | (Abnormality of the integument );(Abnormality of the immune system ); | 1 | 0.15 |
| HP:0100783 | Breast aplasia | (Abnormality of the breast ); | 1 | 0.15 |
| HP:0000179 | Thick lower lip vermilion | (Abnormality of head and neck ); | 1 | 0.15 |
| HP:0000358 | Posteriorly rotated ears | (Abnormality of the ear ); | 1 | 0.15 |
| HP:0000444 | Convex nasal ridge | (Abnormality of head and neck ); | 1 | 0.15 |
| HP:0000574 | Thick eyebrow | (Abnormality of head and neck );(Abnormality of the integument ); | 1 | 0.15 |
| HP:0000718 | Aggressive behavior | (Abnormality of the nervous system ); | 1 | 0.15 |
| HP:0002003 | Large forehead | (Abnormality of head and neck ); | 1 | 0.15 |
| HP:0003186 | Inverted nipples | (Abnormality of the breast ); | 1 | 0.15 |
| HP:0011246 | Underdeveloped superior crus of antihelix | (Abnormality of the ear ); | 1 | 0.15 |
| HP:0001288 | Gait disturbance | (Abnormality of the nervous system ); | 1 | 0.15 |
| HP:0001909 | Leukemia | (Abnormality of blood and blood-forming tissues );(Neoplasm );(Abnormality of the immune system ); | 1 | 0.15 |
| HP:0001915 | aplastic anemia | (Abnormality of blood and blood-forming tissues ); | 1 | 0.15 |
| HP:0002863 | Myelodysplasia | (Abnormality of blood and blood-forming tissues );(Neoplasm ); | 1 | 0.15 |
| HP:0004808 | Acute myeloid leukemia | (Abnormality of blood and blood-forming tissues );(Neoplasm );(Abnormality of the immune system ); | 1 | 0.15 |
| HP:0011107 | Recurrent aphthous stomatitis | (Abnormality of head and neck );(Abnormality of the immune system ); | 1 | 0.15 |
| HP:0002516 | Increased intracranial pressure | (Abnormality of the nervous system ); | 1 | 0.15 |
| HP:0004349 | Reduced bone mineral density | (Abnormality of the skeletal system ); | 1 | 0.15 |
| HP:0000568 | Microphthalmos | (Abnormality of head and neck );(Abnormality of the eye ); | 1 | 0.15 |
| HP:0004408 | Abnormality of the sense of smell | (Abnormality of head and neck );(Abnormality of the nervous system ); | 1 | 0.15 |
| HP:0001655 | Patent foramen ovale | (Abnormality of the cardiovascular system ); | 1 | 0.15 |
| HP:0001904 | Autoimmune neutropenia | (Abnormality of blood and blood-forming tissues );(Abnormality of the immune system ); | 1 | 0.15 |
| HP:0001973 | Autoimmune thrombocytopenia | (Abnormality of blood and blood-forming tissues );(Abnormality of the immune system ); | 1 | 0.15 |
| HP:0002092 | Pulmonary hypertension | (Abnormality of the respiratory system ); | 1 | 0.15 |
| HP:0002383 | Encephalitis | (Abnormality of the nervous system ); | 1 | 0.15 |
| HP:0002724 | Recurrent Aspergillus infections | (Abnormality of the immune system ); | 1 | 0.15 |
| HP:0003613 | Antiphospholipid antibody positivity | (Abnormality of the immune system ); | 1 | 0.15 |
| HP:0004966 | Medial calcification of large arteries | (Abnormality of the skeletal system );(Abnormality of the cardiovascular system ); | 1 | 0.15 |
| HP:0011459 | Esophageal carcinoma | (Neoplasm );(Abnormality of the digestive system ); | 1 | 0.15 |
| HP:0012115 | Hepatitis | (Abnormality of the immune system );(Abnormality of the digestive system ); | 1 | 0.15 |
| HP:0012182 | Oropharyngeal squamous cell carcinoma | (Abnormality of the integument );(Neoplasm ); | 1 | 0.15 |
| HP:0030355 | Abnormal serum interferon-gamma level | (Abnormality of the immune system ); | 1 | 0.15 |
| HP:0000016 | Urinary retention | (Abnormality of the genitourinary system ); | 1 | 0.15 |
| HP:0000071 | Ureteral stenosis | (Abnormality of the genitourinary system ); | 1 | 0.15 |
| HP:0000083 | Renal insufficiency | (Abnormality of the genitourinary system ); | 1 | 0.15 |
| HP:0000579 | Nasolacrimal duct obstruction | (Abnormality of head and neck ); | 1 | 0.15 |
| HP:0001297 | Stroke | (Abnormality of the nervous system );(Abnormality of the cardiovascular system ); | 1 | 0.15 |
| HP:0001644 | Dilated cardiomyopathy | (Abnormality of the cardiovascular system ); | 1 | 0.15 |
| HP:0001741 | Phimosis | (Abnormality of the genitourinary system ); | 1 | 0.15 |
| HP:0006530 | Interstitial pulmonary disease | (Abnormality of the respiratory system ); | 1 | 0.15 |
| HP:0012451 | Acute constipation | (Abnormality of the digestive system ); | 1 | 0.15 |
| HP:0100699 | Scarring | (Abnormality of connective tissue ); | 1 | 0.15 |
| HP:0000572 | Visual loss | (Abnormality of the eye ); | 1 | 0.15 |
| HP:0003777 | Pili torti | (Abnormality of the integument ); | 1 | 0.15 |
| HP:0007447 | Diffuse palmoplantar hyperkeratosis | (Abnormality of the integument );(Abnormality of limbs ); | 1 | 0.15 |
| HP:0012384 | Rhinitis | (Abnormality of head and neck ); | 1 | 0.15 |
| HP:0100776 | Recurrent pharyngitis | (Abnormality of head and neck );(Abnormality of the respiratory system );(Abnormality of the immune system ); | 1 | 0.15 |
| HP:0001347 | Hyperreflexia | (Abnormality of the nervous system ) | 1 | 0.15 |
| HP:0002301 | Hemiplegia | (Abnormality of the nervous system ); | 1 | 0.15 |
| HP:0002333 | Motor deterioration | (Abnormality of the nervous system ); | 1 | 0.15 |
| HP:0002514 | Cerebral calcification | (Abnormality of the nervous system );(Abnormality of the skeletal system ); | 1 | 0.15 |
| HP:0010550 | Paraplegia | (Abnormality of the nervous system ); | 1 | 0.15 |
| HP:0100716 | Self-injurious behavior | (Abnormality of the nervous system ); | 1 | 0.15 |
| HP:0001123 | Visual field defect | (Abnormality of the eye ); | 1 | 0.15 |
| HP:0001317 | Abnormality of the cerebellum | (Abnormality of the nervous system ); | 1 | 0.15 |
| HP:0002104 | Apnea | (Abnormality of the respiratory system ); | 1 | 0.15 |
| HP:0002841 | Recurrent fungal infections | (Abnormality of the immune system ); | 1 | 0.15 |
| HP:0002883 | Hyperventilation | (Abnormality of the respiratory system ); | 1 | 0.15 |
| HP:0006511 | Laryngeal stridor | (Abnormality of the respiratory system ); | 1 | 0.15 |
| HP:0001081 | Cholelithiasis | (Abnormality of the digestive system ); | 1 | 0.15 |
| HP:0001394 | Cirrhosis | (Abnormality of the digestive system ); | 1 | 0.15 |
| HP:0001410 | Decreased liver function | (Abnormality of the digestive system ); | 1 | 0.15 |
| HP:0002194 | Delayed gross motor development | (Abnormality of the nervous system ); | 1 | 0.15 |
| HP:0002805 | Accelerated bone age after puberty | (Abnormality of the skeletal system ); | 1 | 0.15 |
| HP:0008258 | Congenital adrenal hyperplasia | (Abnormality of the endocrine system ); | 1 | 0.15 |
| HP:0008665 | Clitoral hypertrophy | (Abnormality of the genitourinary system ); | 1 | 0.15 |
| HP:0030088 | Increased serum testosterone level | (Abnormality of the endocrine system );(Abnormality of metabolism/homeostasis ); | 1 | 0.15 |
| HP:0000567 | Chorioretinal coloboma | (Abnormality of head and neck );(Abnormality of the eye ); | 1 | 0.15 |
| HP:0001651 | Dextrocardia | (Abnormality of the cardiovascular system ); | 1 | 0.15 |
| HP:0009777 | Absent thumb | (Abnormality of the skeletal system );(Abnormality of limbs ); | 1 | 0.15 |
| HP:0011466 | aplasia/Hypoplasia of the gallbladder | (Abnormality of the digestive system ); | 1 | 0.15 |

# the count means how many rare diseases the concomitant phenotype shared with eczema.

*the score was calculated based on the frequency of phenotype in different rare diseases. Five weight value were used 0.9999 for obligate (100%) phenotype; 0.9000 for very frequent (99-80%) phenotypes; 0.5500 for frequent (79-30%) phenotypes; 0.1500 for occasional (29-5%) phenotypes; 0.0200 for very rare (<4-1%) phenotypes.


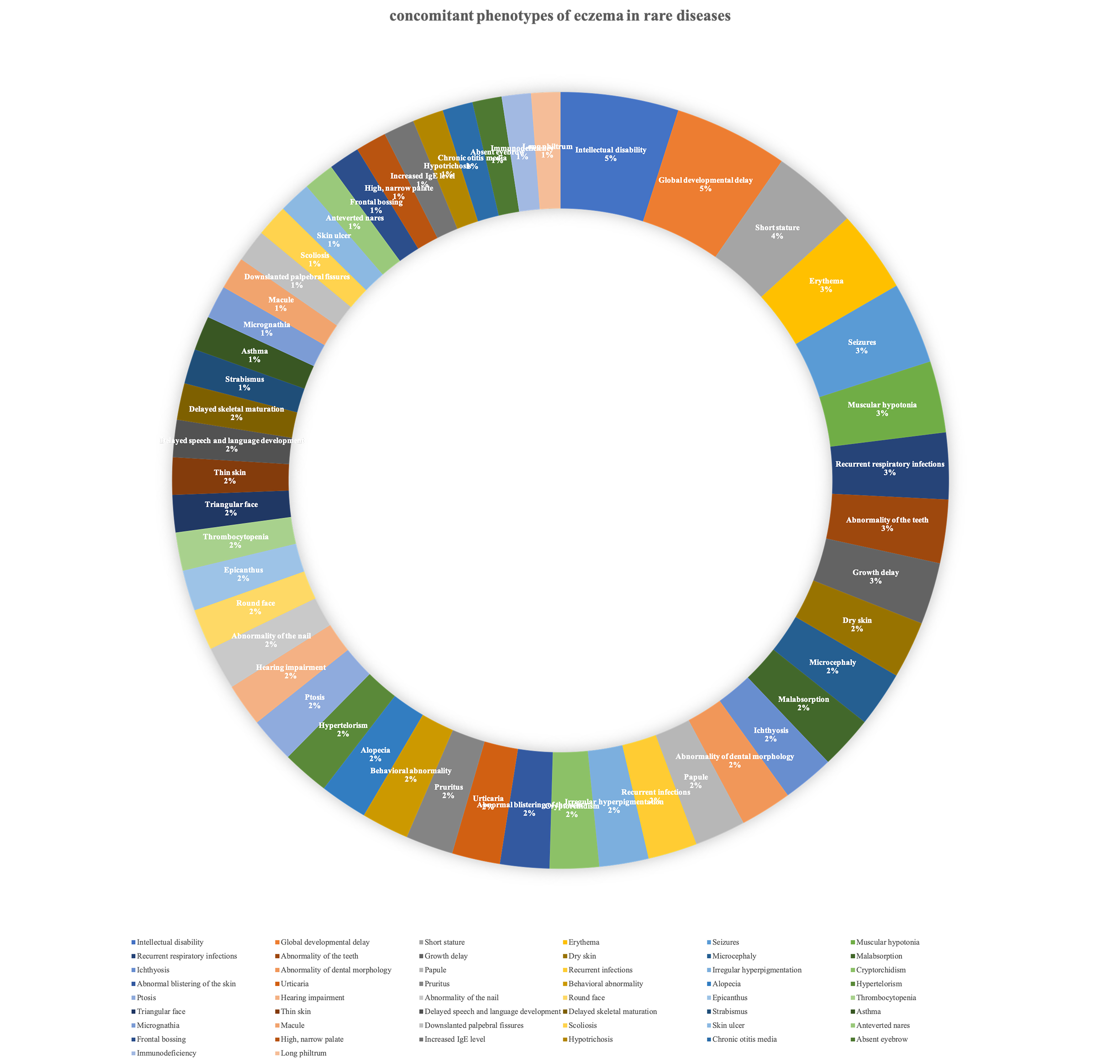


**Figure S1 Top 50 concomitant phenotypes of eczema in rare diseases.**
